# Starvation alters the liver transcriptome of the innate immune response in Atlantic salmon (*Salmo salar*)

**DOI:** 10.1186/1471-2164-11-418

**Published:** 2010-07-05

**Authors:** Samuel AM Martin, Alex Douglas, Dominic F Houlihan, Christopher J Secombes

**Affiliations:** 1 Scottish Fish Immunology Research Centre, Institute of Biological and Environmental Sciences, University of Aberdeen, Tillydrone Avenue, Aberdeen, AB24 2TZ, UK

## Abstract

**Background:**

The immune response is an energy demanding process, which has effects in many physiological pathways in the body including protein and lipid metabolism. During an inflammatory response the liver is required to produce high levels of acute phase response proteins that attempt to neutralise an invading pathogen. Although this has been extensively studied in both mammals and fish, little is known about how high and low energy reserves modulate the response to an infection in fish which are ectothermic vertebrates. Food withdrawal in fish causes a decrease in metabolic rate so as to preserve protein and lipid energy reserves, which occurs naturally during the life cycle of many salmonids. Here we investigated how the feeding or fasting of Atlantic salmon affected the transcriptional response in the liver to an acute bacterial infection.

**Results:**

Total liver RNA was extracted from four different groups of salmon. Two groups were fed or starved for 28 days. One of each of the fed or starved groups was then exposed to an acute bacterial infection. Twenty four hours later (day 29) the livers were isolated from all fish for RNA extraction. The transcriptional changes were examined by micro array analysis using a 17 K Atlantic salmon cDNA microarray. The expression profiling results showed major changes in gene transcription in each of the groups. Enrichment for particular biological pathways was examined by analysis of gene ontology. Those fish that were starved decreased immune gene transcription and reduced production of plasma protein genes, and upon infection there was a further decrease in genes encoding plasma proteins but a large increase in acute phase response proteins. The latter was greater in magnitude than in the fish that had been fed prior to infection. The expression of several genes that were found altered during microarray analysis was confirmed by real time PCR.

**Conclusions:**

We demonstrate that both starvation and infection have profound effects on transcription in the liver of salmon. There was a significant effect on the transcriptional response to infection depending on the prior feeding regime of the fish. It is likely that the energy demands on protein synthesis for acute phase response proteins are relatively high in the starved fish which have reduced energy reserves. This has implications for dietary control of fish if an immune response is anticipated.

## Background

The immune response is a coordinated reaction mounted by a host in an attempt to control or destroy an invading pathogen. There are a multitude of different processes that occur during this response which are dependent on the type of pathogen, the route of infection or the previous exposure to the pathogen. The innate immune response includes both cellular and humoral elements. These can detect and neutralise pathogens [1] but manage to avoid attacking the hosts own tissues. Once an inflammatory immune response is initiated proinflammatory cytokines including IL-1β, IL-6, tumour necrosis factor α and a panel of chemokines instigate the coordinated expression of downstream genes. Fish have an increasingly well characterised innate immune system [2] with many genes being identified as having a role in controlling the immune response [3]. Transcriptome analysis has been performed in salmonid fish following bacterial [4-7], viral [8-10] and parasitic infections [11].

Mounting an immune response requires energy and an increase in metabolic activity, and the effectiveness of the response may be related to body energy reserves [12]. In mammals dietary restriction can be advantageous in relation to autoimmune diseases [13] but is deleterious with respect to defence to infections [14]. In many temperate animals allocation of energy reserves change on a seasonal basis resulting in altered immune status, as seen in deer mice where B cell production is decreased especially during energy demanding periods [15]. Repression of the immune response can be linked to survival in animals that have a high energy reserve demand. For example eider ducks showing a low humoral immune reactivity have significantly greater return rates to breeding areas than those with high immune activity [16].

Although nutritional status of individuals has been shown to have direct effects on their immune response these studies have focused primarily on human health [17] and endothermic animals[18]. Dramatic effects on the transcriptional response in mammals result from either complete food restriction [19] or from calorie restriction [20] with multiple tissues responding. Mice starved for 24 h show activation of genes related to muscle wastage [21] releasing free amino acids for essential metabolic functions. These same pathways are activated during chronic infection [22], demonstrating a link between nutritional status and immune function.

Fish are ectothermic and as such control their metabolic rate very differently to mammals. They show major changes following starvation [23-25] such as a generalized reduction in protein synthesis and protein turnover [26], however almost nothing is known about the effect of dietary restriction on the immune response in fish. Additionally carnivorous fish such as salmonids rely on protein as the major energy source oxidising proteins for gluconeogenesis [27]. As the liver is a central organ controlling many physiological functions and synthesizing plasma proteins, under acute infection conditions it is required to alter much of its transcriptional and translational machinery to synthesise high levels of acute phase reactants [28,29]. The production of these acute phase proteins requires increased access to free amino acids. Additionally protein synthesis is an energy demanding process [30] thus mounting of a successful innate response may be related to the availability of sufficient energy reserves and free amino acids for de novo synthesis.

In this paper we have examined how the liver transcriptional response to a bacterial pathogen of Atlantic salmon (*Salmo salar*) is modulated depending on the previous feeding regime. We have used the Atlantic salmon TRAITS-SGP 17 K cDNA microarray [31,32] platform that has been enriched for genes related to lipid and protein metabolism and the immune response [5]. Our results show that key components of the immune system are depressed during starvation but that following infection the starved fish attempt to compensate for this by increasing expression of several key immune related genes to a much greater extent than that seen in fish fed prior to infection.

## Results

All fish survived the 4 week trial. The mean (± sem) size of fish starting the trial was 54.2g ± 1.3 and the growth rate of the fed fish was 0.87% ± 0.04 body weight (bw) day^-1^. Fish that were starved lost weight at -0.57% ± 0.05 bw day^-1^. The condition factor of the starved fish was also significantly decreased compared to fed fish (0.96 ± 0.02 and 1.27 ± 0. 17, P < 0.01) at the end of the experiment, indicating a decreased level of energy reserves in the starved fish. The condition factor for the fed fish increased significantly during the experiment, increasing from 1.16 ± 0.01 to 1.27 ±0.17 (P < 0.01), showing that the fish were depositing additional energy reserves during growth.

### Overview of transcriptome analysis

**Figure 1:**
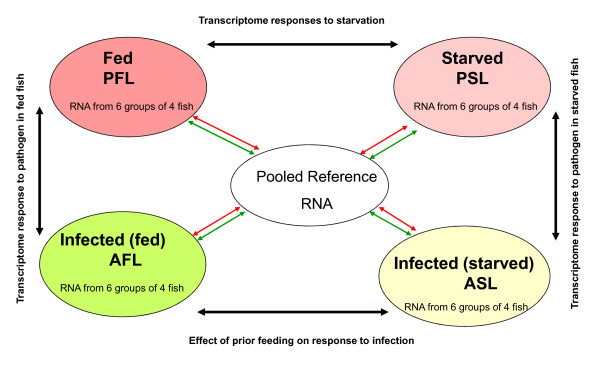
**Experimental design and hybridizations**. Diagram outlining the experimental setup and hybridization strategy for microarray analysis. For each experimental group 6 pools of liver RNA extracted from 4 individuals was used. Experimental groups were: PBS control/fed liver (PFL), from fish fed a normal diet and injected with PBS: PBS control/starved liver (PSL), fish starved for 28 days and injected with PBS: *Aeromonas salmonicida *infected/fed liver (AFL), fish fed normal diet and injected with 100 μL of bacteria (*A. salmonicida*, 10^9 ^CFU ml^-1^); *A. salmonicida *infected/starved liver (ASL) fish starved for 28 days and injected with 100 μL of bacteria (*A. salmonicida*, 10^9 ^CFU ml^-1^). All fish were sampled 24 h following injection. The pooled control was an equal mixture of RNA pools from each group. All hybridizations were between experimental groups and the pooled control. For each group of 6, 12 hybridizations were performed which included a dye swap.

The four groups of fish allowed examination of the changes in the liver transcriptome occurring as a result of starvation and acute bacterial infection. Additionally we wished to determine the effect of infection on the liver transcriptome depending on the feeding regime prior to infection. For each sampling, 6 replicates of fish were used, each replicate consisting of four individual fish (24 fish per group). RNA extracted from 4 different fish was pooled equally and then considered as a single sample. This pooling of individuals removed any large individual variation as our intention was not to study individual responses. The experimental design (Fig. 1) was a common reference design for which the reference sample was an equi molar mixture of all the experimental samples and cDNA from the experimental groups was hybridized against this common control RNA sample. Each of the 6 replicates was hybridized using a dye swap protocol resulting in 12 slides per group and 48 slides in total. All 48 microarray slides hybridized were used in the analysis. Following scanning of the slides and quality control 9855 (58%) cDNA features reached a threshold and were maintained for analysis as described in the materials and methods.

**Figure 2:**
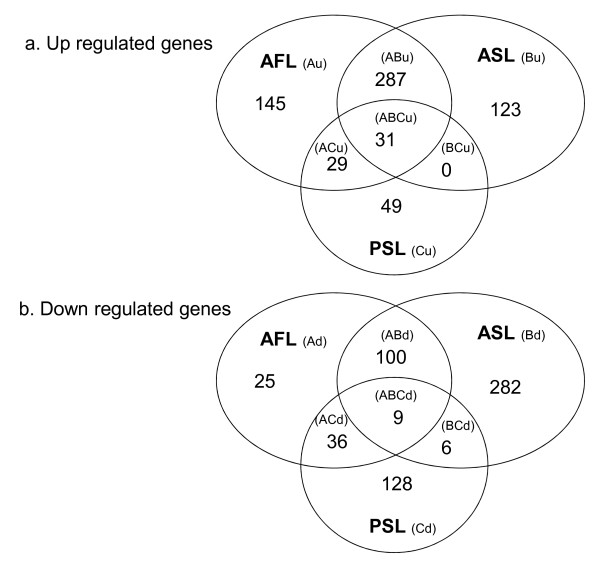
**Venn diagram showing numbers of genes identified as differentially expressed by microarray analysis**. Summary of numbers of genes up and down regulated. The genes presented are all significantly altered in expression (P < 0.001 and multiple test correction) with >2 fold change in expression. Up regulated in AFL group relative to PFL is also denoted as Au, upregulated in ASL relative to PSL is denoted as Bu, up regulated in PSL relative to PFL is denoted as Cu, and down regulated genes are Ad, Bd and Cd respectively.

The challenges resulted in large global alterations in transcriptional activity, with 2363 genes being significantly altered following infection compared to 959 genes that were altered by the starvation, indicating that 23.9% and 9.7% of those genes that passed filtering were significantly affected by these treatments. The numbers of genes differentially expressed are shown in Fig. 2. For annotation of the microarray, 76% of the genes could be assigned to a functional protein and 46% of the genes had a gene ontology (GO) identifier. This allowed statistical analysis for enrichment for GO biological processes to help interpret the changes in the transcriptome following starvation, infection and the effect of feeding regime on the response to infection.

### Transcriptome changes following starvation

Of 959 genes significantly altered by starvation, 288 were altered by greater than 2 fold, with 109 showing an increased and 179 a decreased expression as a result of starvation (Table 1 & Additional file 1 Table S1). To further interpret this extensive list of genes, enrichment for GO biological process was performed. Seventy one biological processes were found to be significantly altered following starvation. The GO enrichments indicated major alterations in biological processes related to fatty acid and steroid metabolism, energy metabolism, cell structure, immune status, protein metabolism and ion transport (Table 2, Additional file 2 Table S2). Genes encoding proteins related to a number of key biological processes that were altered in expression following starvation are outlined below.

**Table 1:** Genes altered significantly in liver by starvation

TRAITS IDENTIFIER^1^	ACC^2^	AFL^3^	ASL^4^	PSL^5^		Identity^6^
		FC A	FC B	FC C	group^7^	
**Blood and serum proteins**						
liv_stb_J4D11_sti_tra_sub_0p_11S	AM397499			3.17	Cu	(P80961) Antifreeze protein LS-12 precursor
liv_ali_02H02_abe_tra_sub_0p_11C	AM402609			2.92	Cu	Type-4 ice-structuring protein precursor (Antifreeze protein type IV)
tes_opk_14E17_osl_sgp_std_5p_11S	CK899203			2.26	Cu	(P29788) Vitronectin precursor (Serum spreading factor) (S-protein)
						
int_oss_T5O22_osl_sal_std_5p_11C	CK885870		-2.15	-2.74	BCd	(P14527) Hemoglobin alpha-4 subunit (Hemoglobin alpha-4 chain) (Alpha-4-globin)
spl_sts_19B11_sti_sal_std_5p_12S	AJ425729			-3.71	Cd	(P02019) Hemoglobin alpha-1 subunit (Hemoglobin alpha-1 chain) (Alpha-1-globin)
mus_snm_10F04_osl_tra_nrc_5p_11C	EG648831			-4.16	Cd	(P02142) Hemoglobin beta-1 subunit
swi_rpk_74B22_osl_sgp_std_5p_11C	CK896319			-6.95	Cd	(Q01584) Lipocalin precursor
						
**Energy metabolism**						
swi_rpk_74M05_osl_sgp_std_5p_11S	CK895001			3.68	Cu	isocitrate dehydrogenase 2 (NADP+) mitochondrial
gil_oss_52N23_osl_sal_std_5p_11C	CK879377			3.05	Cu	(Q9N2D5) Glyceraldehyde-3-phosphate dehydrogenase (EC 1.2.1.12) (GAPDH)
liv_ali_06G02_abe_tra_sub_0p_11S	AM402930			2.05	Cu	Bos taurus mono(ADP-ribosyl)transferase (ART1) mRNA
int_oss_T5A05_osl_sal_std_5p_11C	CK885697			-2.17	Cd	(P48163) NADP-dependent malic enzyme (EC 1.1.1.40) (NADP-ME) (Malic enzyme1)
pit_cpi_C1C04_car_tra_sub_0p_11C	No Acc			-2.39	Cd	glyoxalase 1
tes_tsr_02H11_gal_sal_std_5p_11C	BM413811			-3.06	Cd	(Q8BH95) Enoyl-CoA hydratase mitochondrial precursor (EC 4.2.1.17)
						
**Stress response**						
kid_aki_05A08_abe_tra_sub_0p_11C	AM042306	29.57	12.79	3.63	ABCu	(P06761) 78 kDa glucose-regulated protein precursor (GRP 78) (BiP)
hrt_opk_04E05_osl_sgp_std_5p_11C	CK883188	15.54	7.86	2.57	ABCu	DnaJ-like subfamily B member 11
gil_oss_GHB16_osl_sal_std_5p_11C	CN181053			2.30	Cu	(P08110) Endoplasmin precursor (Heat shock 108 kDa protein) (Transferrin-binding protein)
int_oss_T5B18_osl_sal_std_5p_11C	CK885962	2.31		2.27	ACu	(P30710) Epididymal secretory glutathione peroxidase precursor (EC 1.11.1.9)
liv_opk_12F09_osl_sgp_std_5p_11C	CK888938			2.12	Cu	glutathione peroxidase 3
int_oss_T4K02_osl_sal_std_5p_11C	CK884637			2.10	Cu	(O57521) Heat shock protein HSP 90-beta
gil_rpk_76A21_osl_sgp_std_5p_11C	CK878328			2.06	Cu	(P08294) Extracellular superoxide dismutase [Cu-Zn] precursor (EC 1.15.1.1)
hrt_opk_08M01_osl_sgp_std_5p_11C	CK899423	-2.39		-2.47	ACd	thioredoxin interacting protein
bra_snb_06F02_osl_tra_nrc_5p_11C	EG647964			-2.84	Cd	(Q4AEH7) Glutathione peroxidase 2 (EC 1.11.1.9)
						
**Immune related**						
swi_rpk_74F07_osl_sgp_std_5p_11C	CK896920			9.77	Cu	(Q9ES30) Complement C1q tumor necrosis factor-related protein 3 precursor
liv_ali_02G01_abe_tra_sub_0p_11C	AM402595			5.33	Cu	C-type MBL-2 protein
liv_lrr_01C04_gal_sal_std_5p_11C	No Acc			2.91	Cu	(Q02988) Lectin precursor
eye_opk_20I02_osl_sgp_std_5p_11S	CO471610	7.56	2.97	2.86	ABCu	(P27797) Calreticulin precursor (CRP55) (Calregulin)
liv_dis_D1E12_abe_tra_sub_0p_11N	AM049479	16.18	6.74	2.79	ABCu	similar to catechol-O-methyltransferase domain containing 1
liv_dis_D4H02_abe_tra_sub_0p_11N	AM049741	14.11	7.30	2.59	ABCu	precerebellin-like protein
liv_dis_D4B06_abe_tra_sub_0p_11N	AM049686			2.04	Cu	(Q9WUW3) Complement factor I precursor (EC 3.4.21.45) (C3B/C4B inactivator)
liv_dis_D1G04_abe_tra_sub_0p_11N	AM049505	2.65		2.00	ACu	(O88803) Leukocyte cell-derived chemotaxin 2 precursor
kid_aki_05H02_abe_tra_sub_0p_11C	AM042371			-2.03	Cd	(Q801Y3) Hepcidin 1 precursor
tes_tsa_01C01_gal_sal_std_5p_11C	No Acc			-2.58	Cd	liver-expressed antimicrobial peptide 2 isoform A precursor
liv_lrr_01C06_gal_sal_std_5p_11C	No Acc.	-2.05		-2.59	ACd	(P04186) Complement factor B precursor (EC 3.4.21.47)
liv_dis_D4A07_abe_tra_sub_0p_11N	AM049678			-3.09	Cd	C type lectin receptor A
eye_rpk_73M16_osl_sgp_std_5p_11C	CK875442			-3.82	Cd	TTRAP_BRARETRAF and TNF receptor-associated protein homolog
gil_oss_G6N16_osl_sal_std_5p_11C	CK877483	-2.31		-4.15	ACd	nuclear factor kappa-B 1
liv_opk_12I14_osl_sgp_std_5p_11S	CK889507			-4.65	Cd	(P80429) Serotransferrin II precursor (Siderophilin II) (STF II)
kid_aki_04G07_abe_tra_sub_0p_11C	AM042284			-10.17	Cd	(P81491) Serum amyloid A-5 protein
						
**Lipid and steroid metabolism**						
bra_opk_07F18_osl_sgp_std_5p_11S	CK875291	5.39		4.62	ACu	(O35760) Isopentenyl-diphosphate delta-isomerase 1 (EC 5.3.3.2)
swi_rpk_74J22_osl_sgp_std_5p_11C	CK896123			3.91	Cu	similar to Elongation of very long chain fatty acids protein 2
liv_opk_12J23_osl_sgp_std_5p_11C	CK888142	4.94		3.35	ACu	AF232215Salvelinus fontinalis steroidogenic acute regulatory protein (StAR)
hrt_opk_06O21_osl_sgp_std_5p_11C	CK874989			3.13	Cu	(O88822) Lathosterol oxidase (EC 1.3.3.2) (Lathosterol 5-desaturase)
bra_snb_09B09_osl_tra_nrc_5p_11S	EG647637			2.77	Cu	squalene monooxygenase
liv_ali_01E09_abe_tra_sub_0p_11S	AM402497			2.74	Cu	(P23228) Hydroxymethylglutaryl-CoA synthase cytoplasmic (EC 2.3.3.10)
bra_bfo_02D04_fou_sal_nrc_5p_11S	DW588272			2.36	Cu	(P37268) Squalene synthetase (EC 2.5.1.21)
kid_opk_01A09_osl_sgp_std_5p_11S	CK887294			-2.18	Cd	(O54939) Estradiol 17-beta-dehydrogenase 3 (EC 1.1.1.62)
bra_bfo_13G03_fou_sal_nrp_5p_11M	DW589986			-4.35	Cd	(Q92038) Acyl-CoA desaturase (EC 1.14.19.1) (Delta(9)-desaturase)
ova_opk_10D15_osl_sgp_std_5p_11C	CK891273	-5.23		-4.51	ACd	(Q18268) Phosphodiesterase delta-like protein
						
**Amino acid metabolism**						
swi_rpk_74K12_osl_sgp_std_5p_11C	CK895973			2.74	Cu	betaine-homocysteine methyltransferase
bra_bfo_15H06_fou_sal_nrp_5p_11M	DW590158			2.73	Cu	(P46410) Glutamine synthetase (EC 6.3.1.2) (Glutamate--ammonia ligase) (GS)
liv_ali_03E06_abe_tra_sub_0p_11C	AM402665			2.63	Cu	alanine-glyoxylate aminotransferase
mus_snm_07H11_osl_tra_nrc_5p_11C	EG648581			2.63	Cu	(P82264) GLutamate dehydrogenase (EC 1.4.1.3) (GDH)
liv_ali_02E10_abe_tra_sub_0p_11C	AM402574			2.13	Cu	(Q866Y3) N-acetylmuramoyl-L-alanine amidase precursor (EC 3.5.1.28)
liv_opk_12F15_osl_sgp_std_5p_11C	CK888977			2.00	Cu	(P16930) Fumarylacetoacetase (EC 3.7.1.2) (Fumarylacetoacetate hydrolase)
						
**Protein degradation**						
ova_oyr_02D04_gal_sal_std_5p_11S	BM414061	4.13	2.09	4.07	ABCu	(P97571) Calpain-1 catalytic subunit (EC 3.4.22.52)
mus_mfo_14E10_fou_sal_nrp_5p_11M	DW592075			-2.12	Cd	(Q9JHW0) Proteasome subunit beta type 7 precursor (EC 3.4.25.1)
liv_ali_04H04_abe_tra_sub_0p_11C	AM402779			-2.22	Cd	(P62193) 26S protease regulatory subunit 4 (Proteasome 26S subunit ATPase 1)
hkd_opk_03C15_osl_sgp_std_5p_11S	CK881845			-2.31	Cd	(P18242) Cathepsin D precursor (EC 3.4.23.5)
spl_sts_03D12_sti_sal_std_5p_11C	AJ425020			-2.43	Cd	(Q9H944) Ubiquitin-specific protease homolog 49 (TRF-proximal protein homolog)
tes_opk_13P17_osl_sgp_std_5p_11S	CK898961	-2.99		-3.60	ACd	(Q92743) Serine protease HTRA1 precursor (EC 3.4.21.-)
int_oss_T6A14_osl_sal_std_5p_11C	CK884021			-3.66	Cd	(P62972) Ubiquitin
int_rpk_78A08_osl_sgp_std_5p_11S	CK885620	-7.97		-8.17	ACd	(Q04592) Proprotein convertase subtilisin/kexin type 5 precursor (EC 3.4.21.-)
						
**Protein synthesis**						
gil_rpk_75O01_osl_sgp_std_5p_11C	CK878136			-2.29	Cd	(P47198) 60S ribosomal protein L22
gil_cgi_E3H04_car_tra_sub_0p_11N	No Acc	-2.39		-2.40	ACd	(Q9DCH4) Eukaryotic translation initiation factor 3 subunit 5
bra_bfo_04H04_fou_sal_nrc_3p_11C	DW588554			-2.45	Cd	AF390021_1glutamine synthetase
mus_mfo_13G09_fou_sal_nrp_5p_11S	DW591963			-2.67	Cd	(P25007) Peptidyl-prolyl cis-trans isomerase (EC 5.2.1.8)
tes_tsr_03A07_gal_sal_std_5p_11C	BM413900			-3.28	Cd	(Q7ZYS1) 60S ribosomal protein L19
spl_sts_17H08_sti_sal_std_5p_11C	AJ425625	-3.58		-4.97	ACd	(P63326) 40S ribosomal protein S10
int_oss_T6A03_osl_sal_std_5p_11C	CK884084	-9.32		-8.66	ACd	rRNA promoter binding protein
						
**Cell structure**						
int_oss_T4C19_osl_sal_std_5p_11C	CK885060			-2.15	Cd	collagen a3(I)
int_oss_THA07_osl_sal_std_5p_11C	CN181319			-2.13	Cd	Oncorhynchus mykiss COL1A3 mRNA for collagen a3(I) complete cds
int_rpk_78C22_osl_sgp_std_5p_11C	CK885437			-2.76	Cd	(O00423) Echinoderm microtubule-associated protein-like 1 (EMAP-1)
mus_snm_03C07_sti_tra_nrc_5p_11C	EG649361			-2.76	Cd	(O93484) Collagen alpha 2(I) chain precursor
ova_oyr_04F11_gal_sal_std_5p_11S	BM414013			-3.96	Cd	(P14105) Myosin-9 (Myosin heavy chain nonmuscle IIa)

List of selected mRNAs found up and down regulated resulting from starvation and grouped according to functional classes (shown in bold). The selection was based on manual assignment of function and genes with greatest fold difference in expression are presented, the genes that are down regulated are denoted by (-) value. In this table the genes within each group are ordered by expression level following starvation (PSL). If the genes were significantly altered in the infection these values are also given. The genes shown were significant at P < 0.001 following correction for multiple tests and greater than 2 fold change. ^1^Indicates the unique code for the feature on the microarray, ^2^Accession number of the cDNA sequence, if "No Acc" the TRAITS web page. ^3^Fold change for genes increased in expression following *Aeromonas salmonicida *infection in fish fed a normal diet (AFLvsPFL). ^4^Fold change for genes increased in expression following A. salmonicida infection in fish starved prior to infection (ASLvsPSL). ^5^Fold change for genes increased expression following 28 days starvation. ^6^Identity of the cDNA as determined by BlastX and BlastN searches. ^7^This indicates if this gene is up regulated in one or more other experiments, Ad (AFL), Bd (ASL), Cd (PSL), ABd (AFL+ASL), ABCd (AFL+ASL+PSL). The complete list of genes can be found in Additional file Table S1.

**Table 2:** GO Biological processes significantly altered following starvation

GO Identifier^1^	Term^2^	q^3^	m^4^	p^5^
**Blood and oxygen transport**				
GO:0015669	gas transport	10	19	0.00
GO:0015671	oxygen transport	10	19	0.00
				
**Amino acid metabolism**				
GO:0006544	glycine metabolic process	5	33	0.05
GO:0006541	glutamine metabolic process	3	16	0.08
				
**Fatty acid and steroid metabolism**				
GO:0006720	isoprenoid metabolic process	5	33	0.05
GO:0016125	sterol metabolic process	4	18	0.03
GO:0008299	isoprenoid biosynthetic process	4	10	0.00
GO:0008203	cholesterol metabolic process	3	15	0.06
				
**Immune function**				
GO:0006953	acute-phase response	3	17	0.09
GO:0009617	response to bacterium	3	13	0.05
GO:0042742	defense response to bacterium	3	13	0.05
GO:0006935	chemotaxis	7	27	0.00
				
**Cellular homeostasis**				
GO:0006873	cellular ion homeostasis	7	60	0.06
GO:0030003	cellular cation homeostasis	7	50	0.03
GO:0048878	chemical homeostasis	7	69	0.09
GO:0050801	ion homeostasis	7	61	0.06
				
**Cellular responses**				
GO:0042221	response to chemical stimulus	17	184	0.01
GO:0009605	response to external stimulus	10	56	0.00
GO:0040011	locomotion	8	53	0.01
GO:0007610	behaviour	7	37	0.00
				
**Other processes**				
GO:0022603	regulation of anatomical structure morphogenesis	4	27	0.08
GO:0001501	skeletal system development	4	22	0.05
GO:0018904	organic ether metabolic process	3	8	0.01
GO:0030334	regulation of cell migration	3	17	0.09

Gene ontology (GO) identifiers over represented in genes found differentially expressed following infection. In total 175 genes (69 up regulated and 106 down regulated) genes were included in the list for GO analysis. ^1^Gene ontology identifier, ^2^Gene Ontology term, ^3^number of times the GO term is present in the gene list, ^4^number of times the GO term is present on the filtered array list, ^5^statistical enrichment for GO term. Only GO terms that appear 4 or more times are shown. Full list is shown in Additional file Table S2.

#### *Transcription*, *protein and amino acid metabolism*

Following starvation there was a general decrease in expression of genes related to mRNA transcription, protein synthesis and protein degradation suggesting a reduction in protein metabolism. Genes decreased and related to protein synthesis included ribosomal protein mRNAs, rRNA binding proteins and several key transcription initiation factors. For protein degradation, genes related to the ubiquitin proteasome pathway such as polyubiquitin and proteasome subunits were reduced as was the lysosomal enzyme, cathepsin D. However a calcium activated protease, calpain 1 mRNA was increased following the starvation indicating these pathways of protein degradation are independently controlled. Although a general decrease in genes relating to protein turnover was observed in starved animals, an increase in genes encoding proteins related to amino acid catabolism was found after starvation. The increased amino acid catabolism may generate molecules that contribute to gluconeogenesis and subsequent entry into the TCA cycle.

#### *Lipid and steroid metabolism*

Genes encoding proteins performing steroid biosynthesis and metabolism of fatty acids were up regulated following starvation suggesting a reallocation of lipid reserves and cholesterol metabolism. A number of genes encoding key proteins involved in cholesterol metabolism were found increased including hydroxymethyl-glutaryl-CoA synthetase (HMG-CoA), a rate limiting enzyme in cholesterol biosynthesis, isopentenylpyrophosphate delta-isomerase (IPP), lathosterol, squalene monooxygenase and squalene synthetase, which together demonstrate the fish were attempting to compensate for the dramatic reduction in circulating cholesterol levels following starvation. A number of genes involved in fatty acid metabolism were decreased indicating a general decrease in lipid metabolism.

#### *Serum and oxygen transport*

Plasma and blood transport protein encoding genes were reduced, both α and β haemoglobins, serotransferrin and the calcium binding albumin protein parvalbumin decreased in starved animals indicating a reduced requirement for transport of oxygen and ions in the blood, presumably reflecting reduced energy and oxygen demand from peripheral tissues. A member of the apolipoprotein family, described as an antifreeze protein had reduced expression in starved fish. Two genes central to preventing oxidative damage that showed increased expression were glutathione peroxidase and superoxide dismutase, both implicated in the protection from reactive oxygen species that are often produced as a consequence of nitric oxide synthase activity. Thus these genes may have been up regulated to deal with toxic by products of amino acid catabolism.

#### *Immune and defence genes altered by starvation*

Interestingly, genes encoding a number of secreted immune related proteins were decreased in expression following starvation, including serum amyloid A, complement factor B and serotransferrin, suggesting a reduced level of production of these serum secreted proteins which were constitutively expressed at low levels in uninfected animals. During an infection these are major components of the acute phase response. A mRNA encoding precerebellin, a protein also related to the acute phase response was increased in the starved fish, indicating there is a requirement to maintain this plasma protein at higher levels even during starvation conditions. Two liver specific antimicrobial peptides were also down regulated by starvation, hepcidin and LEAP 2, which could reflect a reduced ability of the starved fish to respond to bacterial infection.

### Transcriptome changes following infection

A large diverse group of genes were found to be modulated by the bacterial challenge, with 615 genes increased and 458 decreased with greater than a two fold change. For those genes increased in expression 318 were commonly up regulated in fish fed or starved prior to the infection, whereas 145 and 123 genes were increased uniquely for fish fed prior to infection (AFL) or starved prior to infection (ASL) respectively (Fig. 2). It is of note that there were more than twice as many genes down regulated in ASL with 397 decreased compared to 170 in the AFL group, which may reflect he requirement of the starved fish to further down regulate genes in order to mount an adequate immune response. Key genes found modulated are presented in Additional file 3 Table S3. To determine the major biological processes being altered by the bacterial infection, enrichment for GO identifiers were assigned to 76 significantly enriched biological processes that were altered following the infection (Table 3, Additional file 2 Table S2). The biological processes that were enriched could be grouped to immune related, cellular homeostasis, protein, lipid and energy metabolism and catabolism.

**Table 3:** GO Biological processes significantly altered following infection

GO Identifier^1^	Term^2^	q^3^	m^4^	p^5^
**Immune related**				
GO:0006952	defense response	30	110	0.00
GO:0009611	response to wounding	27	102	0.01
GO:0006954	inflammatory response	25	70	0.00
GO:0002526	acute inflammatory response	19	39	0.00
				
**Protein metabolism**				
GO:0006519	cellular amino acid and derivative metabolic process	64	296	0.00
GO:0009308	amine metabolic process	55	284	0.09
GO:0044106	cellular amine metabolic process	53	256	0.02
GO:0006520	cellular amino acid metabolic process	51	253	0.05
				
**Energy metabolism**				
GO:0019752	carboxylic acid metabolic process	80	372	0.00
GO:0005975	carbohydrate metabolic process	52	244	0.01
GO:0044262	cellular carbohydrate metabolic process	42	193	0.03
GO:0019318	hexose metabolic process	33	138	0.02
				
**Catabolic process**				
GO:0044282	small molecule catabolic process	43	173	0.00
GO:0046164	alcohol catabolic process	23	96	0.09
GO:0046395	carboxylic acid catabolic process	21	82	0.05
GO:0009063	cellular amino acid catabolic process	19	70	0.04
				
**Lipid and steroid metabolism**				
GO:0006720	isoprenoid metabolic process	12	33	0.01
GO:0045444	fat cell differentiation	3	5	0.08
GO:0006775	fat-soluble vitamin metabolic process	4	7	0.04
				
**Other Processes**				
GO:0044281	small molecule metabolic process	137	683	0.00
GO:0043436	oxoacid metabolic process	80	372	0.00
GO:0040011	locomotion	20	53	0.00
GO:0030003	cellular cation homeostasis	15	50	0.04

Gene ontology (GO) identifiers significantly over represented in genes differentially expressed following infection. In total 698 regulated (414 up regulated and 284 down regulated) genes were included in the list for GO analysis. ^1^Gene ontology identifier, ^2^Gene Ontology term, ^3^number of times the GO term is present in the gene list, ^4^number of times the GO term is present on the filtered array list, ^5^statistical enrichment for GO term. Only GO terms that appear 3 or more times are shown. Full list is shown in Additional file Table S2.

#### *Immune related genes*

As predicted, a large number of genes responding to the bacterial infection could be directly related to immune responses, with GO biological process showing acute phase response proteins, inflammatory response and chemotaxis being significantly up regulated. A number of highly induced genes were acute phase response proteins, including serum amyloid A, haptoglobin, complement factors and precerebellin like protein. As part of the immune recognition of pathogens a number of cell surface receptors were also induced such as C type lectin receptors and Toll like receptors which initiate further signalling to coordinate the immune response. Up regulated transcription factors included Jun B, CAAT enhancer binding proteins α and β, interferon regulatory factor 2 and inhibitor of NFκB amongst others. Genes encoding proteins with direct antibacterial activity were also induced; in particular, hepcidin was dramatically up regulated in both ASL and AFL, and lysozyme was also increased but to a lesser level.

#### *Protein and amino acid metabolism*

The dramatic increase in transcriptional and translational activity for the production of acute phase response proteins and other immune related proteins is expected to have major effects on other processes and potentially at the whole animal level. Ribosomal protein encoding genes and protein elongation factors imply an increase in protein synthesis activity, combined with major protein degradation pathways which were also increased including ubiquitin-proteasome, lysosomal (cathepsins) and calpains. Such changes indicate increased protein turnover reflecting the altered protein production in the liver. A number of genes encoding protein chaperones were increased including heat shock proteins 60, 70 and 108, which could be interpreted as a stress response. Several genes related to amino acid catabolism were increased following infection, a number of these being concerned with metabolism of specific amino acids rather that synthesis and turnover of proteins. Genes related to energy metabolism via glycolysis were also down regulated following infection, as well as genes encoding enzymes in gluconeogenic pathways, perhaps reflecting a reduced use of amino acids as an energy source.

#### *Lipid and steroid metabolism*

There was a general decrease in genes related to lipid and steroid metabolism in those fish that were infected, specifically peroxisomal enzymes and a fatty acid desaturase. A nuclear peroxisome proliferator-activated receptor, a critical regulator of the cholesterol and inflammatory response in macrophages, was increased following infection suggesting tight control of cholesterol metabolism in the infected animals. This can explain the increase in steroid biogenesis related genes including isopentenyl pyrophosphate isomerise 1 and steroidogenic acute regulatory factor that were increased following infection, so as to maintain correct cholesterol balance and potential production of prostaglandins. The genes related to transport of lipids, apolipoprotein (Apo) B, Apo A-I and Apo H were decreased in expression following disease in the ASL group, proteins highly abundant in the plasma. Another plasma protein, serum albumin, was also down regulated following infection, although again, only significantly so for the ASL group.

### Starvation and infection interaction

A key feature of this study was to determine if the feeding regime prior to infection had any effect on the change in the liver transcriptome following infection. The difference in the response between the ASL and AFL treatment groups was examined by extracting this specific contrast (ASL vs AFL) from the linear model. This resulted in the identification of 474 genes (198 at greater than 2 fold) that responded differently to infection depending on prior feeding. From this analysis 103 and 53 genes showed a greater expression in AFL and ASL respectively. The results from the linear model gave 4 possible groups (Table 4, Additional file 4 table S4); 1. increased more in AFL than ASL (51 genes), 2. increased more in ASL than AFL (26 genes) 3. decreased more in ASL than in AFL (51 genes), and 4. decreased more in AFL than ASL (27 genes).

**Table 4:** Genes responding differently to infection between fed and starved fish

TRAITS IDENTIFIER^1^	ACC^2^	AFL/PFL^3^	ASL/PSL^4^	Interaction^5^	IDENTITY^6^
		FC A	FC B	FC C	
**Increased more in AFL than ASL**					
liv_dis_D3C06_abe_tra_sub_0p_11N	AM049619	16.06	6.01	2.67	(Q40313) Caffeoyl-CoA O-methyltransferase (EC 2.1.1.104)
eye_opk_20I02_osl_sgp_std_5p_11S	CO471610	7.56	2.97	2.54	(P27797) Calreticulin precursor (CRP55) (Calregulin) (HACBP) (ERp60)
mus_mfo_13B12_fou_sal_nrp_5p_12M	DW591886	5.60	2.36	2.37	(P36871) Phosphoglucomutase-1 (EC 5.4.2.2)
bra_opk_07F18_osl_sgp_std_5p_11S	CK875291	5.41	1.22	4.43	(O35760) Isopentenyl-diphosphate delta-isomerase 1 (EC 5.3.3.2)
spl_opk_16G20_osl_sgp_std_5p_11C	CK893548	5.08	2.31	2.20	(P11941) Lysozyme C II precursor (EC 3.2.1.17)
liv_opk_12J23_osl_sgp_std_5p_11C	CK888142	4.94	1.95	2.54	AF232215 *Salvelinus fontinalis *steroidogenic acute regulatory protein (StAR)
liv_lrr_06F01_gal_sal_std_5p_11C	No Acc	4.62	1.84	2.51	(O88803) Leukocyte cell-derived chemotaxin 2 precursor
spl_opk_16I18_osl_sgp_std_5p_11S	CK893722	3.54	1.77	2.01	OMLYRNA *O.mykiss *mRNA for lysozyme II
liv_stb_J4D07_sti_tra_sub_0p_11C	AM397498	2.65	1.09	2.43	(P08603) Complement factor H precursor (H factor 1)
liv_lrr_04C03_gal_sal_std_5p_11C	BI468056	2.46	*-1.03*	2.54	(P04186) Complement factor B precursor (EC 3.4.21.47)
liv_opk_12E12_osl_sgp_std_5p_11S	CK888813	2.02	*-1.13*	2.29	(P98093) Complement C3-1
					
**Increased more in ASL than AFL**					
kid_aki_07D09_abe_tra_sub_0p_11S	AM042502	52.18	124.66	-2.39	ONHMHB2M *Oncorhynchus mykiss *beta-2 microglobulin mRNA
kid_aki_05H02_abe_tra_sub_0p_11C	AM042371	46.53	177.59	-3.82	(Q801Y3) Hepcidin 1 precursor
liv_ali_02G12_abe_tra_sub_0p_11C	AM402598	15.77	31.84	-2.02	(P10643) Complement component C7 precursor
kid_aki_04G07_abe_tra_sub_0p_11C	AM042284	13.21	36.72	-2.78	(P81491) Serum amyloid A-5 protein
liv_opk_12G04_osl_sgp_std_5p_11C	CK889070	9.80	32.12	-3.28	(Q9JLF7) Toll-like receptor 5 precursor
liv_dis_D4D03_abe_tra_sub_0p_11N	AM049704	4.72	14.51	-3.07	toll-like leucine-rich repeat protein precursor
liv_opk_12D07_osl_sgp_std_5p_11C	CK888622	3.69	7.44	-2.02	(O15431) High-affinity copper uptake protein 1 (hCTR1) (Copper transporter 1)
liv_dis_D4A07_abe_tra_sub_0p_11N	AM049678	3.09	7.36	-2.38	C type lectin receptor A
liv_ali_04B05_abe_tra_sub_0p_11C	AM402715	1.22	3.08	-2.51	(P80429) Serotransferrin II precursor
ova_oyr_05A10_gal_sal_std_5p_11C	BM414000	1.19	3.24	-2.73	(P62916) Transcription initiation factor IIB (General transcription factor TFIIB)
bra_snb_06F02_osl_tra_nrc_5p_11C	EG647964	1.12	3.21	-2.87	(Q4AEH7) Glutathione peroxidase 2 (EC 1.11.1.9)
					
**Decreased more in ASL than AFL**					
liv_opk_12M05_osl_sgp_std_5p_11C	CK888589	-2.46	-6.49	2.64	(P31029) Serine--pyruvate aminotransferase mitochondrial precursor (EC 2.6.1.51)
liv_opk_12L05_osl_sgp_std_5p_11C	CK888371	-2.47	-5.16	2.09	(P30613) Pyruvate kinase isozymes R/L (EC 2.7.1.40)
liv_opk_12I06_osl_sgp_std_5p_11S	CK889439	-2.68	-7.71	2.88	(P04694) Tyrosine aminotransferase (EC 2.6.1.5)
tes_opk_15A07_osl_sgp_std_5p_11S	CK897836	-2.79	-7.95	2.85	(Q93088) Betaine--homocysteine S-methyltransferase (EC 2.1.1.5)
ova_oyr_04D02_gal_sal_std_5p_11S	BM414075	-4.93	-23.18	4.71	(Q8BTW8) CDK5 regulatory subunit associated protein 1
					
**Decreased more in AFL than ASL**					
liv_lrr_01C06_gal_sal_std_5p_11C	No Acc	-2.09	1.40	-2.93	(P04186) Complement factor B precursor (EC 3.4.21.47)
tes_tsr_02G05_gal_sal_std_5p_11C	BM414288	-2.10	1.44	-3.03	(O00750) Phosphatidylinositol-4-phosphate 3-kinase (EC 2.7.1.154)
mus_amu_05D01_abe_tra_sub_0p_11C	AM412040	-2.31	*-1.05*	-2.19	CHK1 checkpoint homolog (S. pombe)
gil_oss_G6N16_osl_sal_std_5p_11C	CK877483	-2.31	1.40	-3.23	nuclear factor kappa-B 1
spl_sts_20D01_sti_sal_std_5p_11C	AJ425823	-2.54	*1.09*	-2.77	rRNA promoter binding protein
spl_sts_17H08_sti_sal_std_5p_11C	AJ425625	-3.60	*-1.07*	-3.37	(P63326) 40S ribosomal protein S10
ova_opk_10D15_osl_sgp_std_5p_11C	CK891273	-5.23	*-1.14*	-4.60	(Q18268) Phosphodiesterase delta-like protein
int_oss_T6A03_osl_sal_std_5p_11C	CK884084	-9.32	*-1.27*	-7.36	rRNA promoter binding protein

List of mRNAs found to respond to the bacterial infection in a differential manner depending on prior feeding regime. The genes were differentially expressed with P < 0.001 following correction for multiple tests. Only those with a two fold difference in response are shown. ^1^Indicates the unique identifier for the microarray clone on the microarray, ^2^Accession number of the sequence deposited to GenBank, when "No Acc" the sequence can be obtained from the TRAITS web page as described in materials. ^3^Fold change in gene expression between diseased fed and uninfected fed (numbers in italics indicate they are non significant at P < 0.001). ^4^Fold change in gene expression between diseased starved and uninfected starved (numbers in italics indicate they are non significant at P < 0.001). ^5^The fold difference in the response to the disease challenge as result of the prior feeding regime. ^6^Identity of the cDNAs following BlastX or BlastN. Full list shown in Additional file 2 Table S3.

For GO analysis to examine the interaction between infection and feeding regime, both those genes that responded at a greater or decreased level were combined. Fourty eight GO biological processes were significantly altered ie. had a different magnitude in infected fish depending on if they were fed or starved before the infection (Table 5) and included genes involved in immune function, protein metabolism, lipid metabolism and cellular homeostasis.

**Table 5:** GO Biological processes significantly altered in infected fish depending on prior feeding regime

GO Identifier^1^	Term^2^	q^3^	m^4^	p^5^
**Immune related**				
GO:0002376	immune system process	9	135	0.08
GO:0009605	response to external stimulus	9	137	0.08
GO:0006952	defense response	8	110	0.08
GO:0006954	inflammatory response	7	70	0.03
GO:0002253	activation of immune response	4	25	0.03
GO:0006956	complement activation	4	22	0.02
GO:0002526	acute inflammatory response	6	39	0.01
GO:0048583	regulation of response to stimulus	5	52	0.08
GO:0002541	activation of plasma proteins involved in acute inflammatory response	4	22	0.02
GO:0006959	humoral immune response	4	28	0.04
GO:0002684	positive regulation of immune system process	4	30	0.04
GO:0048584	positive regulation of response to stimulus	4	29	0.04
GO:0050776	regulation of immune response	4	35	0.08
GO:0050778	positive regulation of immune response	4	26	0.04
				
**Protein metabolism**				
GO:0016485	protein processing	4	31	0.05
GO:0051604	protein maturation	4	32	0.06
GO:0051605	protein processing by peptide bond cleavage	4	29	0.04
GO:0032880	regulation of protein localization	3	12	0.02
GO:0051222	positive regulation of protein transport	3	12	0.02
GO:0070201	regulation of establishment of protein localization	3	12	0.02
GO:0051223	regulation of protein transport	3	12	0.02
GO:0060341	regulation of cellular localization	3	20	0.07
GO:0045786	negative regulation of cell cycle	3	22	0.08
				
**Lipid and glycerol metabolism**				
GO:0045834	positive regulation of lipid metabolic process	3	20	0.07
GO:0006638	neutral lipid metabolic process	3	8	0.00
GO:0006639	acylglycerol metabolic process	3	8	0.00
GO:0006641	triglyceride metabolic process	3	7	0.00
GO:0006662	glycerol ether metabolic process	3	8	0.00
GO:0018904	organic ether metabolic process	3	8	0.00
				
**Cellular homeostasis**				
GO:0048518	positive regulation of biological process	13	242	0.10
GO:0032879	regulation of localization	7	53	0.01
GO:0051239	regulation of multicellular organismal process	6	63	0.05
GO:0050793	regulation of developmental process	6	41	0.01
GO:0022603	regulation of anatomical structure morphogenesis	6	27	0.00
GO:0051094	positive regulation of developmental process	5	19	0.00
GO:0045766	positive regulation of angiogenesis	5	14	0.00
GO:0045765	regulation of angiogenesis	5	19	0.00
GO:0051049	regulation of transport	4	33	0.07
GO:0051272	positive regulation of cellular component movement	3	11	0.01
GO:0051050	positive regulation of transport	3	16	0.04
GO:0030334	regulation of cell migration	3	17	0.04
GO:0030335	positive regulation of cell migration	3	9	0.01
GO:0040012	regulation of locomotion	3	17	0.04
GO:0040017	positive regulation of locomotion	3	9	0.01
GO:0051270	regulation of cellular component movement	3	20	0.07

Gene ontology (GO) identifiers over represented in genes found differentially expressed following infection depending on prior feeding regime. For this analysis both increased and decreased genes were included. ^1^Gene ontology identifier, ^2^Gene Ontology term, ^3^number of times the GO term is present in the gene list, ^4^number of times the GO term is present on the filtered array list, ^5^statistical enrichment for GO term. Only GO terms that appear 3 or more times are shown.

Genes encoding proteins that are secreted into plasma were affected to the greatest extent between ASL and AFL. Complement components H, B and C1 were more induced in AFL whereas complement C7 showed greater up regulation in ASL. A number of major acute phase protein genes, serum amyloid A (SAA), serotransferrin and β 2 microglobulin (β 2 M) were all highly up regulated to a significantly greater magnitude than in the AFL group, suggesting a greater increase in production of these proteins. Hepcidin also had a 3.8 fold greater increase in ASL than AFL showing again that the ASL group responded differently to the AFL group. It is also of note that both SAA, hepcidin and serotransferrin were decreased in expression in the starved group (PSL). This additional increase in the ASL group compared to AFL may be a compensatory increase due to the decreased expression of these genes in the starved fish. Cell surface receptors including Toll like receptor 5 was up regulated to a greater extent in ASL than AFL, whereas there was a less clear picture with the C type lectins. Thus, maltose binding lectin was down regulted in ASL to a greater degree than in AFL whereas a C type lectin receptor A was more highly up regulated in ASL than AFL.

Genes in the steroid biosynthesis/cholesterol pathway which includes IIP isomerase, steroidogenic acute regulatory protein and lathersterol oxidase were up regulated to a greater extent in the AFL. Expression of genes related to amino acid metabolism were reduced in both ASL and AFL, but a greater reduction was seen in serine pyruvate amino transferase and tyrosine amino transferase where there was a significantly greater reduction in ASL fish.

### Confirmation of expression by real time PCR

Real time PCR analysis was performed on a number of genes for confirmation of microarray data (Table 6). All the genes were those that showed a significant increase in expression following the infection challenges, and represented genes related to different aspects of the immune response.

**Table 6:** Real time PCR analysis of genes selected for confirmation of microarray data.

Group:	SAA	TRL 5	Hepcidin	JunB	CAAT/EBP	COUP	C type lectin	Precerebellin
AFL vs PFL	31.7 ± 4.4*	17.2 ± 4.6*	100.0 ± 20.7*	18.1 ± 3.3*	2.5 ± 0.4	3.0 ± 1.5	3.6 ± 0.6*	15.9 ± 7.5*
ASL vs PSL	128.2 ± 43.8	60.5 ± 26.8*0	424.5 ± 66.2*	8.7 ± 1.4*	5.6 ± 3.0	0.9 ± 0.5*	4.3 ± 2.0*	27.1 ± 13.6*
PSL vs PFL	-6.6 ± 3.7	-1.0 ± 0.3	-1.5 ± 0.4	-0.6 ± 0.3	-1.0 ± 0.6	-13.4 ± 7.7	-1.1 ± 0.2	-0.5 ± 0.2*

Candidate gene expression in liver RNA isolated from Atlantic salmon fed or starved prior to bacterial infection. Values are fold increase (± sem). The groups are: AFL fish that were fed on normal diet prior to bacterial infection compared to fed uninfected fish, PFL. ASL: fish that were starved for 28 days prior to infection compared to uninfected starved fish PSL. PSL uninfected starved fish compared to uninfected fed fish, PFL. Gene expression was determined by real time PCR and normalized to the elongation factor 1α gene. Statistical analysis of expression data was by t-test, * indicates P < 0.05.

These were SAA, Toll like receptor 5, hepcidin, JunB, CAAT, COUP, C type lectin and precerebellin. The qPCR expression was normalized using elongation factor 1 α as this was not found to be modulated in expression by microarray analysis. For all genes the expression pattern showed the same direction of response between microarray and real time PCR analysis, however the magnitude of the expression differences varied between the microarray and qPCR. In general greater differences were observed from real time expression data than from microarray data. For example, a 47 fold increase was found for hepcidin AFL in samples by microarray analysis but ~100 fold difference was found when these samples were examined by qPCR. Similar differences were also found for SAA. The reason for the difference is likely to be the different platforms being used and different protocols for cDNA synthesis and equipment.

## Discussion

Both infection and food withdrawal have major consequences to whole animal physiology with alterations occurring in many cellular processes across numerous tissues. Central to metabolism is the liver, which carries out many processes related to energy metabolism, synthesis and secretion of serum proteins and immune responses. During infection the liver has a number of essential roles, in particular the production of acute phase response proteins (APR) [33] as well as specialized liver specific macrophages, the Kupffler cells [34]. Both starvation and infection have been examined in fish, but rarely has the interaction of these two processes been investigated. A considerable amount of literature is available on the transcriptional response to starvation in different fish species, however care needs to be taken when interpreting the results as many factors including, prior feeding [26], stress [35], energy reserves [36] and sexual maturation status [37] can alter the profile of the response and are impacted by husbandry, developmental and environmental parameters.

Our results showed that both starvation and infection lead to large transcriptional responses. We used 12 microarray slides per experimental group which allowed robust statistical analysis to be performed [38]. All data presented was highly significant at P < 0.001 following correction for multiple tests. Additionally we only considered genes that had a greater than 2 fold difference in expression to help interpret the results. Following starvation, GO analysis showed a number of biological processes to be significantly altered. On examination, genes encoding proteins involved in protein and amino acid metabolism, fatty acid and steroid metabolism, immune function and general energy metabolism were both increased and decreased, whereas genes relating to blood and ion transport, lipid metabolism and energy metabolism were down regulated following starvation.

The liver is a tissue with very high and variable rates of protein synthesis and degradation [39,40]. During starvation the liver receives reduced levels of free amino acids from digestion, and a multitude of hormonal signals mainly from growth hormone and insulin like growth factor [41] which when reduced result in decreased protein synthesis rates. The decrease in protein synthesis rates begins with reduced transcription as seen by the reduction in transcriptional machinery including ribosomal RNA binding proteins and transcription initiation factors. In mammals [19,20] and fish [30] protein synthesis is depressed following reduction in protein intake which appears to be a conserved response. Although both synthesis and degradation are decreased during starvation, the balance between synthesis and degradation shifts towards greater relative degradation [25,42,43], and in turn the reduced protein synthesis levels are reflected in the lower quantities of serum proteins synthesised and secreted [44]. Protein degradation, similar to protein synthesis is a tightly controlled process [45], with two key pathways of protein degradation down regulated in the starved fish, namely genes relating to the ubiquitin proteasome pathway of degradation [46] and lysosomal pathway enzymes including cathepsin D. Although the ubiquitin proteasome pathway in mammals is most often viewed as important in muscle tissue [47] clearly the general decreased transcription of genes in liver in the present study show it is an important pathway in liver tissue in fish. A gene encoding the calcium dependant cysteine protease, calpain-1 was found increased in the starved fish, which demonstrates proteolytic pathways are independently regulated with each pathway targeting different proteins for destruction.

Both protein synthesis and degradation are energy demanding processes and it is highly likely reduced protein turnover acts as an energy conserving mechanism. Following infection there was a rise in genes relating to protein turnover, with those genes relating to both protein synthesis and degradation being up regulated in both the AFL and ASL groups. This would lead to increased demand for ATP production and also in the case of starved animals an increased supply of free amino acids as substrates for protein synthesis. It would be expected that this would have a greater energetic impact on the starved animals which already have reduced energy reserves. Catabolism of amino acids was found increased in starved fish which could suggest further oxidation of amino acids leading to gluconeogenesis and subsequent oxidation [48]. In the diseased fish there was a marked decrease in genes related to amino acid catabolism which could be explained by amino acids being used for increased protein synthesis, however amino acid modification is increased by amino acid transferases and amino acid methylases which may be involved in amino acid biosynthesis. Control of amino acid biosynthesis and oxidation can be dramatically altered by food deprivation [49] and infection [50] in mammals, with redistribution of amino acids, particularly those required for synthesis of inflammatory response proteins (such as APR proteins) which have a different amino acid composition to proteins synthesized during normal growth and metabolism.

During normal growth the liver is a major producer of plasma proteins including serum albumin [51], apolipoproteins [52] and other blood associated proteins which represent a considerable part of the transcription and translation occurring in the liver. During starvation there was a marked decrease in the transcription of these proteins. Both α and β globins were down regulated in the starved animals, reflecting the presumed decrease in whole animal metabolism and decreased oxygen demand. They were also further decreased when the starved animals were immune challenged (ASL group), but the globins were not decreased in the fish which were fed before infection (AFL group). Serum albumin was depressed in starved fish (1.7 fold, data not shown) as also observed in mammals fed low protein diets [53]. The modest decrease in serum albumin in starved fish showed a further decrease in the ASL group which could indicate decreases in total circulating protein. The decrease in circulating globins and serum albumin was also observed in brook trout following bacterial infection [54] and ascribed to a reduction in transcription and potentially additional catabolism induced by the inflammation. In mammals reduced circulating serum albumin levels correlates with increased susceptibility to infection [55] which may also be the case in fish. In parallel to serum albumin, apoliproteins, the major lipid and cholesterol transporters were reduced following infection. It is likely this was brought about by either reduction in lipid metabolism and transport or to a down regulation of these genes to make way for the synthesis of APR proteins.

The greatest increase in transcription in both AFL and ASL groups was for genes related to the APR as has been widely reported in a number of previous studies in liver [4,5,28,29,56]. There was a trend for there to be a greater increase in the ASL as seen for SAA, serotransferrin, β 2 microglobulin and a major antimicrobial peptide (hepcidin) which is often increased in parallel with the APR following bacterial challenge [57]. Interestingly, SAA, serotransferrin and hepcidin were all decreased in the starved fish suggesting the starved fish were down regulating their constitutively expressed immune molecules, most likely as an energy conserving mechanism. Upon infection the fish needed to dramatically increase production of the APR proteins and appear to sacrifice the transcription of normal plasma proteins as seen for serum albumin, globins and apoliproteins. These dramatic changes were likely to have more major consequences for the physiology of the fish in the ASL group than the AFL group. One further APR protein of note is precerebellin [58]. The mRNA for this gene was increased following starvation in uninfected fish, suggesting that there is a requirement for this protein to be maintained at a higher constitutive level, although to date the exact function of the protein remains unknown. The APR is predominantly stimulated by cytokines, in particular IL-6 which acts via its receptor gp130/IL-6R to transduce the signal and stimulate STAT transcription factors to target responsive genes. A number of transcription factors were increased in expression following the infection, including CAAT/enhancer binding protein (also termed C/EBP or nuclear factor IL-6) which binds to promoters of APR proteins and IL-6 target genes [59]. Although c/EBP increases APR transcription it also binds to the promoter of serum albumin decreasing its expression [60]. Toll like receptors (Toll like 5 and a Toll like leucine rich repeat protein) which are related to IL-1 family receptors, signal via NFκB and are key in eliciting an immune response [61], were increased in ASL and AFL. As with several APR proteins, the increase in these cell surface receptors was greater in ASL for both genes which could help explain the magnitude of expression of the APR genes. A further inflammatory regulatory gene, suppressor of cytokine signalling 1 (SOCS1) [62], was decreased in the ASL group. SOCS1 functions to attenuate the inflammatory response to cytokines, hence the decrease in ASL may be a further indication that these fish do not negatively regulate their inflammatory response to the same degree as in fed fish.

The effects of malnutrition on the APR in rats showed that animals on low protein diets had higher levels of APR transcripts in the liver [63] which was interpreted as a low level inflammatory response and hence a preservation of the APR during malnutrition. However other studies suggest an attenuated APR and cytokine response in humans which can lead to increased morbidity following infection [64]. Protein malnourishment in mammals can activate NFkB, leading to inflammation, however our results in fish do not find this to be the case, and in fact the transcription factors controlling these pathways were down regulated, with little evidence for increased transcription of inflammatory response genes in the starved fish.

Despite the depletion of protein reserves the starved fish were still able to mount an APR, or at least induce expression of the genes. Our results suggest the ASL potentially had a greater magnitude of APR than the AFL group, but the APR alone is no indicator of the outcome of an infection.

The removal of free radicals and toxins are achieved in part by antioxidants and we observed a number of antioxidant encoding genes being both increased and decreased during infection. In mammals that were on a restricted calorie intake a number of different isoforms of glutathione peroxidase were both increased and decreased [20]. In fish we have observed similar results with super oxide dismutase and glutathione peroxidase 3 increased in starved fish whereas glutathione peroxidase 2 was decreased following starvation. This may indicate these genes are controlled differently with glutathione peroxidase 3 perhaps having a greater role in detoxifying products of amino acid catabolism. Following infection glutathione peroxidase 2 was increased in both ASL and AFL groups which would indicate a requirement to remove reactive products of increased protein turnover and oxidation [65]. Several different glutathione-s-transferases were found reduced in expression in the ASL fish. Interestingly glutathione-s-transferase activity was down regulated in rainbow trout following 6 weeks starvation [66], a considerably longer time of starvation than in the present study indicating the decrease in the activity of this enzyme may be accelerated by infection.

The complement system which has major roles in innate and acquired immune defences has components differentially expressed following infection in both the ASL and AFL groups of fish. Activation of the complement system during innate immune responses is via the "alternative pathway" or "lectin pathway" [67], with pathogen recognition resulting in increased transcription of complement components. Once activated the complement factors help neutralise pathogens by lysis and phagocytosis, and also act as inducers of inflammation [68]. Complement factors C3-1, C7, B, H and C1q were all increased which is an expected response to acute bacterial infection. However a number of complement factors were found to be down regulated after infection in the ASL group. Components C4 and C1q were both decreased in expression in the ASL group, and both are major factors of the classical complement pathway [69] involved in the binding of immunoglobulin molecules to pathogens during an adaptive immune response. As the major response in this study is an innate response it is possible that this part of the pathway is being attenuated, particularly in the ASL group that has less energy reserves. However C6 was found down regulated in AFL, which is surprising as it is directly involved in the pathogen membrane attack complex and several complement B genes were found increased and decreased following infection. The differential expression of complement factors can be anticipated, as shown in trout infected with *Listonella anguillarum *where C7 was increased but C3 components down regulated [70] suggesting the control of the complement cascade is complicated with different components being independently regulated.

**Figure 3:**
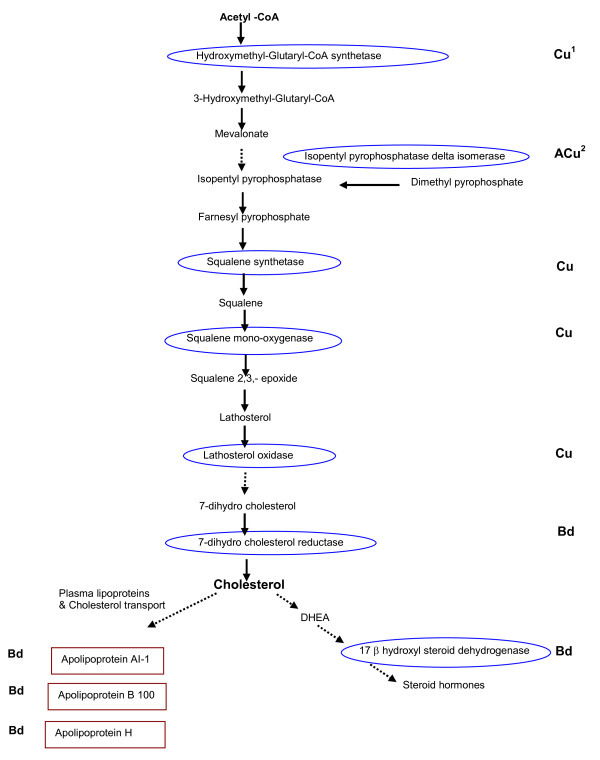
**Genes altered in the cholesterol biosynthesis pathway in starved and infected fish**. Diagram shows key pathway of cholesterol metabolism. Enzymes circled in blue were found to be differentially expressed during microarray analysis. Those proteins boxed in red are also found altered in expression. ^1^Cu indicate up regulated in PSL relative to PFL, ^2^ACu indicates up regulated in AFL and PSL relative to PFL and, ^3^Bd indicates down regulated in ASL group relative to AFL. Expression levels are shown in supplementary Table S1.

Cholesterol and fatty acid metabolism was altered as a result of both starvation and infection. In starvation, several key rate limiting genes involved in the cholesterol biosynthesis pathway were significantly up regulated (Fig. 3). This would indicate a major increase in cholesterol biosynthesis, suggesting the fish are attempting to maintain circulating levels of cholesterol. In fasted cod cholesterol in high density lipoprotein (HDL) increased in serum whereas there was a decrease in low density lipoprotein (LDL) [71]. Interestingly in the cholesterol pathway isopentyl pyrophosphase delta isomerase was increased in the AFL group, as was a steroidogenic acute regulatory factor. However, one of the final enzymes in the production of cholesterol, 7 dehydrocholesterol reductase was down regulated in the ASL fish indicating major differences in cholesterol metabolism between ASL and AFL groups, which was further supported by the reduction of a number of apolipoproteins (Fig. 3) in ASL, that are responsible for lipid and cholesterol transport. The peroxisome proliferator activated receptors (PPARs) are nuclear hormone receptors that act as transcription factors and are critical regulators of lipid and energy homeostasis [72], several of which have recently been described in fish [73]. In our experiments PPARα was increased following infection in both AFL and ASL, indicating increased lipid metabolism within the peroxisome. However two enzymes which have activity within the peroxisome were down regulated in the ASL fish, a trans enoyl CoA isomerise and peroxisomal bifunctional enzyme, both involved in fatty acid biosynthesis and metabolism. Potentially the observed changes in lipid metabolism and peroxisomal activity are related to production of eicosanoids, which have a major role in the control of inflammation [74]. Although the precise mechanisms for the reduction in immune gene expression and other biological pathways following starvation are not known, it is likely these are attributable to hormonal factors including glucocorticoids which are altered following starvation [75] and also have major impacts on the immune system [76].

## Conclusions

Our transcriptome analysis has demonstrated that starvation in salmon has profound effects on biological processes in the liver, with protein, lipid and steroid metabolism being affected. Equally the acute bacterial infection results in a massive increase in APR proteins, and has impacts on protein turnover and cholesterol metabolism. The reallocation of and control of amino acids from normal physiological functions to production of APR proteins is probably the greatest effect we have observed. The starved fish have generally decreased transcription of immune genes but following infection they attempt to recover this, although they have less circulating plasma proteins and hence may have more difficulty in achieving this and potentially expend considerably more protein and energy reserves attempting to deal with the pathogen. This experiment has used an extreme model (starvation and infection) and only examines the mRNA responses for those genes present on the microarray. Future experiments could incorporate additional physiological and immunological parameters. The results presented here may help predict how fish will respond to infection or vaccination on specific diets and the importance of high protein feeds during times of a potential immune challenge.

## Methods

### Fish maintenance and challenge

Juvenile mixed sex Atlantic salmon were maintained in 250 L freshwater tanks at the University of Aberdeen, UK, fish facilities. Water was held at a constant 12°C, pH 7.60 (± 0.05) and 90% (± 1%) of oxygen saturation. The fish were individually marked on the ventral surface by alcian blue dye, which enabled recognition of individuals. Initially the fish were fed *ad libitum *(Nutreco feed). At the beginning of the experiment fish were separated randomly into two tanks, in one tank fish were fed *ad libitum *as before and in the other tank they were not fed for 4 weeks. The initial and final weight and length were recorded and were used to calculate condition factor (K, 100 * BW/FL^-3^) where BW is body weight in g and FL is fork length in mm. Specific growth rate (SGR) was calculated from the equation: SGR = ((LnBW2 - LnBW1)/t) × 100 where BW2 is the final weight, BW1 is the initial weight and t is time between W1 and W2 in days. Following the 4 week trial fish were anaesthetised with bezocaine (Sigma 20 mg l^-1^) and injected with 100 μl of a genetically attenuated (aro A^-^) strain of *Aeromonas salmonicida *(Brivax II[77]) (10^9 ^CFU ml^-1^) in PBS or 100 μl of PBS as control. Fish were returned to separate tanks, either infected or control. The fish were then sacrificed 24 h post challenge and liver tissue was removed immediately and stored in RNAlater (Ambion) at 4°C for 24 h then placed at -20°C until RNA extraction. A total of 96 fish were used in the trial, split into four groups of 24, denoted as AFL (*Aeromonas *infected/fed), ASL (*Aeromonas *infected/starved), PFL (PBS control/fed) and PSL (PBS control/starved).

### RNA isolation

Liver tissue (100 mg) stored in RNAlater was blotted on tissue paper to remove any excess liquid. The tissue was then homogenised in RNA STAT60 (AMS Biotechnology) according to the manufacturer's instructions for purification of total RNA. After precipitation, RNA was resuspended in sterile RNase free water (Sigma). Further RNA purification was performed using RNeasy cleanup columns (Qiagen) according to the manufacturer's protocol. RNA was eluted from these columns in water and the concentration adjusted to 2.5 μg μL^-1^. The integrity of the RNA was determined by electrophoresis (Agilent Bioanalyser 2100) and concentration measured by spectrometry (ND-1000, NanoDrop Technologies). RNA was stored at -80°C until required.

#### Microarray hybridisation and analysis

For microarray analysis 6 pools of RNA were made, each pool containing RNA from 4 different fish chosen randomly from each group. Concentration and quality was rechecked as described above. RNA was reverse transcribed using a FAIRPLAY II cDNA post labelling kit (Stratagene) according to the manufacturer's instructions and labelled separately with both Cy3 or Cy5 (GE HealthCare; PA23001, PA25001) to allow for a swap protocol. All reverse transcriptions were carried out at the same time on 96 well plates to reduce technical variation. Briefly 20 μg total RNA was reverse transcribed after being primed with oligo dT. Following reverse transcription the RNA template was hydrolysed using 1 M NaOH for 15 min and then neutralised with 1 M HCl. The cDNA was ethanol precipitated overnight. The cDNA pellets were washed in 80% ethanol and air dried before being resuspended in 5 μl 2× coupling buffer. Once the cDNA had fully dissolved (after at least 30 min) 5 μl Cy dye was added to each tube and incubated in the dark for 30 min. (Pre-aliquotted Cy3 and Cy5 dyes were resuspended in 45 μl DMSO prior to being added to the coupling buffer.) To remove unincorporated dye, the labelled cDNA (total volume 10 μl) was passed through a DyeEx 2.0 spin column (Qiagen). Dye incorporation was checked by spectrophotometry (ND-1000) and by electrophoresis of labelled cDNA on a mini-gel and visualisation by microarray scanner (Perkin Elmer ScanArray 5000XL). For hybridisation, 20 μl of labelled sample (10 μl Cy3 and 10 μl Cy5) was added to 85 μl hybridisation buffer (Ambion), 10 μl poly(A) potassium salt (Sigma; 10 mg ml^-1^) and 5 μl ultrapure BSA (Ambion;10 mg ml^-1^).

Hybridizations were performed on a Gene TAC Hyb Station (Genomic solutions) for 16 h at 42°C. Following the hybridisations slides were washed with 1× SSC 10 min at 60°C, 1× SSC +0.2% SDS 10 min at 60°C, 0.1 × SSC + 0.2% SDS 10 min at 42°C. Slides were then rinsed in isopropanol and dried by centrifugation before being scanned. All 48 slides were hybridised at the same time to reduce any technical artefacts that may occur.

The slides were scanned on an Axon 4200A scanner (Axon Instruments) at a resolution of 10 μm and saved as *.TIF files. Initial image analysis was by GenPix (version 5.1, Axon instruments) program. The array images were edited to ensure that the GAL (Gene Associated List) files were correctly orientated and that any abnormal hybridization signals were flagged as bad and the image output saved as *.gpr files. Image output files were imported into the R statistical environment [78] using the BioConductor Limma package [79] for preprocessing and further analysis. Individual spot quality weights were derived based on flags generated by the GenPix software and those spots flagged as bad were assigned a weighting of zero in subsequent analysis. Spot intensities were background corrected using the *normexp *method with an offset of 50 to avoid zero or negative intensity values [80]. Intensity dependent loess normalization was performed to balance any dye bias and print-tip effect within each array [81]. Quantile normalization was performed to stabilize the experimental variances across replicate arrays. Differential expression of individual genes was assessed using linear modelling and empirical Bayes methods [79]. The linear model included the effects of bacterial infection (*Aeromonas *infected and PBS control), feeding regime (feeding and fasting) and the interaction between bacterial infection and feeding regime (equivalent to a two-way analysis of variance). Specific comparisons of treatment groups were made by extracting the appropriate contrasts from the linear model. Comparisons were based on a robust *t *statistics in which the standard errors of the estimated log fold changes were moderated across genes using an empirical Bayes approach [82]. Multiple testing was accounted for by controlling the false discovery rate (FDR) at 5% [83]. Clones were assigned as being differentially expressed if both the FDR adjusted *P *values were less than 0.001 and a greater than two fold change in expression level. Details of the TRAITS/SGP microarray are available at European Bioinformatics Institute (EBI) ArrayExpress platform http://www.ebi.ac.uk/arrayexpress under accession number A-MEXP-664. The raw hybridisations data have been deposited at ArrayExpress under accession number (*E-MEXP-2496*). All experimental procedures for array construction, hybridisation and analysis comply with MIAME guidelines [84].

#### Analysis of gene ontology

Enrichment for specific gene ontology (GO) categories was performed on all those cDNA features that had associated GO identifiers using the GOEAST program [85]. Fisher's exact test was used within the GOEAST program to determine if GO identifiers occurred more often in a group than would appear by chance. For GO analysis only biological process GO identifiers were considered that occurred more than 4 times.

#### Real time PCR

All RNA samples for real time PCR were taken from the same pool of material as used for microarray analysis. RNA (2 μg) was denatured (65°C, 10 min) in the presence of 1 μl oligo dT_17 _primer (500 ng μl^-1^), left at room temperature for 5 min to allow annealing, then kept on ice. cDNA was synthesised using 15 U Bioscript reverse transcriptase (Bioline, UK) in the presence of dNTPs (final concentration 200 μM each), at 42°C for 1 h in a final volume of 20 μl. The cDNA was diluted to 100 μl and 3 μl used as template for PCR using primers designed against the Atlantic salmon genes of interest (Table 7). cDNA amplification using ready-prepared 2× SYBR Green PCR master mix (Biorad) was performed in 25 μL volumes on a white BioRad 96 well PCR plate covered with transparent film, and an Opticon qPCR machine was used for monitoring cDNA amplification. A negative control (no template) reaction was also performed for each primer pair. Efficiency of amplification was determined for each primer pair using 10 fold dilutions (1, 10, 100 & 1000 fold dilutions). Elongation factor 1α was used for normalization of expression, previously validated for real time PCR [86]. PCR conditions for all gene assays were 95°C for 5 min followed by 94°C for 15 s, 57°C for 15 s, 72°C for 20 s for a total of 35 cycles. The fluorescence signal output was measured and recorded at 78°C during each cycle for all wells. Melting curves (1°C steps between 75°C - 95°C) ensured that only a single product had been amplified in each reaction. A sample from the serial dilution was separated on an ethidium bromide stained agarose gel, to confirm that a single band of correct size was amplified.

**Table 7:** Primers for real time PCR analysis

Primer name	Gene name	Accession number	Primer sequence	Annealing temp	product size
Exs1F1	Hepcidin	AF542965.1	GCTGTTCCTTTCTCCGAGGTG	55°C	163
Exs1R1			GACAGCAGTTGCAGCACAAAC		
					
sTRL5F1	TRL 5	AY628755.1	GACCCCGGTGTGGCTGAA	55°C	310
sTRL5R1			TGGCTGATTTGTTTTACGCTGT		
					
Exs5F1	Jun B	CA056715.1	TACTGCACTGTTGGGACAGC	55°C	171
Exs5R1			GTTCAGTATGCCCCGAGTGT		
					
Exst10F1	CAAT/EBP	DW550698	CAGCGGGTGTTAAGATCCAT	55°C	200
Exst10R1			GCAGCAGGAGGATCCAAGTA		
					
Exs22F	COUP TF	CK887094.1	CATCGAAAGCCTGCAGGAGAAATC	55°C	165
Exs22R			CCTACCAAGCGGACGAAGAACAGC		
					
Exs3F1	Precerebellin	CB511158.1	GAGGGAACTGACAGCCAGAG	55°C	246
Exs3R1			TTCCCAACATTGCAAGTGAA		
					
Exs2F1	C-type lectin	CB516930	AATCAGTTTGGCAAGCAGCAGA	55°C	374
Exs2R1			AAGCGATTTGAGATGTTTTAGTG		
					
Exs14F	SAA	BQ037050.1	CCCTGCAGGTGCTAAAGACAT	55°C	186
Exs14R			CCTCGACCACTGGAACCCTGAA		
					
EF1AEF	ELF-1α	AF321836.1	CAAGGATATCCGTCGTGGCA	55°C	327
EF1aER			ACAGCGAAACGACCAAGAGG		

PCR primers for real time PCR analysis to assay gene expression of cDNAs found to be differentially expressed by the diet and infection experiments. Amplified cDNA was monitored by Sybr green (BioRad) incorporation on an Opticon real time PCR machine.

To determine the relative expression level of candidate genes the method described by Paffle [87] was employed using elongation factor 1α as house keeping gene. The efficiency of the PCR reaction for each primer set was measured on the same plate as the experimental samples. The efficiency was calculated as E = 10^(-1/s) ^where s is the slope generated from the serial dilutions, when Log dilution is plotted against ^Δ^CT (threshold cycle number). For all real time PCRs six replicates were performed and statistical analysis performed by t-test.

## Authors' contributions

SAM performed microarray experiments, analysed the data, carried out the real time PCR and wrote the manuscript. AD performed the microarray data analysis. DFH and CJS were involved in the experimental design and drafting of the manuscript. All authors read and approved the final manuscript.

## Supplementary Material

Additional file 1 **Table S1**. Full list of genes altered significantly in following infection and starvation. Click here for file

Additional file 2 **Table S2**. GO Biological processes enriched following starvation and infection. Click here for file

Additional file 3 **Table S3**. Genes altered significantly in liver by infection. Click here for file

Additional file 4 **Table S4**. Full list of genes responding differently to infection between fed and starved fish. Click here for file
