# Supplementary material for: Starvation alters the liver transcriptome of the innate immune response in Atlantic salmon (Salmo salar)
Source: BMC Genomics. 2010 Jul 5;11:418. doi: 10.1186/1471-2164-11-418 (PMC2996946; doi:10.1186/1471-2164-11-418)
Supplement: Additional file 1 — Table S1. Full list of genes altered significantly in following infection and starvation. [file 1471-2164-11-418-S1.PDF]

Additional file 1 Table S1. Full list of genes altered significantly in following infection and starvation.

Up regulated genes

TRAITS IDENTIFIER[1]	ACC[2]	AFL/PFL[3] FC A	ASL/PSL[4] FC B	PSL/ PFL[5] FC C	group [7]	Identity[6]
int_oss_THM13_osl_sal_std_5p_11C	CN181442	12.33			Au	extensin 1 - *Arabidopsis thaliana*
kid_sts_14H05_sti_sal_std_5p_11C	AJ424714	11.35			Au	No Hit
tes_opk_15F13_osl_sgp_std_5p_11S	CK897205	6.43			Au	similar to cytochrome b5 domain-containing protein like (3F409)
spl_opk_16E17_osl_sgp_std_5p_11S	CK894910	6.33			Au	No Hit
spl_sts_13G10_sti_sal_std_5p_11C	AJ425503	4.30			Au	(Q9Y3C1) Protein CGI-117
tes_opk_13B06_osl_sgp_std_5p_11S	CK898289	3.85			Au	No Hit
gil_rpk_76C19_osl_sgp_std_5p_11S	CK878600	3.78			Au	Parahucho perryi DNA microsatellite locus Hper-20
spl_opk_16I18_osl_sgp_std_5p_11S	CK893722	3.57			Au	*O.mykiss* mRNA for lysozyme II
spl_sts_05A01_sti_sal_std_5p_11C	AJ425281	3.53			Au	(O08623) Sequestosome-1 (Ubiquitin-binding protein p62)
swi_rpk_74J14_osl_sgp_std_5p_11C	CK896211	3.52			Au	AF483530_1VHSV-induced protein-4
eye_opk_18H11_osl_sgp_std_5p_11C	CK876700	3.42			Au	IBD1278
liv_dis_D1B08_abe_tra_sub_0p_11N	AM049455	3.24			Au	*Danio rerio* similar to myosin light chain kinase isoform 6 (LOC559667) mRNA
eye_opk_17K11_osl_sgp_std_5p_11S	CK877024	3.24			Au	No Hit
ova_oyr_07G04_gal_sal_std_5p_11C	BM414024	3.17			Au	(Q9YH91) Claudin-like protein ZF-A89 (Claudin d)
eye_opk_19E01_osl_sgp_std_5p_11S	CO470297	3.14			Au	(O15393) Transmembrane protease serine 2 precursor (EC 3.4.21.-)
kid_sts_09A04_sti_sal_std_5p_11S	AJ424409	3.01			Au	No Hit
gil_oss_G5G11_osl_sal_std_5p_11S	CK878874	2.97			Au	(Q64337) Sequestosome-1 (Ubiquitin-binding protein p62) (STONE14)
ova_oyr_06H02_gal_sal_std_5p_11S	No Acc	2.93			Au	(O15320) Cutaneous T-cell lymphoma-associated antigen 5 (cTAGE-5 protein)
kid_opk_01A01_osl_sgp_std_5p_11C	CK887252	2.93			Au	ONU56710Oncorhynchus nerka microsatellite Oneu10 DNA
eye_rpk_73B19_osl_sgp_std_5p_11C	CO472091	2.92			Au	(Q9EPC5) Retinoid-binding protein 7 (Cellular retinoic acid-binding protein 4) (CRBP4)
bra_snb_13F03_osl_tra_nrc_5p_11C	EG648030	2.89			Au	(P08962) CD63 antigen (Melanoma-associated antigen ME491)
gil_oss_G6M22_osl_sal_std_5p_12C	CK877325	2.89			Au	Schistosoma japonicum SJCHGC04786 protein mRNA complete cds
gil_oss_G6M19_osl_sal_std_5p_11C	CK877244	2.86			Au	No Hit
eye_opk_18M07_osl_sgp_std_5p_11C	CO469850	2.86			Au	Danio rerio zgc:55813 mRNA (cDNA clone MGC:77830 IMAGE:7001751)
bra_bfo_08B04_fou_sal_nrc_5p_11C	DW589103	2.80			Au	(Q91V92) ATP-citrate synthase (EC 2.3.3.8) (ATP-citrate (pro-S-)-lyase)
mus_snm_12B04_osl_tra_nrc_5p_11C	EG648946	2.74			Au	similar to heterogeneous nuclear ribonucleoprotein A0 isoform 3
bra_snb_10C11_osl_tra_nrc_5p_11C	EG647741	2.74			Au	Zgc:101724 protein
spl_sts_12F02_sti_sal_std_5p_11C	AJ425392	2.73			Au	No Hit
ova_opk_10P20_osl_sgp_std_5p_11C	CK890006	2.73			Au	Danio rerio zgc:64047 (zgc:64047) mRNA
ova_opk_11N15_osl_sgp_std_5p_11S	CK889641	2.70			Au	No Hit

liv_stb_J4D07_sti_tra_sub_0p_11C	AM397498	2.65	Au	(P08603) Complement factor H precursor (H factor 1)
int_oss_T5E19_osl_sal_std_5p_11C	CK885726	2.65	Au	ADP-ribosylation factor 1
gil_oss_G6A22_osl_sal_std_5p_11S	CK877266	2.60	Au	hypothetical protein XP_701264
ova_opk_12C09_osl_sgp_std_5p_11C	CK889908	2.60	Au	Danio rerio hypothetical protein LOC554574 (LOC554574) mRNA
bra_bfo_06D12_fou_sal_nrc_5p_11C	DW588837	2.57	Au	(P27449) Vacuolar ATP synthase 16 kDa proteolipid subunit (EC 3.6.3.14)
int_oss_T4C16_osl_sal_std_5p_11S	CK884600	2.54	Au	similar to Spectrin beta chain brain 2 (Spectrin non-erythroid beta chain 2)
gil_oss_G5O24_osl_sal_std_5p_11C	CK878985	2.52	Au	No Hit
tes_tsr_03D01_gal_sal_std_5p_11C	No Acc	2.51	Au	(Q15291) Retinoblastoma-binding protein 5 (RBBP-5)
kid_sts_10B05_sti_sal_std_5p_11C	AJ424513	2.51	Au	No Hit
hrt_opk_05C10_osl_sgp_std_5p_11C	CK882772	2.50	Au	Salmo salar BAC S0188I22 partial sequence
spl_opk_16D15_osl_sgp_std_5p_11C	CK894854	2.50	Au	(P84248) Histone H3.3
ova_oyr_08E05_gal_sal_std_5p_11S	BM414527	2.49	Au	unnamed protein product
hrt_opk_04K13_osl_sgp_std_5p_11S	CK883676	2.49	Au	Stat3
kid_opk_01M05_osl_sgp_std_5p_11C	CK887114	2.48	Au	(Q9CZC8) Secernin-1
int_oss_T4B17_osl_sal_std_5p_11C	CK884678	2.47	Au	Tax1 binding protein 1
hkd_opk_03J11_osl_sgp_std_5p_11C	CK880515	2.46	Au	Salmo salar zonadhesin-like gene complete cds and 3' UTR
liv_lrr_04C03_gal_sal_std_5p_11C	BI468056	2.46	Au	(P04186) Complement factor B precursor (EC 3.4.21.47) (C3/C5 convertase)
tes_opk_14M09_osl_sgp_std_5p_11C	CK897632	2.45	Au	CNS0GPM1Tetraodon nigroviridis full-length cDNA
kid_opk_01G01_osl_sgp_std_5p_11S	CK887788	2.45	Au	Salmo salar BAC S0188I22 partial sequence
int_rpk_78L23_osl_sgp_std_5p_11C	CK884315	2.42	Au	hypothetical protein LOC550244
ova_opk_09N24_osl_sgp_std_5p_11C	CK890985	2.41	Au	(Q9UKR5) Probable ergosterol biosynthetic protein 28
bra_snb_03C11_sti_tra_nrc_5p_11S	EG648345	2.40	Au	fimbriae-associated protein Fap1
hkd_opk_02D05_osl_sgp_std_5p_11S	CK880916	2.40	Au	similar to CDNA sequence BC006933
ski_opk_09C19_osl_sgp_std_5p_11S	CK892452	2.38	Au	(Q08851) Syntaxin-5
bra_opk_07E13_osl_sgp_std_5p_11S	CK875228	2.36	Au	(Q01405) Protein transport protein Sec23A (SEC23-related protein A)
gil_agi_03E09_abe_tra_sub_0p_11S	AM041624	2.35	Au	(Q5BJI9) Probable signal peptidase complex subunit 2 (EC 3.4.-.-)
gil_oss_G6E23_osl_sal_std_5p_11C	CK877204	2.35	Au	(P50894) 40S ribosomal protein S7
kid_opk_01C19_osl_sgp_std_5p_11S	CK887587	2.35	Au	synaptonemal complex protein 3
spl_sts_16H10_sti_sal_std_5p_22C	AJ425549	2.35	Au	(Q09251) Hypothetical RING finger protein C16C10.5 in chromosome III
hkd_opk_03H15_osl_sgp_std_5p_11S	CK880355	2.34	Au	No Hit
kid_sts_08G04_sti_sal_std_5p_22C	AJ424386	2.32	Au	(P12815) Programmed cell death protein 6 (Probable calcium-binding protein ALG-2) (PMP41) (ALG-257)
mus_snm_04H07_sti_tra_nrc_5p_11C	EG649487	2.31	Au	(Q13151) Heterogeneous nuclear ribonucleoprotein A0 (hnRNP A0)
spl_sts_17F07_sti_sal_std_5p_11C	AJ425602	2.31	Au	(Q5RF83) Cold-inducible RNA-binding protein (Glycine-rich RNA-binding protein CIRP)
hkd_opk_03O11_osl_sgp_std_5p_11S	CK880753	2.30	Au	No Hit
mus_snm_06F04_osl_tra_nrc_5p_11S	EG649059	2.29	Au	Danio rerio zgc:56317 (zgc:56317) mRNA
liv_dis_D2A01_abe_tra_sub_0p_11N	AM049516	2.29	Au	(Q864W1) Complement factor B precursor (EC 3.4.21.47)
hrt_opk_06L11_osl_sgp_std_5p_11C	CK874863	2.29	Au	(Q95LJ0) Proto-oncogene serine/threonine-protein kinase pim-1 (EC 2.7.1.37)

gil_agi_05G12_abe_tra_sub_0p_11S	AM041801	2.28	Au	chemokine CXC-like protein
hrt_opk_06O03_osl_sgp_std_5p_11C	CK874965	2.28	Au	Danio rerio zgc:56419 (zgc:56419) mRNA
hkd_opk_02O21_osl_sgp_std_5p_11S	CK881620	2.27	Au	Oncorhynchus tshawytscha clone Ots.u202.43.68 genomic sequence
bra_bfo_15H01_fou_sal_nrp_5p_11M	DW590148	2.25	Au	No Hit
mus_snm_10F12_osl_tra_nrc_5p_11S	EG648841	2.24	Au	(Q99624) System N amino acid transporter 1 (SN1) (N-system amino acid transporter 1)
spl_sts_13C04_sti_sal_std_5p_11C	AJ425450	2.24	Au	unknown protein
int_rpk_76O05_osl_sgp_std_5p_11C	CK885327	2.23	Au	(Q5R651) 14-3-3 protein zeta/delta
gil_oss_G6G10_osl_sal_std_5p_11S	CK877291	2.23	Au	Salmo salar BAC S0188I22 partial sequence
bra_snb_06A07_osl_tra_nrc_5p_11C	EG648330	2.23	Au	(Q5R9C7) Microsomal signal peptidase 18 kDa subunit (EC 3.4.-.-) (SPase 18 kDa subunit)
bra_snb_14H01_osl_tra_nrc_5p_11S	EG648141	2.22	Au	No Hit
hkd_opk_02I16_osl_sgp_std_5p_11S	CK881264	2.21	Au	hypothetical protein LOC406794
bra_bfo_09E07_fou_sal_nrc_5p_11M	DW589326	2.21	Au	(P27449) Vacuolar ATP synthase 16 kDa proteolipid subunit (EC 3.6.3.14)
eye_opk_19E02_osl_sgp_std_5p_11C	CO470298	2.20	Au	telomerase binding protein p23
int_rpk_76G19_osl_sgp_std_5p_11S	CK884539	2.20	Au	protein tyrosine phosphatase 4a2
ova_opk_10B04_osl_sgp_std_5p_11C	CK891143	2.19	Au	similar to NTF2-related export protein 2 (p15-2 protein)
kid_sts_01D02_sti_sal_std_5p_11C	AJ424115	2.19	Au	Salmo salar BAC S0188I22 partial sequence
bra_snb_05A04_sti_tra_nrc_5p_11C	EG647378	2.19	Au	(Q15008) 26S proteasome non-ATPase regulatory subunit 6 (26S proteasome regulatory subunit S10)
swi_rpk_74K07_osl_sgp_std_5p_11C	CK896140	2.19	Au	No Hit
kid_sts_14A10_sti_sal_std_5p_11C	AJ424636	2.19	Au	(P03934) Transposable element Tc1 transposase
gil_oss_GHF13_osl_sal_std_5p_11C	CN181066	2.18	Au	No Hit
gil_rpk_75K21_osl_sgp_std_5p_11C	CK877770	2.18	Au	(P56399) Ubiquitin carboxyl-terminal hydrolase 5 (EC 3.1.2.15)
mus_mfo_04C11_fou_sal_nrp_5p_11M	DW590625	2.17	Au	hyperosmotic glycine rich protein
bra_bfo_06F07_fou_sal_nrc_5p_11M	DW588872	2.16	Au	(P22122) RAS-like GTP-binding protein O-RHO
eye_opk_19B13_osl_sgp_std_5p_11S	CO470137	2.16	Au	activating transcription factor 4
bra_bfo_12F02_fou_sal_nrc_5p_11M	DW589815	2.16	Au	(Q37676) NADH-ubiquinone oxidoreductase chain 1 (EC 1.6.5.3) (NADH dehydrogenase subunit 1)
mus_snm_04E04_sti_tra_nrc_5p_11S	EG649453	2.15	Au	(P84082) ADP-ribosylation factor 2
eye_opk_17L11_osl_sgp_std_5p_11S	CK877088	2.14	Au	No Hit
gil_oss_G6M22_osl_sal_std_5p_22C	CK877325	2.14	Au	Schistosoma japonicum SJCHGC04786 protein mRNA complete cds
tes_tsr_03D10_gal_sal_std_5p_11C	BM414295	2.14	Au	(O60784) Target of Myb protein 1
spl_sts_04C01_sti_sal_std_5p_11C	AJ425093	2.14	Au	(P30436) Tubulin alpha chain
gil_agi_04B07_abe_tra_sub_0p_11C	AM041672	2.14	Au	CNS0FOE7Tetraodon nigroviridis full-length cDNA
kid_sts_02A04_sti_sal_std_5p_11C	AJ424170	2.13	Au	cathepsin B preproprotein
int_oss_T5M06_osl_sal_std_5p_22C	CK885851	2.13	Au	cathepsin Y
spl_sts_13F07_sti_sal_std_5p_11S	AJ425489	2.12	Au	Salmo salar clone Hae338 microsatellite sequence
mus_snm_04A11_sti_tra_nrc_5p_11C	EG649406	2.12	Au	Oncorhynchus mykiss BAC 127C24 partial sequence
kid_sts_15C06_sti_sal_std_5p_12C	AJ424749	2.12	Au	chemokine (C-C motif) ligand 13

mus_snm_01F08_sti_tra_nrc_5p_11S	No Acc	2.12	Au	No Hit
tes_opk_14A02_osl_sgp_std_5p_11S	CK898981	2.12	Au	(Q8BK64) Activator of 90 kDa heat shock protein ATPase homolog 1 (AHA1)
bra_snb_08F07_osl_tra_nrc_5p_11S	EG647585	2.11	Au	(Q86UL8) Atrophin-1-interacting protein 1 (Atrophin-1-interacting protein A)
bra_bfo_15G04_fou_sal_nrp_5p_11S	DW590141	2.11	Au	(P51992) Heterogeneous nuclear ribonucleoprotein A3 homolog 2 (hnRNP A3(B))
liv_opk_12E06_osl_sgp_std_5p_11S	CK888785	2.11	Au	No Hit
gil_rpk_75L17_osl_sgp_std_5p_11S	CK878006	2.11	Au	suppressor for yeast mutant
eye_opk_18G10_osl_sgp_std_5p_11S	CK876643	2.10	Au	Salmo salar clone Rsa226 microsatellite sequence
ova_oyr_06H10_gal_sal_std_5p_11S	No Acc	2.10	Au	hypothetical protein LOC422249
liv_lra_02F01_gal_sal_std_5p_11C	No Acc	2.10	Au	similar to eukaryotic translation elongation factor 1 delta isoform b isoform 1
spl_sts_04B08_sti_sal_std_5p_11C	AJ425088	2.10	Au	death associated protein 1b
spl_opk_16L21_osl_sgp_std_5p_11C	CK893984	2.10	Au	(Q9IA79) Probable Bax inhibitor-1 (BI-1)
eye_opk_20I09_osl_sgp_std_5p_11C	CO471627	2.10	Au	Oncorhynchus mykiss mRNA for membrane protein p24B
bra_snb_12A04_osl_tra_nrc_5p_11C	EG647883	2.09	Au	ADP-ribosylation factor 2
mus_mfo_07E04_fou_sal_nrp_5p_11C	DW591076	2.09	Au	No Hit
gil_rpk_75K12_osl_sgp_std_5p_11S	CK877696	2.09	Au	AF180490Salmo salar clone BE7 beta-2 microglobulin (B2m) mRNA complete cds
bra_snb_08D08_osl_tra_nrc_5p_11S	EG647565	2.08	Au	activin A receptor type II-like 1
tes_tsr_06C01_gal_sal_std_5p_11C	No Acc	2.08	Au	CD63
hkd_opk_03O08_osl_sgp_std_5p_11C	CK880744	2.06	Au	*Oncorhynchus mykiss* SYPG1 (SYPG1) PHF1 (PHF1) and RGL2 (RGL2)
kid_aki_05C09_abe_tra_sub_0p_11S	AM042327	2.06	Au	(P39656) Dolichyl-diphosphooligosaccharide--protein glycosyltransferase 48 kDa
gil_oss_G6E22_osl_sal_std_5p_11S	CK877287	2.06	Au	No Hit
hkd_opk_02P24_osl_sgp_std_5p_11C	CK881672	2.05	Au	CCAAT/enhancer binding protein alpha
tes_opk_13J11_osl_sgp_std_5p_11C	CK898699	2.05	Au	Salmo salar clone BAC S0085O16 partial sequence
mus_snm_08D12_osl_tra_nrc_5p_11C	EG648628	2.05	Au	(P68037) Ubiquitin-conjugating enzyme E2 L3 (EC 6.3.2.19)
gil_oss_G6E21_osl_sal_std_5p_11S	CK877203	2.05	Au	Oncorhynchus mykiss genes MHC class I a region complete and partial cds
kid_sts_11A03_sti_sal_std_5p_12C	AJ424589	2.04	Au	beta-2 microglobulin
kid_sts_05C11_sti_sal_std_5p_11C	AJ424283	2.04	Au	Danio rerio similar to Interferon induced with helicase C domain protein 1
ova_opk_09M10_osl_sgp_std_5p_11C	CK890911	2.04	Au	(P21670) Proteasome subunit alpha type 4 (EC 3.4.25.1) (Proteasome component C9)
ova_opk_12A22_osl_sgp_std_5p_11S	CK889819	2.03	Au	SSGNRHS.salar sGnRH gene
kid_opk_01D21_osl_sgp_std_5p_11C	CK887647	2.03	Au	(Q7TP47) Heterogeneous nuclear ribonucleoprotein Q (hnRNP Q) (hnRNP-Q)
ova_oyr_01A11_gal_sal_std_5p_11S	No Acc	2.03	Au	CNS0G0XLTetraodon nigroviridis full-length cDNA
liv_ali_02E08_abe_tra_sub_0p_11C	AM402582	2.03	Au	SURF4_FUGRUSurfeit locus protein 4
ski_opk_08P05_osl_sgp_std_5p_11S	CK892070	2.03	Au	Oncorhynchus mykiss cyp19b-I gene for P450aromB-I exons 1-10
ova_oya_01E08_gal_sal_std_5p_11C	No Acc	2.03	Au	(Q9JI19) Acidic fibroblast growth factor intracellular binding protein
ski_opk_08N12_osl_sgp_std_5p_11S	CK891936	2.03	Au	No Hit
gil_rpk_75M22_osl_sgp_std_5p_11C	CK878109	2.02	Au	heat shock 60 kD protein 1
liv_opk_12E12_osl_sgp_std_5p_11S	CK888813	2.02	Au	(P98093) Complement C3-1 [ContainsComplement C3 beta chain
hrt_opk_07F01_osl_sgp_std_5p_11C	CK875251	2.02	Au	ATPase H+ transporting V1 subunit F

spl_opk_15M24_osl_sgp_std_5p_11C	CK894445	2.01		Au	Oncorhynchus clarki henshawi microsatellite Och9 sequence
gil_oss_G6B17_osl_sal_std_5p_11C	CK877344	2.01		Au	Oncorhynchus mykiss SYPG1 (SYPG1) PHF1 (PHF1) and RGL2 (RGL2)
eye_opk_18C14_osl_sgp_std_5p_11C	CK876413	2.00		Au	Sus scrofa mRNA clone:UTR010008C03 expressed in uterus
hkd_opk_03M11_osl_sgp_std_5p_11C	CK882228	2.00		Au	Oncorhynchus mykiss mineralocorticoid receptor form A gene partial cds
int_oss_T4J13_osl_sal_std_5p_11C	CK884714	2.00		Au	(P62262) 14-3-3 protein epsilon (14-3-3E) (Protein kinase C inhibitor protein 1) (KCIP-1)
hkd_opk_03J02_osl_sgp_std_5p_11C	CK880489		5.95	Bu	Oncorhynchus mykiss interferon regulatory-factor 2 mRNA
hkd_opk_03I22_osl_sgp_std_5p_11S	CK880463		5.69	Bu	Cyclic-AMP-dependent transcription factor ATF-5
spl_opk_16E18_osl_sgp_std_5p_11S	CK894917		4.72	Bu	Salmo salar BAC S0188I22 partial sequence
liv_opk_12L16_osl_sgp_std_5p_11C	CK888465		4.66	Bu	No hit
liv_opk_12I14_osl_sgp_std_5p_11S	CK889507		4.40	Bu	(P80429) Serotransferrin II precursor (Siderophilin II)
ova_oyr_04F11_gal_sal_std_5p_11S	BM414013		4.09	Bu	(P14105) Myosin-9 (Myosin heavy chain nonmuscle IIa)
hkd_opk_03G12_osl_sgp_std_5p_11S	CK880278		3.46	Bu	No Hit
ova_oyr_07B05_gal_sal_std_5p_11C	BM413972		3.31	Bu	(Q9UJZ1) Stomatin-like protein 2 (SLP-2) (EPB72-like 2)
mus_amu_08F10_abe_tra_sub_0p_11C	AM083822		3.29	Bu	Oncorhynchus tshawytscha virus-inducible stress protein (VISP) mRNA
ova_oyr_05A10_gal_sal_std_5p_11C	BM414000		3.24	Bu	(P62916) Transcription initiation factor IIB (General transcription factor TFIIB)
bra_snb_06F02_osl_tra_nrc_5p_11C	EG647964		3.21	Bu	(Q4AEH7) Glutathione peroxidase 2 (EC 1.11.1.9)
liv_ali_05C10_abe_tra_sub_0p_11S	AM402809		3.18	Bu	(Q07949) Probable phosphatase PSR2 (EC 3.1.3.-)
liv_ali_04B05_abe_tra_sub_0p_11C	AM402715		3.08	Bu	(P80429) Serotransferrin II precursor (Siderophilin II) (STF II)
kid_aki_04F05_abe_tra_sub_0p_11C	AM042270		2.99	Bu	(Q9YHC3) Tubulin beta-1 chain (Beta-1 tubulin)
spl_opk_16C09_osl_sgp_std_5p_11C	CK894789		2.95	Bu	interleukin 1 receptor accessory protein
eye_opk_18N17_osl_sgp_std_5p_11S	CO469916		2.94	Bu	(Q92183) Alpha-N-acetylgalactosaminide alpha-2 6-sialyltransferase 1 (EC 2.4.99.3)
bra_bfo_16D09_fou_sal_nrp_5p_11S	DW590167		2.94	Bu	Danio rerio ataxin 2-binding protein 1-like (a2bp1l) mRNA
ova_oyr_04C04_gal_sal_std_5p_11S	BM414422		2.90	Bu	(P15101) Dopamine beta-hydroxylase precursor (EC 1.14.17.1)
mus_snm_05E03_sti_tra_nrc_5p_11C	EG648476		2.89	Bu	(Q5RF02) T-complex protein 1 epsilon subunit (TCP-1-epsilon) (CCT-epsilon)
tes_opk_15C17_osl_sgp_std_5p_11C	CK897954		2.87	Bu	(Q8AXL1) Diamine acetyltransferase 1 (EC 2.3.1.57)
tes_opk_14L09_osl_sgp_std_5p_11C	CK897575		2.84	Bu	(Q8AWW7) RuvB-like 1 (EC 3.6.1.-) (Pontin) (zPontin)
bra_snb_06A08_osl_tra_nrc_5p_11C	EG647317		2.83	Bu	(P68372) Tubulin beta-? chain
spl_sts_13D10_sti_sal_std_5p_11C	AJ425468		2.82	Bu	*Oncorhynchus tshawytscha* insulin-like growth factor I (IGF-I.1) gene
bra_snb_01E01_sti_tra_nrc_5p_11S	No Acc		2.78	Bu	(P06686) Sodium/potassium-transporting ATPase alpha-2 chain precursor (EC 3.6.3.9)
liv_ali_04F09_abe_tra_sub_0p_11S	AM402761		2.78	Bu	adenosine kinase a
hkd_opk_02I06_osl_sgp_std_5p_11C	CK881246		2.77	Bu	Sus scrofa mRNA clone:OVRM10069A02 expressed in ovary
int_rpk_78J06_osl_sgp_std_5p_11S	CK886440		2.77	Bu	hypothetical protein XP_533264
swi_rpk_74G19_osl_sgp_std_5p_11S	CK895563		2.75	Bu	No Hit
eye_opk_17O06_osl_sgp_std_5p_11S	CK876166		2.74	Bu	No Hit
tes_opk_12M22_osl_sgp_std_5p_11S	CK898088		2.72	Bu	CNS0G2P6Tetraodon nigroviridis full-length cDNA
mus_mfo_03F03_fou_sal_nrp_5p_11M	DW590559		2.72	Bu	similar to Methionine aminopeptidase 2 (MetAP 2) (Peptidase M 2)

swi_rpk_74D13_osl_sgp_std_5p_11S	CK896771	2.68	Bu	No Hit
gil_rpk_75L11_osl_sgp_std_5p_11S	CK877857	2.66	Bu	hypothetical protein LOC406633
bra_snb_14H07_osl_tra_nrc_5p_11C	EG648148	2.65	Bu	(P62155) Calmodulin (CaM)
kid_aki_02F11_abe_tra_sub_0p_11C	AM042106	2.64	Bu	Oncorhynchus keta clone Oke.u12.50.19 genomic sequence
tes_opk_13G14_osl_sgp_std_5p_11S	CK898551	2.60	Bu	(Q8TDN6) Brix domain containing protein 2 (Ribosome biogenesis protein Brix)
ova_oyr_02G08_gal_sal_std_5p_11C	No Acc	2.59	Bu	similar to NY-REN-58 antigen
mus_snm_12A09_osl_tra_nrc_5p_11C	EG648941	2.58	Bu	phosphoglycerate kinase
gil_rpk_75I23_osl_sgp_std_5p_11C	CK877738	2.55	Bu	HUCHHP15Hucho perryi DNA for repeat sequence Hpa I
swi_rpk_74H03_osl_sgp_std_5p_11C	CK895420	2.53	Bu	AF338347_1eukaryotic initiation factor 2 alpha subunit
int_oss_T4K19_osl_sal_std_5p_11S	CK885103	2.52	Bu	(P30352) Splicing factor arginine/serine-rich 2 (Splicing factor SC35)
kid_sts_05C01_sti_sal_std_5p_11C	AJ424274	2.51	Bu	hypothetical protein XP_697335
ova_opk_11K17_osl_sgp_std_5p_11C	CK890555	2.50	Bu	(O00488) Zinc finger protein 593 (Zinc finger protein T86)
eye_opk_18D18_osl_sgp_std_5p_11C	CK876492	2.47	Bu	UPF0373 protein precursor
ova_oyr_02D07_gal_sal_std_5p_11C	BM414466	2.47	Bu	sperm acrosomal membrane protein 14
hkd_opk_03A24_osl_sgp_std_5p_11S	CK881754	2.45	Bu	CNS0G7MKTetraodon nigroviridis full-length cDNA
bra_snb_06E08_osl_tra_nrc_5p_11C	EG647910	2.45	Bu	(P79251) Vacuolar ATP synthase subunit G 1 (EC 3.6.3.14) (V-ATPase G subunit 1)
mus_mfo_07F04_fou_sal_nrp_5p_11C	DW591096	2.43	Bu	novel protein similar to vertebrate lumican (LUM)
bra_snb_09C05_osl_tra_nrc_5p_11C	EG647646	2.43	Bu	amyloid precursor-like protein 2
bra_bfo_05E09_fou_sal_nrc_3p_11C	DW588674	2.43	Bu	hypothetical protein LOC550548
int_oss_T5J14_osl_sal_std_5p_11S	CK886001	2.40	Bu	(Q8R059) UDP-glucose 4-epimerase (EC 5.1.3.2) (UDP-galactose 4-epimerase)
int_rpk_78I15_osl_sgp_std_5p_11S	CK886353	2.40	Bu	No Hit
liv_lrr_05D09_gal_sal_std_5p_11C	BI468170	2.40	Bu	CNS0GSUUTetraodon nigroviridis full-length cDNA
gil_rpk_76B23_osl_sgp_std_5p_11S	CK878429	2.39	Bu	Homo sapiens cDNA clone IMAGE:3939141 **** WARNINGchimeric clone ****
ova_opk_10B09_osl_sgp_std_5p_11S	CK891152	2.39	Bu	No Hit
int_oss_T6P16_osl_sal_std_5p_12C	CK884245	2.38	Bu	hypothetical protein XP_683572
int_rpk_78E01_osl_sgp_std_5p_11S	CK885600	2.37	Bu	(P36220) Tubulin alpha chain (Alpha T6)
hkd_opk_03B15_osl_sgp_std_5p_11C	CK881775	2.36	Bu	(P28072) Proteasome subunit beta type 6 precursor (EC 3.4.25.1)
hkd_opk_04A05_osl_sgp_std_5p_11C	CK880049	2.35	Bu	Abcf2 protein
eye_opk_20B23_osl_sgp_std_5p_11C	CO471165	2.35	Bu	translocon-associated protein beta
eye_rpk_73E23_osl_sgp_std_5p_11C	CK875997	2.31	Bu	(O93277) WD-repeat protein 1 (Actin-interacting protein 1) (AIP1)
int_rpk_76I05_osl_sgp_std_5p_11S	CK885259	2.31	Bu	(Q9YH26) Sodium/potassium-transporting ATPase alpha-1 chain precursor (EC 3.6.3.9)
eye_opk_19L04_osl_sgp_std_5p_11C	CO470723	2.29	Bu	Sfrs2-prov protein
bra_bfo_08E10_fou_sal_nrc_5p_11C	DW589176	2.29	Bu	(Q9PV94) Small nuclear ribonucleoprotein associated protein B' (snRNP-B')
bra_bfo_05E10_fou_sal_nrc_3p_11C	DW588678	2.28	Bu	No Hit
tes_tsr_07A03_gal_sal_std_5p_11C	BM413891	2.28	Bu	(Q6TMA8) Angiopoietin-related protein 4 precursor (Angiopoietin-like 4)
mus_snm_07D12_osl_tra_nrc_5p_11C	EG648535	2.27	Bu	similar to basic leucine zipper and W2 domains 1
spl_sts_18H11_sti_sal_std_5p_12S	AJ425706	2.27	Bu	similar to THUMP domain containing 3 isoform 2

tes_opk_13A22_osl_sgp_std_5p_11C	CK898272	2.27	Bu	(P36972) Adenine phosphoribosyltransferase (EC 2.4.2.7) (APRT)
tes_opk_13E15_osl_sgp_std_5p_11C	CK898447	2.26	Bu	(P38659) Protein disulfide-isomerase A4 precursor (EC 5.3.4.1)
hrt_opk_07M10_osl_sgp_std_5p_11C	CK873927	2.25	Bu	(P14018) Clusterin precursor (51.5 kDa protein)
spl_sts_20D05_sti_sal_std_5p_11C	AJ425827	2.24	Bu	CNS0GGKOTetraodon nigroviridis full-length cDNA
mus_snm_14A12_osl_tra_nrc_5p_11C	EG649113	2.24	Bu	Gasterosteus aculeatus clone CNB264-B03 mRNA sequence
liv_opk_12F12_osl_sgp_std_5p_11C	CK888951	2.23	Bu	(Q6DRH5) H/ACA ribonucleoprotein complex subunit 3
int_rpk_78D02_osl_sgp_std_5p_11S	CK885517	2.22	Bu	(P41182) B-cell lymphoma 6 protein (BCL-6) (Zinc finger protein 51)
gil_rpk_75G11_osl_sgp_std_5p_11C	CK877977	2.22	Bu	(P13184) Cytochrome c oxidase polypeptide VIIa-liver/heart mitochondrial precursor
eye_opk_19E17_osl_sgp_std_5p_11C	CO470324	2.22	Bu	(Q21086) Putative guanine nucleotide binding protein-like 3 homolog (Nucleostemin-1)
hkd_opk_02E04_osl_sgp_std_5p_11C	CK880984	2.22	Bu	(Q13895) Bystin
ova_oyr_02C01_gal_sal_std_5p_11C	BM413951	2.21	Bu	ZPC2
kid_sts_22A04_sti_sal_sub_0p_11C	AJ424875	2.20	Bu	(P63039) 60 kDa heat shock protein mitochondrial precursor (Hsp60)
bra_bfo_06E09_fou_sal_nrc_5p_11M	DW588855	2.19	Bu	(P30352) Splicing factor arginine/serine-rich 2 (Splicing factor SC35) (SC-35)
eye_opk_18D02_osl_sgp_std_5p_11C	CK876449	2.19	Bu	hypothetical protein LOC393239
gil_rpk_74O03_osl_sgp_std_5p_11C	CK879511	2.18	Bu	(Q6AXS5) Plasminogen activator inhibitor 1 RNA-binding protein
kid_aki_01I23_abe_tra_sub_0p_11C	AM412192	2.18	Bu	developmentally regulated GTP binding protein 1
bra_opk_06F23_osl_sgp_std_5p_11S	CK874652	2.18	Bu	No Hit
kid_aki_07D05_abe_tra_sub_0p_11C	AM042499	2.18	Bu	No Hit
mus_mfo_12E12_fou_sal_nrp_5p_11C	DW591803	2.17	Bu	No Hit
ova_oya_01E04_gal_sal_std_5p_11S	No Acc	2.16	Bu	(Q8C3Y4) Kinetochore-associated protein 1
hkd_opk_02E18_osl_sgp_std_5p_11C	CK881026	2.15	Bu	(Q9NZM5) Glioma tumor suppressor candidate region gene 2 protein (p60)
int_rpk_76P04_osl_sgp_std_5p_11C	CK884953	2.14	Bu	CNS0GGKOTetraodon nigroviridis full-length cDNA
hrt_opk_06C24_osl_sgp_std_5p_11C	CK874519	2.14	Bu	(Q9DCA2) 28S ribosomal protein S11 mitochondrial precursor (S11mt) (MRP-S11)
int_rpk_78D05_osl_sgp_std_5p_11C	CK885521	2.14	Bu	similar to cryopyrin isoform b
ova_opk_11G24_osl_sgp_std_5p_11S	CK890364	2.13	Bu	ONU14551Oncorhynchus nerka type-1 growth hormone gene complete cds
bra_snb_04D08_sti_tra_nrc_5p_11C	EG647326	2.12	Bu	(P30352) Splicing factor arginine/serine-rich 2 (Splicing factor SC35) (SC-35)
spl_sts_03F02_sti_sal_std_5p_11C	AJ425034	2.12	Bu	Salmo salar TNF-alpha 2 gene complete cds
tes_opk_12N14_osl_sgp_std_5p_11C	CK898114	2.12	Bu	SMOTC1TPSNSalmo salar transposon-like Tc1-encoded transposase pseudogene
ova_opk_09N12_osl_sgp_std_5p_11C	CK890961	2.10	Bu	(P49962) Signal recognition particle 9 kDa protein (SRP9)
swi_rpk_74C16_osl_sgp_std_5p_11C	CK896794	2.09	Bu	(Q99460) 26S proteasome non-ATPase regulatory subunit 1
kid_opk_01H19_osl_sgp_std_5p_11C	CK887906	2.09	Bu	transposase
int_oss_T6L17_osl_sal_std_5p_22C	CK884144	2.09	Bu	LOC495035 protein
kid_sts_09E12_sti_sal_std_5p_11C	AJ424462	2.09	Bu	hypothetical protein LOC571114
bra_snb_06A01_osl_tra_nrc_5p_11C	EG647776	2.09	Bu	(P16039) Nucleophosmin (NPM) (Nucleolar phosphoprotein B23) (Numatrin)
bra_snb_11B07_osl_tra_nrc_5p_11C	EG647817	2.08	Bu	novel protein (zgc:77026)
hkd_opk_03H11_osl_sgp_std_5p_11C	CK880342	2.08	Bu	No Hit
swi_rpk_74H23_osl_sgp_std_5p_11C	CK895873	2.07	Bu	MGC114881 protein

bra_bfo_07B11_fou_sal_nrc_5p_11M	DW588950		2.07		Bu	(P62146) Calmodulin-alpha (CaM A) (Fragment)
mus_snm_12F02_osl_tra_nrc_5p_11C	EG648990		2.06		Bu	(Q9IB84) Proteasome subunit beta type 1-A (EC 3.4.25.1)
bra_snb_14A01_osl_tra_nrc_5p_11S	EG648061		2.06		Bu	(Q8WQG1) Hypothetical protein C35D10.1 in chromosome III
hkd_opk_02O03_osl_sgp_std_5p_11C	CK881582		2.06		Bu	(Q5RAP1) T-complex protein 1 theta subunit (TCP-1-theta) (CCT-theta)
ova_opk_10F07_osl_sgp_std_5p_11C	CK891361		2.06		Bu	Salmo salar clone BAC S0085O16 partial sequence
gil_oss_G6N24_osl_sal_std_5p_12C	CK877487		2.06		Bu	(P07355) Annexin A2 (Annexin II) (Lipocortin II) (Calpactin I heavy chain)
liv_dis_D4C06_abe_tra_sub_0p_11N	AM049696		2.04		Bu	(P02790) Hemopexin precursor (Beta-1B-glycoprotein)
ova_opk_11P14_osl_sgp_std_5p_11C	CK889740		2.03		Bu	hypothetical protein LOC558738
tes_tsr_04C08_gal_sal_std_5p_11C	No Acc		2.03		Bu	(P01783) Ig heavy chain V region MOPC 21 precursor (Fragment)
liv_dis_D3A11_abe_tra_sub_0p_11N	AM049592		2.03		Bu	(P02790) Hemopexin precursor (Beta-1B-glycoprotein)
gil_oss_52H11_osl_sal_std_5p_11S	CK879342		2.03		Bu	karyopherin alpha 2
ova_opk_09P23_osl_sgp_std_5p_11C	CK891086		2.03		Bu	(Q6NZ06) Interleukin enhancer-binding factor 2 homolog
swi_rpk_74E18_osl_sgp_std_5p_11C	CK897098		2.02		Bu	AY872256S1Oncorhynchus mykiss IgH.A locus partial sequence
kid_opk_01B20_osl_sgp_std_5p_11C	CK887456		2.02		Bu	(Q14697) Neutral alpha-glucosidase AB precursor (EC 3.2.1.84) (
tes_tsr_02D07_gal_sal_std_5p_11C	BM413768		2.02		Bu	(Q9ES56) Trafficking protein particle complex subunit 4 (Synbindin) (TRS23 homolog)
mus_snm_01E06_sti_tra_nrc_5p_11S	EG649232		2.01		Bu	peptidylprolyl isomerase B
bra_bfo_07E10_fou_sal_nrc_5p_11S	DW589022		2.01		Bu	similar to solute carrier family 24 member 4 isoform 1 precursor
bra_bfo_12D11_fou_sal_nrc_5p_11M	DW589779		2.00		Bu	(Q13503) Mediator of RNA polymerase II transcription subunit 21
gil_rpk_75O07_osl_sgp_std_5p_11C	CK878212	407.28		347.90	ACu	CPSF6 protein
eye_opk_19N22_osl_sgp_std_5p_11S	CO470890	22.27		22.46	ACu	No Hit
bra_opk_07F18_osl_sgp_std_5p_11S	CK875291	5.39		4.62	ACu	(O35760) Isopentenyl-diphosphate delta-isomerase 1 (EC 5.3.3.2)
eye_opk_20N21_osl_sgp_std_5p_11C	CO471940	5.09		4.88	ACu	(Q4R4W5) Isopentenyl-diphosphate delta-isomerase 1 (EC 5.3.3.2)
liv_opk_12J23_osl_sgp_std_5p_11C	CK888142	4.94		3.35	ACu	AF232215Salvelinus fontinalis steroidogenic acute regulatory protein (StAR)
int_oss_T5H07_osl_sal_std_5p_11S	CK885907	4.93		5.81	ACu	transposase
liv_lrr_06F01_gal_sal_std_5p_11C	No Acc	4.62		3.11	ACu	(O88803) Leukocyte cell-derived chemotaxin 2 precursor (Chondromodulin II) (ChM-II)
bra_bfo_12B08_fou_sal_nrc_5p_11M	No Acc	4.15		4.74	ACu	No Hit
ova_oyr_07G06_gal_sal_std_5p_11S	No Acc	4.12		2.95	ACu	CK073_XENLAProtein C11orf73 homolog
liv_dis_D2G04_abe_tra_sub_0p_11N	AM049579	3.94		3.83	ACu	chemotaxin
ova_oyr_06H04_gal_sal_std_5p_11C	No Acc	3.13		2.44	ACu	(Q8VCK5) Kelch-like protein 20 (Kelch-like ECT2 interacting protein)
liv_opk_12I04_osl_sgp_std_5p_11C	CK889426	3.10		2.10	ACu	Danio rerio similar to myosin light chain kinase isoform 6 (LOC559667) mRNA
liv_stb_K4E03_sti_tra_sub_0p_11S	AM397505	3.08		2.60	ACu	hypothetical protein XP_683888
swi_rpk_74L20_osl_sgp_std_5p_11S	CK895060	3.07		2.26	ACu	hypothetical protein XP_683888
kid_sts_05A07_sti_sal_std_5p_12C	AJ424263	3.06		2.22	ACu	No Hit
mus_mfo_08G06_fou_sal_nrp_5p_11C	DW591237	3.02		2.19	ACu	(Q61142) Spindlin (30000 Mr metaphase complex) (SSEC P)
ova_oyr_05D05_gal_sal_std_5p_22S	BM414534	2.84		3.18	ACu	No Hit
liv_lrr_08H06_gal_sal_std_5p_11C	BI468047	2.65		3.10	ACu	(P51590) Cytochrome P450 2J3 (EC 1.14.14.1) (CYPIIJ3)

liv_dis_D1G04_abe_tra_sub_0p_11N	AM049505	2.65		2.00	ACu	(O88803) Leukocyte cell-derived chemotaxin 2 precursor (Chondromodulin II) (ChM-II)
liv_opk_12E14_osl_sgp_std_5p_11S	CK888829	2.62		2.10	ACu	AF256661Salmo salar clone BHMS111 microsatellite sequence
tes_tsa_02B07_gal_sal_std_5p_12C	BM413697	2.59		3.39	ACu	(P10775) Ribonuclease inhibitor (Ribonuclease/angiogenin inhibitor 1)
gil_rpk_75P10_osl_sgp_std_5p_11S	CK878443	2.41		2.06	ACu	(Q9YH26) Sodium/potassium-transporting ATPase alpha-1 chain precursor (EC 3.6.3.9)
ova_opk_09N20_osl_sgp_std_5p_11S	CK890974	2.34		5.09	ACu	solute carrier family 25 member 25
int_oss_T5B18_osl_sal_std_5p_11C	CK885962	2.31		2.27	ACu	(P30710) Epididymal secretory glutathione peroxidase precursor (EC 1.11.1.9)
int_oss_T4L23_osl_sal_std_5p_11S	CK884729	2.31		2.04	ACu	No Hit
liv_ali_01D12_abe_tra_sub_0p_11C	AM402478	2.22		2.19	ACu	hypothetical protein XP_683888
liv_opk_12H05_osl_sgp_std_5p_11S	CK889271	2.19		2.11	ACu	No Hit
kid_aki_07A11_abe_tra_sub_0p_11S	AM042467	2.09		2.04	ACu	No Hit
hrt_opk_04E01_osl_sgp_std_5p_11C	CK883183	2.09		3.61	ACu	unnamed protein product
ova_oyr_08B05_gal_sal_std_5p_11C	No Acc	64.77	198.09		ABu	PDZ and LIM domain 2
kid_aki_07D09_abe_tra_sub_0p_11S	AM042502	52.18	124.66		ABu	ONHMHB2MOncorhynchus mykiss beta-2 microglobulin mRNA complete cds
kid_aki_05H02_abe_tra_sub_0p_11C	AM042371	46.22	177.73		ABu	(Q801Y3) Hepcidin 1 precursor
liv_dis_D2G07_abe_tra_sub_0p_11N	AM049581	36.57	61.29		ABu	(Q801Y3) Hepcidin 1 precursor
swi_rpk_74F14_osl_sgp_std_5p_11C	CK895354	22.51	75.32		ABu	unnamed protein product
ova_oyr_06H11_gal_sal_std_5p_11S	BM414441	19.31	31.30		ABu	No Hit
liv_ali_02G12_abe_tra_sub_0p_11C	AM402598	15.77	31.84		ABu	(P10643) Complement component C7 precursor
ova_oyr_07G01_gal_sal_std_5p_11S	BM414518	15.46	10.30		ABu	similar to ORF1 of novel LINE-like retrotransposon
liv_ali_02B04_abe_tra_sub_0p_11C	AM402545	15.36	27.21		ABu	unnamed protein product
kid_aki_04G07_abe_tra_sub_0p_11C	AM042284	13.21	36.68		ABu	(P81491) Serum amyloid A-5 protein
liv_dis_D3B04_abe_tra_sub_0p_11N	AM049608	12.72	7.79		ABu	hypothetical protein XP_678073 isoform 1
kid_aki_07B09_abe_tra_sub_0p_11S	AM042484	12.09	25.67		ABu	*Oncorhynchus mykiss* clone 4b immunoglobulin tau heavy chain secretory form
ova_oyr_04G08_gal_sal_std_5p_11C	BM413968	12.06	6.79		ABu	(Q7ZT42) Staphylococcal nuclease domain containing protein 1 (p100 co-activator)
liv_opk_12H23_osl_sgp_std_5p_11S	CK889408	11.94	11.30		ABu	similar to FLN29 gene product
liv_lra_02F05_gal_sal_std_5p_11C	No Acc	11.90	9.04		ABu	*Oncorhynchus mykiss* haptoglobin mRNA partial cds
ova_oyr_08F04_gal_sal_std_5p_11S	BM414529	11.70	9.93		ABu	similar to Homo sapiens (Human). Mucin 2 precursor (Intestinal mucin 2)
swi_rpk_74D20_osl_sgp_std_5p_11S	CK896849	11.32	2.22		ABu	*Oncorhynchus mykiss* fushi tarazu factor 1 (FTZ-F1) mRNA complete cds
can_TIA_S1B09_sti_tra_can_0p_22N	No Acc	11.09	5.74		ABu	At. sal. TIARPContig Blast SW
spl_opk_15J16_osl_sgp_std_5p_11S	CK894206	11.01	12.76		ABu	chemotaxin
ova_oyr_04H07_gal_sal_std_5p_11C	No Acc	10.93	9.10		ABu	(P52170) Importin alpha-1 subunit (Karyopherin alpha-1 subunit)
swi_rpk_74G16_osl_sgp_std_5p_11S	CK895553	10.85	12.40		ABu	No Hit
swi_rpk_74M02_osl_sgp_std_5p_11C	CK894999	10.69	8.64		ABu	(Q91YW3) DnaJ homolog subfamily C member 3
kid_sts_11B02_sti_sal_std_5p_22C	AJ424600	10.63	6.16		ABu	(O88803) Leukocyte cell-derived chemotaxin 2 precursor (Chondromodulin II) (ChM-II)
bra_snb_08H06_osl_tra_nrc_5p_11C	EG647609	10.55	14.30		ABu	(Q99LE6) ATP-binding cassette sub-family F member 2
can_TIA_S1A02_sti_tra_can_0p_12N	No Acc	10.42	7.03		ABu	At. sal. TIARPContig Blast SW
swi_rpk_74J10_osl_sgp_std_5p_11S	CK896202	10.42	2.09		ABu	No Hit

bra_opk_07B24_osl_sgp_std_5p_11S	CK875117	10.18	7.26	ABu	No Hit
liv_opk_12G04_osl_sgp_std_5p_11C	CK889070	9.78	32.12	ABu	(Q9JLF7) Toll-like receptor 5 precursor
swi_rpk_74D02_osl_sgp_std_5p_11C	CK896753	9.68	6.32	ABu	precerebellin-like protein
liv_dis_D1E05_abe_tra_sub_0p_11N	AM049483	9.36	17.13	ABu	differentially regulated trout protein 1
kid_sts_11B02_sti_sal_std_5p_12C	AJ424600	9.03	5.57	ABu	(O88803) Leukocyte cell-derived chemotaxin 2 precursor (Chondromodulin II) (ChM-II)
swi_rpk_74J02_osl_sgp_std_5p_11S	CK896020	8.95	7.54	ABu	Oncorhynchus mykiss BAC 127C24 partial sequence
mus_mfo_09C08_fou_sal_nrp_5p_11C	DW591296	8.91	6.22	ABu	LOC495205 protein
gil_cgi_D1E08_car_tra_sub_0p_11C	No Acc	8.71	12.00	ABu	differentially regulated trout protein 1
kid_cki_A3G02_car_tra_sub_0p_11S	No Acc	8.42	5.69	ABu	chemotaxin
liv_dis_D1F07_abe_tra_sub_0p_11N	AM049496	8.19	12.15	ABu	C type lectin receptor A
kid_sts_16E02_sti_sal_std_5p_22C	AJ424860	8.13	5.92	ABu	(P62293) Abnormal spindle-like microcephaly-associated protein homolog
liv_dis_D2B03_abe_tra_sub_0p_11N	AM049530	8.04	12.02	ABu	differentially regulated trout protein 1
spl_sts_13B01_sti_sal_std_5p_12S	AJ425436	8.03	6.57	ABu	novel protein
liv_dis_D1G01_abe_tra_sub_0p_11N	AM049499	7.81	12.03	ABu	differentially regulated trout protein 1
ova_opk_11F15_osl_sgp_std_5p_11C	CK890296	7.57	10.91	ABu	No Hit
liv_opk_12H09_osl_sgp_std_5p_11C	CK889309	7.40	5.11	ABu	(Q8BWB6) Six-transmembrane epithelial antigen of prostate 2
ova_oyr_07E08_gal_sal_std_5p_11S	BM414515	7.29	13.39	ABu	(Q9QZI9) Tumor differentially expressed protein 1 (Membrane protein TMS-1)
swi_rpk_74F11_osl_sgp_std_5p_11C	CK896922	7.17	6.50	ABu	growth arrest and DNA-damage-inducible beta
liv_dis_D1D08_abe_tra_sub_0p_11N	AM049476	7.05	11.13	ABu	C type lectin receptor A
liv_dis_D6G11_abe_tra_sub_0p_11N	AM049895	6.88	9.28	ABu	C type lectin receptor A
liv_opk_12L13_osl_sgp_std_5p_11C	CK888444	6.84	4.31	ABu	differentially regulated trout protein 1
int_oss_T4J20_osl_sal_std_5p_11C	CK885171	6.73	5.37	ABu	(O43776) Asparaginyl-tRNA synthetase cytoplasmic (EC 6.1.1.22)
kid_sts_15F02_sti_sal_std_5p_12S	AJ424780	6.66	6.98	ABu	No Hit
hrt_opk_05J02_osl_sgp_std_5p_11S	CK882427	6.63	3.19	ABu	MGC89305 protein
kid_aki_03G01_abe_tra_sub_0p_11C	AM042195	6.56	4.26	ABu	similar to carcinoembryonic antigen-related cell adhesion molecule 5
liv_dis_D2F08_abe_tra_sub_0p_11N	AM049572	6.54	6.34	ABu	Hepcidin-1 precursor
ova_opk_10H18_osl_sgp_std_5p_11C	CK891479	6.47	7.22	ABu	hypothetical protein XP_696848
int_oss_T5E11_osl_sal_std_5p_11C	CK885722	6.24	6.61	ABu	complement protein component C7-1
mus_opk_08L10_osl_sgp_std_5p_11S	CK900534	6.04	2.70	ABu	hypothetical protein LOC386968
mus_snm_01B09_sti_tra_nrc_5p_11S	EG649209	6.00	2.77	ABu	hypothetical protein LOC563855
swi_rpk_74K11_osl_sgp_std_5p_11C	CK895971	5.95	3.33	ABu	(Q01528) Hemagglutinin/amebocyte aggregation factor precursor (18K-LAF)
ova_oyr_05B02_gal_sal_std_5p_11C	BM414350	5.89	5.29	ABu	(O14924) Regulator of G-protein signaling 12 (RGS12)
int_oss_T5G09_osl_sal_std_5p_11C	CK885733	5.83	6.78	ABu	(P56389) Cytidine deaminase (EC 3.5.4.5) (Cytidine aminohydrolase)
bra_snb_04D10_sti_tra_nrc_5p_11C	EG648439	5.81	2.46	ABu	hypothetical protein LOC563855
int_oss_T6L01_osl_sal_std_5p_11C	CK884137	5.68	4.86	ABu	junB protein
gil_agi_05E10_abe_tra_sub_0p_11S	No Acc	5.67	6.76	ABu	No Hit
liv_ali_06G05_abe_tra_sub_0p_11C	AM402932	5.60	3.31	ABu	(Q01528) Hemagglutinin/amebocyte aggregation factor precursor (18K-LAF)

mus_mfo_13B12_fou_sal_nrp_5p_12M	DW591886	5.58	2.36	ABu	(P36871) Phosphoglucomutase-1 (EC 5.4.2.2) (Glucose phosphomutase 1) (PGM 1)
eye_rpk_73F10_osl_sgp_std_5p_11C	CK875912	5.40	3.04	ABu	(P09455) Retinol-binding protein I cellular (Cellular retinol-binding protein) (CRBP)
hrt_opk_05H16_osl_sgp_std_5p_11C	CK882321	5.22	2.32	ABu	hypothetical protein LOC563855
mus_mfo_13B12_fou_sal_nrp_5p_22M	DW591886	5.21	2.51	ABu	(P36871) Phosphoglucomutase-1 (EC 5.4.2.2) (Glucose phosphomutase 1) (PGM 1)
ski_opk_09E20_osl_sgp_std_5p_11S	CK892615	5.17	2.65	ABu	Gasterosteus aculeatus clone CNB181-E03 mRNA sequence
tes_opk_14E09_osl_sgp_std_5p_11S	CK899178	5.16	7.13	ABu	(Q99LE6) ATP-binding cassette sub-family F member 2
int_rpk_76O02_osl_sgp_std_5p_11S	CK885324	5.16	6.45	ABu	No Hit
kid_sts_08B04_sti_sal_std_5p_11C	AJ424328	5.15	5.89	ABu	CNS0GKKMTetraodon nigroviridis full-length cDNA
spl_opk_16G20_osl_sgp_std_5p_11C	CK893548	5.12	2.31	ABu	(P11941) Lysozyme C II precursor (EC 3.2.1.17) (1 4-beta-N-acetylmuramidase C) (Lysozyme type II)
ova_opk_10F17_osl_sgp_std_5p_11C	CK891378	5.12	7.35	ABu	No Hit
spl_opk_17C16_osl_sgp_std_5p_11C	CK893262	5.05	4.00	ABu	complement protein component C7-1
liv_dis_D5E12_abe_tra_sub_0p_11N	AM049791	5.01	5.43	ABu	(Q8AV84) Biotinidase precursor (EC 3.5.1.12)
ski_opk_09D06_osl_sgp_std_5p_11S	CK892487	4.94	2.31	ABu	Danio rerio GATA zinc finger domain containing 2A (gatad2a) mRNA
int_oss_T6P12_osl_sal_std_5p_12C	CK884243	4.90	4.25	ABu	Heat shock protein 9B
int_rpk_78N16_osl_sgp_std_5p_11C	CK884362	4.89	4.66	ABu	(Q9Z1W6) LYRIC protein (Lysine-rich CEACAM1 co-isolated protein)
liv_dis_D4D03_abe_tra_sub_0p_11N	AM049704	4.72	14.50	ABu	toll-like leucine-rich repeat protein precursor
bra_cbr_B3B06_car_tra_sub_0p_11S	No Acc	4.70	5.31	ABu	(P22647) Histone H2A.Z
liv_dis_D5E07_abe_tra_sub_0p_11N	AM049796	4.70	2.57	ABu	Oncorhynchus mykiss SYPG1 (SYPG1) PHF1 (PHF1) and RGL2 (RGL2)
hkd_opk_02L18_osl_sgp_std_5p_11S	CK881451	4.66	3.40	ABu	(P26638) Seryl-tRNA synthetase (EC 6.1.1.11) (Serine--tRNA ligase) (SerRS)
liv_dis_D3B05_abe_tra_sub_0p_11N	AM049609	4.61	2.99	ABu	(Q01528) Hemagglutinin/amebocyte aggregation factor precursor (18K-LAF)
tes_tsr_06C02_gal_sal_std_5p_11S	BM413921	4.60	7.85	ABu	(Q9UG63) ATP-binding cassette sub-family F member 2
bra_opk_07I07_osl_sgp_std_5p_11S	CK873695	4.55	3.21	ABu	Oncorhynchus keta IT-I gene for isotocin complete cds
kid_aki_06C10_abe_tra_sub_0p_11C	AM042401	4.52	4.43	ABu	Danio rerio eukaryotic translation initiation factor 4A isoform 1A (eif4a1a) mRNA
liv_ali_02G03_abe_tra_sub_0p_11C	AM402600	4.49	4.39	ABu	(Q61656) Probable RNA-dependent helicase p68 (DEAD-box protein p68)
ova_oyr_08D04_gal_sal_std_5p_11C	BM414056	4.44	7.08	ABu	(Q01081) Splicing factor U2AF 35 kDa subunit
liv_opk_12I09_osl_sgp_std_5p_11C	CK889466	4.43	3.28	ABu	(Q90YT6) 60S ribosomal protein L32
ova_oyr_08C10_gal_sal_std_5p_22S	BM414055	4.38	2.43	ABu	(P06238) Alpha-2-macroglobulin precursor (Alpha-2-M)
eye_opk_17I15_osl_sgp_std_5p_11C	CK876915	4.26	3.03	ABu	hypothetical protein
tes_tsr_04B01_gal_sal_std_5p_11C	BM413798	4.25	3.56	ABu	(P35031) Trypsin I precursor (EC 3.4.21.4)
gil_oss_G5G20_osl_sal_std_5p_11S	CK878951	4.14	2.22	ABu	(Q9P2E5) Chondroitin sulfate glucuronyltransferase (EC 2.4.1.226)
int_oss_THB11_osl_sal_std_5p_11C	CN181327	4.13	3.17	ABu	Salmo salar BAC S0188I22 partial sequence
hkd_opk_02L16_osl_sgp_std_5p_11C	CK881446	4.12	3.14	ABu	No Hit
swi_rpk_74B19_osl_sgp_std_5p_11S	CK896396	4.08	2.07	ABu	Schistosoma japonicum CA protein mRNA complete cds
liv_lrr_06E02_gal_sal_std_5p_11C	No Acc	4.04	3.68	ABu	(Q90WJ9) Saxitoxin and tetrodotoxin binding protein 2 precursor
gil_oss_G5J20_osl_sal_std_5p_11S	CK879095	4.01	2.20	ABu	(O88878) Zinc finger A20 domain containing protein 2 (Zinc finger protein 216)
mus_snm_08E09_osl_tra_nrc_5p_11C	EG648636	4.00	2.63	ABu	Oncorhynchus mykiss calreticulin (CRT) mRNA complete cds
gil_oss_G5P19_osl_sal_std_5p_11C	CK879052	3.95	2.39	ABu	hypothetical protein XP_692860

ova_opk_11J17_osl_sgp_std_5p_11C	CK890507	3.90	5.83	ABu	similar to hemicentin 1 partial
mus_mfo_09B07_fou_sal_nrp_5p_11S	DW591274	3.86	3.73	ABu	similar to CG4646-PA
spl_opk_16B23_osl_sgp_std_5p_11C	CK894762	3.85	4.19	ABu	No Hit
hrt_opk_08P01_osl_sgp_std_5p_11C	CK892065	3.84	2.62	ABu	CCAAT/enhancer-binding protein beta
kid_aki_03C07_abe_tra_sub_0p_11C	AM042161	3.81	2.64	ABu	putative serum transporter protein
int_oss_T4G21_osl_sal_std_5p_11C	CK885082	3.78	2.22	ABu	CD63-like protein
hrt_opk_06H02_osl_sgp_std_5p_11C	CK874696	3.74	2.97	ABu	cytidylate kinase
gil_oss_G6A23_osl_sal_std_5p_11C	CK877180	3.73	2.42	ABu	(P62909) 40S ribosomal protein S3
liv_opk_12D07_osl_sgp_std_5p_11C	CK888622	3.70	7.44	ABu	(O15431) High-affinity copper uptake protein 1 (hCTR1)
bra_snb_06G10_osl_tra_nrc_5p_11S	EG648175	3.69	4.55	ABu	unnamed protein product
hrt_opk_09B03_osl_sgp_std_5p_11C	CK892289	3.64	2.78	ABu	(P79703) Transcription factor jun-B
tes_opk_15I05_osl_sgp_std_5p_11S	CK897337	3.62	3.84	ABu	No Hit
ova_opk_11H16_osl_sgp_std_5p_11C	CK890396	3.57	2.67	ABu	(P49924) Fatty acid-binding protein liver (L-FABP)
kid_opk_01H20_osl_sgp_std_5p_11S	CK887907	3.57	3.72	ABu	(P08110) Endoplasmin precursor (Heat shock 108 kDa protein Transferrin-binding protein)
liv_opk_12F23_osl_sgp_std_5p_11S	CK889033	3.54	3.07	ABu	Gasterosteus aculeatus clone CFW280-A10 mRNA sequence
kid_aki_02B12_abe_tra_sub_0p_11S	AM042065	3.54	2.55	ABu	(Q9GZP9) Derlin-2 (Der1-like protein 2) (DERtrin-2) (F-LANa) (F-LAN-1)
kid_opk_01B10_osl_sgp_std_5p_11S	CK887410	3.51	2.77	ABu	Salmo salar BAC S0188I22 partial sequence
swi_rpk_74I13_osl_sgp_std_5p_11C	CK895653	3.51	3.64	ABu	(P13667) Protein disulfide-isomerase A4 precursor (EC 5.3.4.1) (Protein ERp-72)
gil_oss_G6P19_osl_sal_std_5p_11C	CK877417	3.50	2.35	ABu	(P29350) Tyrosine-protein phosphatase non-receptor type 6 (EC 3.1.3.48)
gil_oss_52F06_osl_sal_std_5p_11S	CK879413	3.48	3.73	ABu	Unknown (protein for IMAGE:7406658)
bra_opk_05L08_osl_sgp_std_5p_11S	CK874173	3.40	3.58	ABu	(Q9UJF2) Ras GTPase-activating protein nGAP (RAS protein activator-like 1)
hrt_opk_06J17_osl_sgp_std_5p_11C	CK874795	3.39	4.27	ABu	(Q07021) Complement component 1 Q subcomponent binding protein
spl_opk_16L18_osl_sgp_std_5p_11C	CK893980	3.39	3.54	ABu	tissue inhibitor of metalloproteinase 2
int_oss_T4L04_osl_sal_std_5p_11C	CK885174	3.38	2.44	ABu	(O54873) Multisynthetase complex auxiliary component p43
ova_oyr_08B12_gal_sal_std_5p_11C	BM414456	3.37	2.07	ABu	(Q78P75) Dynein light chain 2 cytoplasmic
gil_oss_GHK08_osl_sal_std_5p_12S	CN181225	3.33	2.95	ABu	hypothetical protein LOC570589
liv_opk_12H11_osl_sgp_std_5p_11C	CK889324	3.28	4.75	ABu	No Hit
mus_snm_11D06_osl_tra_nrc_5p_11C	EG648891	3.27	2.44	ABu	No Hit
gil_rpk_75H23_osl_sgp_std_5p_11C	CK877791	3.27	2.71	ABu	Salmo salar BAC S0188I22 partial sequence
liv_lra_02F08_gal_sal_std_5p_11C	No Acc	3.24	3.26	ABu	(P36992) AMBP protein precursor
ova_opk_11I05_osl_sgp_std_5p_11C	CK890422	3.24	2.65	ABu	Canis familiaris similar to stromal cell derived factor 4 transcript variant 1 (LOC479572)
liv_opk_12L12_osl_sgp_std_5p_11C	CK888443	3.24	3.02	ABu	putative serum transporter protein
hrt_opk_06E09_osl_sgp_std_5p_11C	CK874584	3.23	2.57	ABu	unnamed protein product
bra_cbr_B1F10_car_tra_sub_0p_11C	EG354852	3.21	2.53	ABu	Unknown (protein for IMAGE:7406658)
kid_cki_A4D09_car_tra_sub_0p_11C	No Acc	3.21	2.48	ABu	AY872256S1Oncorhynchus mykiss IgH.A locus partial sequence
bra_bfo_15B07_fou_sal_nrp_5p_11S	No Acc	3.20	3.08	ABu	Danio rerio ATP-binding cassette sub-family E (OABP) member 1 (abce1) mRNA
liv_ali_02G02_abe_tra_sub_0p_11S	AM402599	3.19	2.19	ABu	(Q8TCT9) Minor histocompatibility antigen H13 (EC 3.4.99.-)

bra_snb_14E07_osl_tra_nrc_5p_11C	EG648113	3.13	2.76	ABu	Unknown (protein for IMAGE:7406658)
liv_ali_06C01_abe_tra_sub_0p_11C	AM402888	3.12	3.39	ABu	Danio rerio hypoxia up-regulated 1 (hyou1) mRNA
kid_aki_03F09_abe_tra_sub_0p_11S	AM042194	3.10	2.97	ABu	(Q29561) UMP-CMP kinase (EC 2.7.4.14) (Cytidylate kinase)
liv_dis_D4A07_abe_tra_sub_0p_11N	AM049678	3.09	7.36	ABu	C type lectin receptor A
hrt_opk_04M04_osl_sgp_std_5p_11S	CK883775	3.07	2.58	ABu	activated leukocyte cell adhesion molecule
int_oss_T5P09_osl_sal_std_5p_11S	CK885953	3.06	2.95	ABu	unnamed protein product
int_oss_T4I13_osl_sal_std_5p_11C	CK885089	3.06	2.25	ABu	(P05619) Leukocyte elastase inhibitor (LEI) (Serpin B1)
hrt_opk_07I05_osl_sgp_std_5p_11C	CK873690	3.06	2.97	ABu	Protein transport protein Sec61 subunit alpha isoform B
kid_sts_10D11_sti_sal_std_5p_11C	AJ424542	3.04	3.00	ABu	No Hit
liv_lrr_08A08_gal_sal_std_5p_11C	No Acc	3.04	3.59	ABu	No Hit
kid_sts_10E08_sti_sal_std_5p_11C	AJ424551	3.02	3.30	ABu	(P17676) CCAAT/enhancer binding protein beta (C/EBP beta) (Nuclear factor NF-IL6)
liv_ali_01A08_abe_tra_sub_0p_11C	AM402452	2.99	3.17	ABu	(Q9Y285) Phenylalanyl-tRNA synthetase alpha chain (EC 6.1.1.20)
bra_opk_06K21_osl_sgp_std_5p_11S	CK874844	2.98	2.87	ABu	(Q9CR60) UPF0198 protein CGI-141 homolog
mus_snm_01F10_sti_tra_nrc_5p_11C	No Acc	2.98	2.54	ABu	methylenetetrahydrofolate dehydrogenase (NADP+ dependent)
hrt_opk_05O08_osl_sgp_std_5p_11C	CK874328	2.97	3.73	ABu	(Q8VC88) Grancalcin
int_rpk_78A15_osl_sgp_std_5p_11S	CK885405	2.96	2.42	ABu	Salmo salar clone BAC S0085O16 partial sequence
mus_snm_07B11_osl_tra_nrc_5p_11C	No Acc	2.95	2.65	ABu	No Hit
ova_oyr_02C07_gal_sal_std_5p_11C	BM414412	2.95	5.98	ABu	No Hit
kid_opk_01I06_osl_sgp_std_5p_11S	CK887928	2.93	2.30	ABu	AF256953Salmo salar clone BHMS391 microsatellite sequence
liv_ali_01H11_abe_tra_sub_0p_11C	AM402518	2.93	3.28	ABu	(Q8AV84) Biotinidase precursor (EC 3.5.1.12)
kid_opk_01M06_osl_sgp_std_5p_11S	CK887115	2.92	2.06	ABu	(O14792) Heparan sulfate glucosamine 3-O-sulfotransferase 1 precursor (EC 2.8.2.23)
gil_oss_52A18_osl_sal_std_5p_11C	CK879219	2.90	2.70	ABu	(P03974) Transitional endoplasmic reticulum ATPase
ova_opk_09J03_osl_sgp_std_5p_11C	CK890754	2.88	2.87	ABu	(P53845) Hypothetical 35.5 kDa protein in PIK1-POL2 intergenic region
kid_opk_01O18_osl_sgp_std_5p_11C	CK886619	2.86	2.80	ABu	(O89079) Coatomer epsilon subunit (Epsilon-coat protein) (Epsilon-COP)
hrt_opk_04F14_osl_sgp_std_5p_11C	CK883277	2.83	2.17	ABu	AChain A Human Ubiquitin-Conjugating Enzyme (E2) Ubch5b Wild-Type
hkd_opk_03M23_osl_sgp_std_5p_11C	CK882261	2.82	3.21	ABu	Prmt1 protein
mus_mfo_06D03_fou_sal_nrp_5p_11M	DW590904	2.82	4.87	ABu	fast myotomal muscle tropomyosin
gil_oss_G5K21_osl_sal_std_5p_11S	CK878898	2.82	2.03	ABu	(O35142) Coatomer beta' subunit (Beta'-coat protein) (Beta'-COP) (p102)
hkd_opk_02O18_osl_sgp_std_5p_11C	CK881612	2.80	3.87	ABu	(P16967) Translocon-associated protein alpha subunit precursor (TRAP-alpha)
hrt_opk_08D15_osl_sgp_std_5p_11C	CK873530	2.79	3.40	ABu	Oncorhynchus tshawytscha virus-inducible stress protein (VISP) mRNA complete cds
ova_opk_09P08_osl_sgp_std_5p_11C	CK891050	2.78	2.36	ABu	Salmo salar clone Rsa423 microsatellite sequence
hrt_opk_05N21_osl_sgp_std_5p_11C	CK874303	2.77	2.97	ABu	CNS0G068Tetraodon nigroviridis full-length cDNA
gil_oss_G6C19_osl_sal_std_5p_11C	CK877190	2.76	2.11	ABu	insulin-like growth factor binding protein 6
mus_mfo_1bE04_fou_sal_nrp_5p_11C	DW590373	2.76	3.13	ABu	(P60060) Protein transport protein SEC61 gamma subunit
kid_aki_02C03_abe_tra_sub_0p_11C	AM042078	2.74	2.69	ABu	AF394686_1C1q-like adipose specific protein
hkd_opk_03E05_osl_sgp_std_5p_11C	CK881965	2.74	2.02	ABu	CD40
ova_opk_11C15_osl_sgp_std_5p_11C	CK890160	2.74	3.93	ABu	heat shock 60 kD protein 1

kid_aki_07C05_abe_tra_sub_0p_11S	AM042490	2.72	2.71	ABu	No Hit
bra_snb_10F11_osl_tra_nrc_5p_11C	EG647775	2.71	2.57	ABu	(O43776) Asparaginyl-tRNA synthetase cytoplasmic (EC 6.1.1.22)
int_oss_T6L17_osl_sal_std_5p_12C	CK884144	2.70	2.45	ABu	LOC495035 protein
kid_aki_05A09_abe_tra_sub_0p_11C	AM042307	2.70	2.14	ABu	(Q61656) Probable RNA-dependent helicase p68 (DEAD-box protein p68)
liv_lrr_03F06_gal_sal_std_5p_11S	BI468136	2.70	2.03	ABu	No Hit
spl_opk_16I13_osl_sgp_std_5p_11C	CK893706	2.69	4.12	ABu	(Q8AXL1) Diamine acetyltransferase 1 (EC 2.3.1.57)
tes_tsa_02D12_gal_sal_std_5p_11S	BM414103	2.69	2.93	ABu	(Q9Y4A8) Nuclear factor erythroid 2 related factor 3 (NF-E2 related factor 3)
mus_snm_09G08_osl_tra_nrc_5p_11C	EG648755	2.67	2.32	ABu	novel protein similar to human general control of amino-acid synthesis 1-like 1 (yeast)
hkd_opk_03N14_osl_sgp_std_5p_11C	CK880657	2.65	3.34	ABu	hypothetical protein XP_697426
ova_oyr_07G12_gal_sal_std_5p_11C	BM414449	2.64	3.04	ABu	Lsm11 protein
mus_snm_07B03_osl_tra_nrc_5p_11C	EG648455	2.64	3.44	ABu	Salmo salar clone Hae416 microsatellite sequence
liv_dis_D1D03_abe_tra_sub_0p_11N	AM049472	2.64	3.14	ABu	C type lectin receptor B
bra_opk_07I06_osl_sgp_std_5p_11S	CK873693	2.63	2.11	ABu	Salmo salar clone 78a genomic sequence
ova_opk_11J11_osl_sgp_std_5p_11C	CK890485	2.63	2.57	ABu	similar to interleukin-4 receptor alpha-chain
mus_snm_13H11_osl_tra_nrc_5p_11S	EG649101	2.63	2.30	ABu	No Hit
liv_dis_D2H05_abe_tra_sub_0p_11N	AM049588	2.61	2.60	ABu	(Q8AV84) Biotinidase precursor (EC 3.5.1.12)
gil_oss_G6J12_osl_sal_std_5p_11S	CK877461	2.60	2.72	ABu	No Hit
bra_bfo_03D07_fou_sal_nrc_5p_11M	DW588384	2.60	2.20	ABu	(P17844) Probable RNA-dependent helicase p68 (DEAD-box protein p68)
hkd_opk_03G08_osl_sgp_std_5p_11C	CK882106	2.59	2.37	ABu	(O35593) 26S proteasome non-ATPase regulatory subunit 14 (26S proteasome regulatory subunit rpn11)
liv_ali_01A12_abe_tra_sub_0p_11S	AM402447	2.58	3.04	ABu	angiotensinogen precursor
kid_opk_01J22_osl_sgp_std_5p_11S	CK886926	2.58	4.63	ABu	(P54996) Glucose-6-phosphate 1-dehydrogenase (EC 1.1.1.49)
kid_sts_15C04_sti_sal_std_5p_11C	AJ424747	2.58	2.36	ABu	(Q6IQM2) Cytochrome c
kid_sts_09D11_sti_sal_std_5p_11C	AJ424449	2.57	2.48	ABu	Salmo salar clone Rsa454 microsatellite sequence
kid_aki_03F11_abe_tra_sub_0p_11S	AM412203	2.54	2.00	ABu	Ddx5 protein
hkd_opk_02E19_osl_sgp_std_5p_11C	CK881027	2.54	2.19	ABu	Danio rerio similar to eukaryotic translation initiation factor 4 gamma 1 isoform 4 (LOC572435) mRNA
kid_aki_02G04_abe_tra_sub_0p_11C	AM042119	2.54	2.64	ABu	Salmo salar clone Alu367 microsatellite sequence
spl_opk_16L17_osl_sgp_std_5p_11C	CK893979	2.53	2.52	ABu	Danio rerio zgc:77725 mRNA (cDNA clone MGC:77725 IMAGE:7000702) complete cds
gil_oss_GHG15_osl_sal_std_5p_11S	CN181072	2.51	2.03	ABu	Salmo salar clone Rsa426 microsatellite sequence
mus_snm_04E08_sti_tra_nrc_5p_11C	EG649455	2.50	2.88	ABu	(Q9CQU3) RER1 protein
kid_aki_03G07_abe_tra_sub_0p_11C	AM042204	2.48	2.15	ABu	Tmc6-related protein 1
ova_oyr_05A11_gal_sal_std_5p_11S	BM414494	2.47	3.52	ABu	No Hit
hrt_opk_05E22_osl_sgp_std_5p_11C	CK882976	2.47	2.22	ABu	tumor protein D52-like 2 isoform 1
kid_aki_05C03_abe_tra_sub_0p_11C	AM042321	2.46	2.01	ABu	Setb protein
kid_aki_01O09_abe_tra_sub_0p_11C	AM042049	2.45	2.33	ABu	(Q9Y221) 60S ribosome subunit biogenesis protein NIP7 homolog (KD93)
gil_agi_06F09_abe_tra_sub_0p_11C	AM041878	2.44	2.53	ABu	(Q62737) Cytochrome b-245 light chain (p22 phagocyte B-cytochrome)
ova_oyr_02A05_gal_sal_std_5p_11S	BM414015	2.43	2.55	ABu	(Q9UJW8) Zinc finger protein 180 (HHZ168)

kid_opk_01J20_osl_sgp_std_5p_11C	CK886920	2.42	2.96	ABu	(P09803) Epithelial-cadherin precursor (E-cadherin) (Uvomorulin) (Cadherin-1) (ARC-1)
bra_bfo_05F09_fou_sal_nrc_3p_11M	DW588701	2.42	2.18	ABu	No Hit
spl_sts_03B01_sti_sal_std_5p_11C	AJ424987	2.42	4.02	ABu	(P25963) NF-kappaB inhibitor alpha
tes_opk_13H11_osl_sgp_std_5p_11C	CK898599	2.42	2.86	ABu	(Q9H7B2) Brix domain containing protein 1
int_oss_T6M06_osl_sal_std_5p_11C	CK884070	2.39	3.04	ABu	(Q4AEI0) Glutathione peroxidase 2 (EC 1.11.1.9)
gil_rpk_75I22_osl_sgp_std_5p_11S	CK877675	2.39	2.17	ABu	No Hit
int_oss_T6P12_osl_sal_std_5p_22C	CK884243	2.39	2.10	ABu	Heat shock protein 9B
ova_opk_11F17_osl_sgp_std_5p_11C	CK890298	2.38	3.36	ABu	(Q8UVY2) Brix domain containing protein 2 (Ribosome biogenesis protein Brix)
eye_rpk_73J22_osl_sgp_std_5p_11C	CO472327	2.38	2.84	ABu	No Hit
hrt_opk_05J18_osl_sgp_std_5p_11C	CK882487	2.38	2.06	ABu	(O13085) Cytochrome c oxidase polypeptide VIa mitochondrial precursor (EC 1.9.3.1)
liv_lrr_08C08_gal_sal_std_5p_11C	BI468069	2.38	2.15	ABu	(Q99NB9) Splicing factor 3B subunit 1
mus_snm_12B12_osl_tra_nrc_5p_11S	EG648956	2.36	2.62	ABu	(Q5RA42) Eukaryotic translation initiation factor 1A X-chromosomal (eIF-1A X isoform)
spl_sts_13D09_sti_sal_std_5p_11C	AJ425467	2.36	2.73	ABu	(P56537) Eukaryotic translation initiation factor 6 (eIF-6) (B4 integrin interactor)
kid_opk_01E01_osl_sgp_std_5p_11C	CK887656	2.35	2.19	ABu	ribosomal protein L7-like 1
tes_opk_14M10_osl_sgp_std_5p_11C	CK897633	2.35	3.16	ABu	(Q8AWW7) RuvB-like 1 (EC 3.6.1.-) (Pontin) (zPontin)
gil_rpk_74N24_osl_sgp_std_5p_11C	CK879507	2.35	2.89	ABu	(P16967) Translocon-associated protein alpha subunit precursor (TRAP-alpha)
liv_lrr_03B01_gal_sal_std_5p_11S	No Acc	2.35	3.08	ABu	Gasterosteus aculeatus clone CEC21-F12 mRNA sequence
mus_snm_10B06_osl_tra_nrc_5p_11C	EG648787	2.35	2.09	ABu	(Q9CR68) Ubiquinol-cytochrome c reductase iron-sulfur subunit mitochondrial precursor (EC 1.10.2.2)
hkd_opk_03M24_osl_sgp_std_5p_11C	CK882262	2.35	2.83	ABu	Unknown (protein for MGC:147239)
bra_snb_12D05_osl_tra_nrc_5p_11C	EG647917	2.35	2.01	ABu	(Q5R5F2) Coatomer zeta-1 subunit (Zeta-1 coat protein) (Zeta-1 COP)
spl_opk_15K12_osl_sgp_std_5p_11C	CK894255	2.34	2.25	ABu	Danio rerio zgc:65781 (zgc:65781) mRNA
liv_opk_12J20_osl_sgp_std_5p_11S	CK888120	2.34	3.46	ABu	Salmo salar BAC S0188I22 partial sequence
gil_rpk_75K09_osl_sgp_std_5p_11C	CK877760	2.34	2.09	ABu	CNS0G0LPTetraodon nigroviridis full-length cDNA
bra_snb_04F11_sti_tra_nrc_5p_11C	EG647341	2.33	2.73	ABu	(P48721) Stress-70 protein mitochondrial precursor (75 kDa glucose regulated protein)
swi_rpk_74L11_osl_sgp_std_5p_11C	CK896270	2.32	2.25	ABu	No Hit
bra_snb_03D09_sti_tra_nrc_5p_11S	EG648362	2.32	2.34	ABu	(Q99873) Protein arginine N-methyltransferase 1 (EC 2.1.1.-) (Interferon receptor 1-bound protein 4)
kid_aki_03H03_abe_tra_sub_0p_11C	AM042210	2.31	2.74	ABu	CNS0FO4STetraodon nigroviridis full-length cDNA
can_PPa_S1B12_sti_tra_can_0p_22N	No Acc	2.31	2.04	ABu	At. sal. PPARa Contig Blast SW
bra_snb_06A09_osl_tra_nrc_5p_22C	EG647428	2.31	2.52	ABu	(Q13283) Ras-GTPase-activating protein binding protein 1
mus_amu_08E05_abe_tra_sub_0p_11C	AM083816	2.30	2.13	ABu	No Hit
kid_aki_07D12_abe_tra_sub_0p_11C	AM042496	2.30	2.28	ABu	No Hit
bra_bfo_15C05_fou_sal_nrp_5p_11M	DW590092	2.29	2.37	ABu	hypothetical protein LOC550424
spl_sts_06D10_sti_sal_std_5p_11C	AJ425205	2.29	2.42	ABu	(Q16270) Insulin-like growth factor binding protein 7 precursor
ova_opk_10G19_osl_sgp_std_5p_11C	CK891441	2.28	2.36	ABu	(P45878) FK506-binding protein 2 precursor (EC 5.2.1.8) (Peptidyl-prolyl cis-trans isomerase)
swi_rpk_74C07_osl_sgp_std_5p_11C	CK896558	2.28	2.03	ABu	(P46978) Oligosaccharyl transferase STT3 subunit homolog (B5) (Integral membrane protein 1)
bra_snb_01E07_sti_tra_nrc_5p_11C	EG648201	2.26	2.06	ABu	(Q5RF83) Cold-inducible RNA-binding protein (Glycine-rich RNA-binding protein CIRP)

bra_snb_03E06_sti_tra_nrc_5p_11C	EG648369	2.26	2.44	ABu	(Q91060) Tubulin alpha chain
ova_opk_10N06_osl_sgp_std_5p_11C	CK891768	2.26	3.04	ABu	(Q9Y3B3) Transmembrane emp24 domain containing protein 7 precursor
int_rpk_78K11_osl_sgp_std_5p_11C	CK886518	2.25	2.62	ABu	(P30944) Epithelial-cadherin precursor (E-cadherin) (Uvomorulin) (xTCAD-1)
mus_snm_04G09_sti_tra_nrc_5p_11C	EG649476	2.25	2.53	ABu	ATP-binding cassette sub-family E (OABP) member 1
gil_rpk_75D22_osl_sgp_std_5p_11S	CK879927	2.25	3.00	ABu	(Q9WUU8) Nef-associated factor 1 (Naf1) (TNFAIP3-interacting protein 1)
liv_str_02C09_sti_tra_sub_0p_11C	AM397516	2.25	2.89	ABu	hypothetical protein LOC402979
hkd_opk_04D01_osl_sgp_std_5p_11S	CK880259	2.24	2.36	ABu	(P43690) Developmentally regulated GTP-binding protein 1 (DRG 1) (xDRG)
spl_opk_15P03_osl_sgp_std_5p_11C	CK894576	2.22	2.24	ABu	CNS0GRLATetraodon nigroviridis full-length cDNA
int_rpk_76H14_osl_sgp_std_5p_11C	CK884783	2.21	2.46	ABu	(Q28104) Coatomer epsilon subunit (Epsilon-coat protein) (Epsilon-COP)
gil_agi_01D08_abe_tra_sub_0p_11C	AM041450	2.21	2.63	ABu	CNS0GFDKTetraodon nigroviridis full-length cDNA
hkd_opk_02I04_osl_sgp_std_5p_11C	CK881242	2.20	2.11	ABu	(P68101) Eukaryotic translation initiation factor 2 subunit 1
kid_sts_22E06_sti_sal_sub_0p_11C	AJ424948	2.20	2.20	ABu	Prmt1 protein
tes_opk_13B22_osl_sgp_std_5p_11C	CK898326	2.20	2.86	ABu	(Q9NX57) Ras-related protein Rab-20
bra_bfo_06G12_fou_sal_nrc_5p_11C	DW588905	2.20	2.43	ABu	(P46462) Transitional endoplasmic reticulum ATPase (TER ATPase)
hrt_opk_07B23_osl_sgp_std_5p_11C	CK875111	2.19	2.26	ABu	nucleolar protein family A member 1 (H/ACA small nucleolar RNPs)
int_oss_T6L13_osl_sal_std_5p_11C	CK884142	2.19	2.25	ABu	NOP56
ova_oyr_06E10_gal_sal_std_5p_22C	BM414402	2.18	2.17	ABu	AHA1 activator of heat shock 90kDa protein ATPase homolog 1 like
mus_mfo_08E10_fou_sal_nrp_5p_11S	DW591215	2.17	2.14	ABu	CNS0G0FRTetraodon nigroviridis full-length cDNA
mus_snm_13A08_osl_tra_nrc_5p_11C	No Acc	2.17	2.63	ABu	zeta1-cop
ova_oyr_04F04_gal_sal_std_5p_11S	BM414488	2.16	2.12	ABu	(Q8C863) Itchy E3 ubiquitin protein ligase (EC 6.3.2.-)
mus_snm_08E02_osl_tra_nrc_5p_11C	EG648630	2.16	2.17	ABu	tubulin alpha 8 like 2
ova_oyr_03B09_gal_sal_std_5p_11C	No Acc	2.15	2.12	ABu	(Q96S59) Ran-binding protein 9 (RanBP9) (RanBP7)
kid_aki_04F06_abe_tra_sub_0p_11C	AM042271	2.15	2.22	ABu	(Q68F90) Nascent polypeptide-associated complex alpha subunit
int_oss_T5E08_osl_sal_std_5p_11C	CK885807	2.14	2.90	ABu	(P63039) 60 kDa heat shock protein mitochondrial precursor (Hsp60)
gil_oss_GHJ14_osl_sal_std_5p_11S	CN181085	2.14	2.16	ABu	similar to TAF15 RNA polymerase II TATA box binding protein (TBP)-associated factor
ova_opk_10H16_osl_sgp_std_5p_11S	CK891477	2.14	2.16	ABu	Salmo salar clone Rsa454 microsatellite sequence
mus_mfo_10A03_fou_sal_nrp_3p_11S	DW591419	2.13	2.29	ABu	No Hit
hkd_opk_03C03_osl_sgp_std_5p_11C	CK881811	2.13	2.10	ABu	Danio rerio ribophorin I mRNA complete cds
mus_snm_10E10_osl_tra_nrc_5p_11C	EG648827	2.10	5.08	ABu	Gasterosteus aculeatus clone CNB269-C04 mRNA sequence
kid_aki_06D07_abe_tra_sub_0p_11C	AM042419	2.08	2.11	ABu	Danio rerio zgc:77725 mRNA (cDNA clone MGC:77725 IMAGE:7000702) complete cds
kid_opk_01N04_osl_sgp_std_5p_11C	CK887193	2.08	2.05	ABu	Danio rerio zgc:77153 mRNA (cDNA clone IMAGE:3819191) partial cds
gil_oss_G5J02_osl_sal_std_5p_11C	CK879087	2.07	2.05	ABu	G1 to S phase transition 1
bra_snb_02H01_sti_tra_nrc_5p_11S	EG648314	2.07	2.17	ABu	MGC53562 protein
ova_opk_09N06_osl_sgp_std_5p_11S	CK890950	2.07	2.41	ABu	Eukaryotic translation initiation factor 4E
int_rpk_76K07_osl_sgp_std_5p_11C	CK884579	2.07	2.01	ABu	cox4 neighbor
mus_mfo_14H04_fou_sal_nrp_5p_11M	DW592129	2.06	2.23	ABu	(P60060) Protein transport protein SEC61 gamma subunit
ova_opk_11J22_osl_sgp_std_5p_11C	CK890517	2.06	2.01	ABu	novel protein similar to human and mouse solute carrier family 30

liv_opk_12G23_osl_sgp_std_5p_11C	CK889207	2.05	2.38		ABu	No Hit
bra_bfo_08C01_fou_sal_nrc_5p_11M	DW589115	2.05	2.43		ABu	(Q9CX56) 26S proteasome non-ATPase regulatory subunit 8 (26S proteasome regulatory subunit S14)
eye_opk_17P14_osl_sgp_std_5p_11S	CK876231	2.03	2.32		ABu	Prmt5 protein
bra_snb_07H07_osl_tra_nrc_5p_11C	EG647520	2.03	2.87		ABu	(P25489) Sodium/potassium-transporting ATPase alpha-1 chain precursor (EC 3.6.3.9)
kid_aki_05A08_abe_tra_sub_0p_11C	AM042306	29.57	12.79	3.63	ABCu	(P06761) 78 kDa glucose-regulated protein precursor (GRP 78) (BiP)
liv_ali_06D10_abe_tra_sub_0p_11C	AM402899	22.02	13.04	2.72	ABCu	(Q90593) 78 kDa glucose-regulated protein precursor (GRP 78) (BiP)
hrt_opk_07B22_osl_sgp_std_5p_11C	CK875110	20.36	10.47	2.44	ABCu	Oncorhynchus mykiss SYPG1 (SYPG1) PHF1 (PHF1) and RGL2 (RGL2) genes complete cds;
ova_oyr_07F12_gal_sal_std_5p_11C	BM414341	17.70	6.39	2.85	ABCu	(Q9UMY4) Sorting nexin-12
liv_ali_02D09_abe_tra_sub_0p_11C	AM402572	16.23	6.86	2.15	ABCu	(P55083) Microfibril-associated glycoprotein 4 precursor
liv_dis_D1E12_abe_tra_sub_0p_11N	AM049479	16.18	6.74	2.79	ABCu	similar to catechol-O-methyltransferase domain containing 1
liv_dis_D3C06_abe_tra_sub_0p_11N	AM049619	16.06	6.01	3.14	ABCu	(Q40313) Caffeoyl-CoA O-methyltransferase (EC 2.1.1.104)
hrt_opk_04E05_osl_sgp_std_5p_11C	CK883188	15.54	7.86	2.57	ABCu	DnaJ-like subfamily B member 11
liv_dis_D3C01_abe_tra_sub_0p_11N	AM049614	15.22	5.40	2.77	ABCu	(Q40313) Caffeoyl-CoA O-methyltransferase (EC 2.1.1.104)
liv_dis_D4H02_abe_tra_sub_0p_11N	AM049741	14.11	7.30	2.59	ABCu	precerebellin-like protein
int_oss_T5H03_osl_sal_std_5p_11S	CK885905	13.56	3.62	3.37	ABCu	Salmo salar clone Rsa459 microsatellite sequence
hrt_opk_04G23_osl_sgp_std_5p_11C	CK883399	13.01	3.72	3.71	ABCu	similar to mucoepidermoid carcinoma translocated 1
liv_dis_D5D03_abe_tra_sub_0p_11N	AM049783	12.53	4.78	3.19	ABCu	similar to catechol-O-methyltransferase domain containing 1
mus_opk_08K02_osl_sgp_std_5p_11S	CK900277	11.92	4.74	2.18	ABCu	(Q5RI56) Optineurin
swi_rpk_74C11_osl_sgp_std_5p_11C	CK896571	11.81	9.69	2.11	ABCu	Salmo salar aryl hydrocarbon receptor 2 gamma (AhR2g) mRNA complete cds
liv_lrr_03G12_gal_sal_std_5p_11C	No Acc	10.94	2.89	3.02	ABCu	hypothetical protein XP_677792 isoform 1
liv_dis_D4E08_abe_tra_sub_0p_11N	AM049716	10.56	3.88	2.69	ABCu	(Q40313) Caffeoyl-CoA O-methyltransferase (EC 2.1.1.104)
int_oss_T4N10_osl_sal_std_5p_11C	CK885188	10.42	8.59	2.11	ABCu	(P55145) ARMET protein precursor (Arginine-rich protein)
tes_tsr_02D08_gal_sal_std_5p_11C	BM414146	9.62	2.91	2.87	ABCu	(Q99K41) EMILIN-1 precursor (Elastin microfibril interface-located protein 1)
eye_opk_20I02_osl_sgp_std_5p_11S	CO471610	7.56	2.97	2.86	ABCu	(P27797) Calreticulin precursor (CRP55) (Calregulin)
ova_opk_10F02_osl_sgp_std_5p_11C	CK891351	7.52	3.57	2.50	ABCu	(P22123) Ras-related protein O-Krev
hkd_opk_02C06_osl_sgp_std_5p_11S	CK880842	7.22	7.18	2.01	ABCu	Oncorhynchus mykiss calreticulin (CRT) mRNA complete cds
ova_opk_10C23_osl_sgp_std_5p_11S	CK891239	6.86	2.61	2.47	ABCu	(P15253) Calreticulin precursor (CRP55) (Calregulin)
bra_snb_01G06_sti_tra_nrc_5p_11S	EG648223	6.33	3.47	2.31	ABCu	Gallus gallus BAC clone TAM31-25K21 from chromosome z complete sequence
swi_rpk_74G15_osl_sgp_std_5p_11S	CK895552	4.42	2.22	2.73	ABCu	chemotaxin
int_oss_THJ05_osl_sal_std_5p_11C	CN181296	4.38	2.61	2.39	ABCu	(P15253) Calreticulin precursor (CRP55) (Calregulin)
liv_dis_D4G10_abe_tra_sub_0p_11N	AM049729	4.24	2.18	2.57	ABCu	precerebellin-like protein
swi_rpk_74D07_osl_sgp_std_5p_11S	CK896819	4.23	2.82	2.20	ABCu	(P27797) Calreticulin precursor (CRP55) (Calregulin)
ova_oyr_02D04_gal_sal_std_5p_11S	BM414061	4.13	2.09	4.07	ABCu	(P97571) Calpain-1 catalytic subunit (EC 3.4.22.52)
tes_opk_14J12_osl_sgp_std_5p_11C	CK897468	3.50	2.50	2.04	ABCu	No Hit
liv_opk_12K22_osl_sgp_std_5p_11S	CK888317	3.31	2.25	2.04	ABCu	(Q99PL5) Ribosome-binding protein 1 (Ribosome receptor protein) (mRRp)

bra_bfo_02D04_fou_sal_nrc_5p_11S	DW588272	2.36	Cu	(P37268) Squalene synthetase (EC 2.5.1.21)
bra_bfo_04B02_fou_sal_nrc_3p_11C	DW588505	2.11	Cu	(P07153) Dolichyl-diphosphooligosaccharide--protein glycosyltransferase 67 kDa subunit (EC 2.4.1.119)
bra_bfo_15H04_fou_sal_nrp_5p_11M	DW590154	2.31	Cu	hypothetical protein LOC327040
bra_bfo_15H06_fou_sal_nrp_5p_11M	DW590158	2.73	Cu	(P46410) Glutamine synthetase (EC 6.3.1.2) (Glutamate--ammonia ligase) (GS)
bra_snb_09B09_osl_tra_nrc_5p_11S	EG647637	2.77	Cu	squalene monooxygenase
gil_oss_52N23_osl_sal_std_5p_11C	CK879377	3.05	Cu	(Q9N2D5) Glyceraldehyde-3-phosphate dehydrogenase (EC 1.2.1.12) (GAPDH)
gil_oss_GHB16_osl_sal_std_5p_11C	CN181053	2.30	Cu	(P08110) Endoplasmin precursor (Heat shock 108 kDa protein) (Transferrin-binding protein)
gil_rpk_76A21_osl_sgp_std_5p_11C	CK878328	2.06	Cu	(P08294) Extracellular superoxide dismutase [Cu-Zn] precursor (EC 1.15.1.1)
hkd_opk_02O22_osl_sgp_std_5p_11C	CK881623	2.09	Cu	(O42563) Cytochrome P450 3A27 (EC 1.14.14.1) (CYPIIIA27)
hrt_opk_06E02_osl_sgp_std_5p_11C	CK874573	2.68	Cu	(P26632) Early growth response protein 1 (EGR-1) (Krox24)
hrt_opk_06O21_osl_sgp_std_5p_11C	CK874989	3.13	Cu	(O88822) Lathosterol oxidase (EC 1.3.3.2) (Lathosterol 5-desaturase)
int_oss_T4G02_osl_sal_std_5p_11S	CK884617	2.07	Cu	(Q9WTL2) Ras-related protein Rab-25
int_oss_T4K02_osl_sal_std_5p_11C	CK884637	2.10	Cu	(O57521) Heat shock protein HSP 90-beta
int_rpk_78H05_osl_sgp_std_5p_11S	CK886129	2.91	Cu	(P51469) Glyceraldehyde-3-phosphate dehydrogenase (EC 1.2.1.12) (GAPDH)
int_rpk_78K19_osl_sgp_std_5p_11C	CK886399	2.73	Cu	hypothetical protein LOC768126
liv_ali_01E09_abe_tra_sub_0p_11S	AM402497	2.74	Cu	(P23228) Hydroxymethylglutaryl-CoA synthase cytoplasmic (EC 2.3.3.10)
liv_ali_02D06_abe_tra_sub_0p_11S	AM412019	2.06	Cu	No Hit
liv_ali_02E10_abe_tra_sub_0p_11C	AM402574	2.13	Cu	(Q866Y3) N-acetylmuramoyl-L-alanine amidase precursor (EC 3.5.1.28)
liv_ali_02G01_abe_tra_sub_0p_11C	AM402595	5.33	Cu	C-type MBL-2 protein
liv_ali_02H02_abe_tra_sub_0p_11C	AM402609	2.92	Cu	Type-4 ice-structuring protein precursor (Antifreeze protein type IV)
liv_ali_03E06_abe_tra_sub_0p_11C	AM402665	2.63	Cu	alanine-glyoxylate aminotransferase
liv_ali_03F06_abe_tra_sub_0p_11S	AM412024	2.09	Cu	No Hit
liv_ali_06G02_abe_tra_sub_0p_11S	AM402930	2.05	Cu	Bos taurus mono(ADP-ribosyl)transferase (ART1) mRNA
liv_ali_06G07_abe_tra_sub_0p_11C	AM402934	2.87	Cu	ovary-specific C1q-like factor
liv_dis_D3E09_abe_tra_sub_0p_11N	AM049641	3.93	Cu	C-type MBL-2 protein
liv_dis_D4B06_abe_tra_sub_0p_11N	AM049686	2.04	Cu	(Q9WUW3) Complement factor I precursor (EC 3.4.21.45) (C3B/C4B inactivator)
liv_dis_D5B03_abe_tra_sub_0p_11N	AM049762	3.83	Cu	C-type MBL-2 protein
liv_dis_D5D11_abe_tra_sub_0p_11N	AM049780	3.90	Cu	C-type MBL-2 protein
liv_lrr_01C04_gal_sal_std_5p_11C	No Acc	2.91	Cu	(Q02988) Lectin precursor
liv_opk_12D08_osl_sgp_std_5p_11C	CK888627	2.10	Cu	CGOAVACA16Coregonus artedii DNA dispersed repeat AvaIII clone Ava(CAr)-1507
liv_opk_12F09_osl_sgp_std_5p_11C	CK888938	2.12	Cu	glutathione peroxidase 3
liv_opk_12F15_osl_sgp_std_5p_11C	CK888977	2.00	Cu	(P16930) Fumarylacetoacetase (EC 3.7.1.2) (Fumarylacetoacetate hydrolase)
liv_stb_J4D11_sti_tra_sub_0p_11S	AM397499	3.17	Cu	(P80961) Antifreeze protein LS-12 precursor
mus_snm_07E07_osl_tra_nrc_5p_11C	EG648543	3.81	Cu	(Q20799) Probable calcium-binding mitochondrial carrier F55A11.4
mus_snm_07H11_osl_tra_nrc_5p_11C	EG648581	2.63	Cu	(P82264) GLutamate dehydrogenase (EC 1.4.1.3) (GDH)
ova_oyr_04D02_gal_sal_std_5p_11S	BM414075	4.06	Cu	(Q8BTW8) CDK5 regulatory subunit associated protein 1

ova_oyr_04G03_gal_sal_std_5p_11S	BM414489		2.02	Cu	(Q29437) Copper amine oxidase liver isozyme precursor (EC 1.4.3.6))
ova_oyr_06B09_gal_sal_std_5p_11C	BM414425		2.69	Cu	unnamed protein product
ova_oyr_08A01_gal_sal_std_5p_11C	BM414030		5.24	Cu	(Q9Z2N8) Actin-like protein 6A (53 kDa BRG1-associated factor A)
ova_oyr_08A05_gal_sal_std_5p_11S	BM414519		2.85	Cu	similar to MGC86501 protein isoform 1
ova_oyr_08H05_gal_sal_std_5p_11S	BM414096		2.12	Cu	(Q96PM5) RING finger and CHY zinc finger domain containing protein 1
swi_rpk_74E16_osl_sgp_std_5p_11C	CK896964		6.34	Cu	C1q-like adipose specific protein
swi_rpk_74F07_osl_sgp_std_5p_11C	CK896920		9.77	Cu	(Q9ES30) Complement C1q tumor necrosis factor-related protein 3 precursor
swi_rpk_74J03_osl_sgp_std_5p_11S	CK896189		2.59	Cu	No Hit
swi_rpk_74J22_osl_sgp_std_5p_11C	CK896123		3.91	Cu	similar to Elongation of very long chain fatty acids protein 2
swi_rpk_74K12_osl_sgp_std_5p_11C	CK895973		2.74	Cu	betaine-homocysteine methyltransferase
swi_rpk_74M05_osl_sgp_std_5p_11S	CK895001		3.68	Cu	isocitrate dehydrogenase 2 (NADP+) mitochondrial
tes_opk_14D02_osl_sgp_std_5p_11S	CK899111		2.08	Cu	Gldc-prov protein
tes_opk_14E17_osl_sgp_std_5p_11S	CK899203		2.26	Cu	(P29788) Vitronectin precursor (Serum spreading factor) (S-protein)
Down regulated genes					
ova_opk_11N23_osl_sgp_std_5p_11S	CK889658	-4.56		Ad	No Hit
mus_mfo_13G07_fou_sal_nrp_5p_11S	DW591960	-3.36		Ad	(Q5BJX0) Protein C9orf32 homolog
kid_sts_15F06_sti_sal_std_5p_22S	AJ424783	-2.87		Ad	Salmo salar caspase 3B gene complete cds
kid_opk_01L24_osl_sgp_std_5p_11C	CK887104	-2.70		Ad	No Hit
kid_aki_07E09_abe_tra_sub_0p_11C	AM042513	-2.64		Ad	No Hit
spl_sts_13G09_sti_sal_std_5p_22C	AJ425502	-2.58		Ad	HMOX_FUGRUHeme oxygenase (HO)
eye_opk_18P23_osl_sgp_std_5p_11S	CO470055	-2.43		Ad	No Hit
int_oss_T4G19_osl_sal_std_5p_11C	CK885081	-2.40		Ad	PREDICTED: sestrin 1 isoform 3
kid_sts_22D08_sti_sal_sub_0p_11C	AJ424936	-2.40		Ad	(Q811M5) Complement component C6 precursor
can_ELO_S1B07_sti_tra_can_0p_11N	No Acc	-2.28		Ad	At. sal. elongase_orf_950bpContig Blast SW
kid_opk_01O22_osl_sgp_std_5p_11C	CK886639	-2.26		Ad	PREDICTED: Canis familiaris similar to tsukushi (LOC485178) mRNA
gil_agi_06A02_abe_tra_sub_0p_11S	AM412188	-2.24		Ad	(P54216) Fructose-bisphosphate aldolase 1 (EC 4.1.2.13) (Aldolase CE-1) (CE1)
ova_oyr_06H05_gal_sal_std_5p_11S	BM414052	-2.18		Ad	(Q5RDH5) 5'-AMP-activated protein kinase catalytic alpha-1 chain (EC 2.7.1.-) (AMPK alpha-1 chain)
tes_opk_14E07_osl_sgp_std_5p_11S	CK899176	-2.13		Ad	PREDICTED: similar to PERP TP53 apoptosis effector
hkd_opk_04B20_osl_sgp_std_5p_11C	CK880168	-2.12		Ad	Urobatis jamaicensis internal transcribed spacer 2 and 28S ribosomal RNA gene partial sequence
bra_snb_13F05_osl_tra_nrc_5p_11C	No Acc	-2.11		Ad	hypothetical 18K protein - goldfish mitochondrion
gil_cgi_E2E03_car_tra_sub_0p_11N	No Acc	-2.10		Ad	No Hit
tes_opk_15G09_osl_sgp_std_5p_11C	CK897241	-2.07		Ad	neuroblastoma suppression of tumorigenicity 1
int_oss_T4P21_osl_sal_std_5p_11C	CK884750	-2.07		Ad	(Q99MI7) Ubiquitin-activating enzyme E1C (Nedd8-activating enzyme E1C)
spl_sts_13G09_sti_sal_std_5p_12C	AJ425502	-2.03		Ad	HMOX_FUGRUHeme oxygenase (HO)
swi_rpk_74I03_osl_sgp_std_5p_11S	CK895643	-2.02		Ad	No Hit

tes_tsr_05D09_gal_sal_std_5p_11S	BM414139	-2.02		Ad	B-G-like protein
mus_mfo_08F04_fou_sal_nrp_5p_11C	DW591222	-2.02		Ad	No Hit
liv_ali_05F02_abe_tra_sub_0p_11S	AM402843	-2.01		Ad	(P04114) Apolipoprotein B-100 precursor (Apo B-100)
gil_rpk_75B01_osl_sgp_std_5p_11C	CK879639	-2.00		Ad	(Q501J6) Probable ATP-dependent helicase DDX17 (EC 3.6.1.-) (DEAD-box protein 17)
ova_oyr_08A01_gal_sal_std_5p_11C	BM414030		-9.54	Bd	(Q9Z2N8) Actin-like protein 6A (53 kDa BRG1-associated factor A)
swi_rpk_74J11_osl_sgp_std_5p_11C	CK896114		-4.46	Bd	AF055439Oncorhynchus kisutch microsatellite OKi14 DNA
ova_opk_09N23_osl_sgp_std_5p_11C	CK890982		-4.12	Bd	(P49638) Alpha-tocopherol transfer protein (Alpha-TTP)
spl_sts_17G04_sti_sal_std_5p_11C	AJ425609		-4.02	Bd	(P04431) Ig kappa chain V-I region Walker precursor
int_oss_T4F12_osl_sal_std_5p_11S	CK885151		-3.88	Bd	suppressor of cytokine signaling 1-like protein
swi_rpk_74J22_osl_sgp_std_5p_11C	CK896123		-3.88	Bd	PREDICTED: similar to Elongation of very long chain fatty acids protein 2
swi_rpk_74L09_osl_sgp_std_5p_11C	CK896176		-3.83	Bd	No Hit
int_oss_T4K02_osl_sal_std_5p_11C	CK884637		-3.82	Bd	(O57521) Heat shock protein HSP 90-beta
liv_ali_05D10_abe_tra_sub_0p_11S	AM402821		-3.79	Bd	(O70127) Bile salt export pump (ATP-binding cassette sub-family B member 11)
liv_ali_05D12_abe_tra_sub_0p_11C	AM402823		-3.77	Bd	(P21643) Tryptophan 2 3-dioxygenase (EC 1.13.11.11)
liv_ali_02G01_abe_tra_sub_0p_11C	AM402595		-3.76	Bd	C-type MBL-2 protein
liv_opk_12K10_osl_sgp_std_5p_11C	CK888239		-3.66	Bd	No Hit
liv_opk_12I20_osl_sgp_std_5p_11C	CK889570		-3.65	Bd	No Hit
liv_opk_12G17_osl_sgp_std_5p_11S	CK889175		-3.55	Bd	No Hit
kid_opk_01L17_osl_sgp_std_5p_11C	CK887085		-3.50	Bd	unnamed protein product
hrt_opk_08H03_osl_sgp_std_5p_11C	CK899703		-3.43	Bd	hypothetical protein LOC553776
mus_mfo_03E02_fou_sal_nrp_5p_11M	DW590543		-3.41	Bd	(Q9WUK2) Eukaryotic translation initiation factor 4H (eIF-4H)
liv_ali_05B07_abe_tra_sub_0p_11C	AM402805		-3.40	Bd	PREDICTED: similar to IGFALS
bra_bfo_07B02_fou_sal_nrc_5p_11S	DW588937		-3.33	Bd	(Q9WUR2) Peroxisomal 3 2-trans-enoyl-CoA isomerase (EC 5.3.3.8)
liv_opk_12H07_osl_sgp_std_5p_11S	CK889304		-3.33	Bd	(O95685) Protein phosphatase 1 regulatory subunit 3D
mus_mfo_14A04_fou_sal_nrp_5p_11M	DW591979		-3.25	Bd	PREDICTED: Canis familiaris hypothetical LOC482885 (LOC482885)
swi_rpk_74H13_osl_sgp_std_5p_11S	CK895853		-3.25	Bd	hypothetical protein LOC389936
ova_oyr_08H09_gal_sal_std_5p_11S	BM414382		-3.24	Bd	(Q9UJW8) Zinc finger protein 180 (HHZ168)
swi_rpk_74K12_osl_sgp_std_5p_11C	CK895973		-3.11	Bd	betaine-homocysteine methyltransferase
mus_snm_06H10_osl_tra_nrc_5p_11C	EG649369		-3.11	Bd	hypothetical protein
kid_sts_15A02_sti_sal_std_5p_11C	AJ424722		-3.10	Bd	No Hit
gil_rpk_76A11_osl_sgp_std_5p_11C	CK878535		-3.07	Bd	Danio rerio solute carrier family 38 member 4 (slc38a4) mRNA
int_oss_T4G02_osl_sal_std_5p_11S	CK884617		-3.03	Bd	(Q9WTL2) Ras-related protein Rab-25
liv_ali_01B01_abe_tra_sub_0p_11C	AM402454		-3.03	Bd	(Q9PT92) Catalase (EC 1.11.1.6)
int_oss_T5L06_osl_sal_std_5p_11C	CK886009		-3.02	Bd	(O75956) DOC-1-related protein (DOC-1R)
hkd_opk_03J10_osl_sgp_std_5p_11C	CK880513		-3.02	Bd	No Hit
spl_sts_17G06_sti_sal_std_5p_22C	AJ425611		-2.99	Bd	PREDICTED: hypothetical protein XP_697594

tes_tsr_07A09_gal_sal_std_5p_11S	BM414190	-2.98	Bd	Oncorhynchus mykiss SLC26A1-like protein mRNA complete cds
swi_rpk_74D11_osl_sgp_std_5p_11S	CK896827	-2.95	Bd	(Q91Z53) Glyoxylate reductase/hydroxypyruvate reductase (EC 1.1.1.79)
int_oss_T4C13_osl_sal_std_5p_11S	CK885058	-2.94	Bd	(P11884) Aldehyde dehydrogenase mitochondrial precursor (EC 1.2.1.3) (ALDH class 2)
ova_oyr_08D12_gal_sal_std_5p_12C	BM414031	-2.92	Bd	(P10949) Ras-related protein Rab-3C (SMG P25C)
gil_oss_G5C14_osl_sal_std_5p_11C	CK878932	-2.90	Bd	(P03974) Transitional endoplasmic reticulum ATPase (TER ATPase)
int_oss_THC11_osl_sal_std_5p_11S	CN181332	-2.90	Bd	Salmo salar caspase 7 gene complete cds
tes_opk_14D02_osl_sgp_std_5p_11S	CK899111	-2.85	Bd	Gldc-prov protein
liv_ali_04C01_abe_tra_sub_0p_11S	AM412026	-2.84	Bd	unnamed protein product
liv_ali_05H09_abe_tra_sub_0p_11S	AM402867	-2.81	Bd	(P07589) Fibronectin (FN)
swi_rpk_74I18_osl_sgp_std_5p_11S	CK895661	-2.81	Bd	(Q03156) Serum albumin 2 precursor
liv_ali_03C09_abe_tra_sub_0p_11S	AM402646	-2.80	Bd	(O57523) Apolipoprotein A-I-1 precursor (Apo-AI-1) (ApoA-I-1)
mus_snm_06F02_osl_tra_nrc_5p_11C	EG649036	-2.76	Bd	(P33436) 72 kDa type IV collagenase precursor (EC 3.4.24.24)
int_oss_T4K05_osl_sal_std_5p_11C	CK885096	-2.75	Bd	(P20720) UDP-glucuronosyltransferase 1-2 precursor microsomal (EC 2.4.1.17)
mus_snm_01H03_sti_tra_nrc_5p_11C	EG649260	-2.74	Bd	(P14527) Hemoglobin alpha-4 subunit
gil_agi_01F07_abe_tra_sub_0p_11S	AM041470	-2.73	Bd	Oncorhynchus mykiss SYPG1 (SYPG1) PHF1 (PHF1) and RGL2 (RGL2) genes complete cds;
kid_sts_22B08_sti_sal_sub_0p_11C	AJ424903	-2.73	Bd	(P25782) Digestive cysteine proteinase 2 precursor (EC 3.4.22.-)
spl_sts_04B04_sti_sal_std_5p_11S	AJ425084	-2.72	Bd	No Hit
swi_rpk_74G17_osl_sgp_std_5p_11C	CK895558	-2.72	Bd	PREDICTED: similar to Prostaglandin-H2 D-isomerase precursor
kid_cki_A1E02_car_tra_sub_0p_11C	EG355353	-2.72	Bd	No Hit
mus_snm_07H11_osl_tra_nrc_5p_11C	EG648581	-2.72	Bd	(P82264) GLutamate dehydrogenase (EC 1.4.1.3) (GDH)
liv_ali_05C12_abe_tra_sub_0p_11C	AM402811	-2.69	Bd	(P42357) Histidine ammonia-lyase (EC 4.3.1.3) (Histidase)
int_oss_T5G04_osl_sal_std_5p_11C	CK885817	-2.69	Bd	hypothetical protein LOC563356
ski_opk_09C13_osl_sgp_std_5p_11S	CK892424	-2.69	Bd	(P56533) Betaine aldehyde dehydrogenase (EC 1.2.1.8) (BADH)
int_rpk_78K19_osl_sgp_std_5p_11C	CK886399	-2.68	Bd	hypothetical protein LOC768126
bra_bfo_16H05_fou_sal_nrp_5p_11M	DW590176	-2.68	Bd	No Hit
liv_stb_K4D02_sti_tra_sub_0p_11C	AM397504	-2.68	Bd	(P35858) Insulin-like growth factor-binding protein complex acid labile chain precursor (ALS)
swi_rpk_74D06_osl_sgp_std_5p_11C	CK896685	-2.67	Bd	Uncharacterized protein C6orf58 homolog precursor
liv_ali_05G02_abe_tra_sub_0p_11S	AM402853	-2.67	Bd	(P53657) Pyruvate kinase isozymes R/L (EC 2.7.1.40)
liv_ali_04D09_abe_tra_sub_0p_11C	AM402738	-2.67	Bd	AF321816Oncorhynchus mykiss hepatic glucose transporter GLUT2 mRNA complete cds
tes_tsr_04G07_gal_sal_std_5p_11C	BM413861	-2.66	Bd	(Q7ZVB1) Charged multivesicular body protein 1b (Chromatin modifying protein 1b)
bra_bfo_08H11_fou_sal_nrc_5p_11C	DW589243	-2.66	Bd	(Q92947) Glutaryl-CoA dehydrogenase mitochondrial precursor (EC 1.3.99.7)
int_rpk_78A14_osl_sgp_std_5p_11S	CK885482	-2.65	Bd	No Hit
bra_snb_02F08_sti_tra_nrc_5p_11C	EG648298	-2.65	Bd	(P47954) Glutathione S-transferase P (EC 2.5.1.18) (GST class-pi)
kid_sts_14A06_sti_sal_std_5p_12C	AJ424632	-2.65	Bd	(P20135) Glutathione S-transferase 1 (EC 2.5.1.18)
mus_mfo_07D01_fou_sal_nrp_5p_11M	DW591050	-2.65	Bd	No Hit
swi_rpk_74D21_osl_sgp_std_5p_11C	CK896723	-2.64	Bd	(O43826) Glucose-6-phosphate translocase

liv_ali_03F06_abe_tra_sub_0p_11S	AM412024	-2.62	Bd	No Hit
hrt_opk_08L19_osl_sgp_std_5p_11C	CK899381	-2.62	Bd	(Q15149) Plectin 1 (PLTN) (PCN) (Hemidesmosomal protein 1) (HD1)
liv_ali_03E06_abe_tra_sub_0p_11C	AM402665	-2.60	Bd	alanine-glyoxylate aminotransferase
bra_snb_06H08_osl_tra_nrc_5p_11C	EG648264	-2.58	Bd	(P62762) Visinin-like protein 1 (VILIP) (Neural visinin-like protein 1)
liv_stb_B4E11_sti_tra_sub_0p_11C	AM397486	-2.57	Bd	(Q92088) Cytochrome P450 2M1 (EC 1.14.14.1)
mus_snm_06C03_osl_tra_nrc_5p_11C	EG648671	-2.56	Bd	unnamed protein product
liv_ali_04C06_abe_tra_sub_0p_11C	AM402725	-2.56	Bd	(P28665) Murinoglobulin-1 precursor (MuG1)
liv_ali_04A04_abe_tra_sub_0p_11S	AM402704	-2.55	Bd	Oncorhynchus mykiss putative serum albumin-related protein mRNA partial cds
mus_snm_12H02_osl_tra_nrc_5p_11S	EG649006	-2.55	Bd	hypothetical protein LOC768158
liv_opk_12D06_osl_sgp_std_5p_11S	CK888616	-2.55	Bd	(Q90592) Solute carrier family 2 facilitated glucose transporter member 2
ova_oyr_02C02_gal_sal_std_5p_11C	BM414462	-2.55	Bd	hypothetical protein LOC751685
bra_bfo_07H02_fou_sal_nrc_5p_11M	DW589070	-2.54	Bd	No Hit
liv_ali_06D04_abe_tra_sub_0p_11C	AM402903	-2.53	Bd	(Q61704) Inter-alpha-trypsin inhibitor heavy chain H3 precursor
liv_ali_02B09_abe_tra_sub_0p_11C	AM402549	-2.53	Bd	(Q61703) Inter-alpha-trypsin inhibitor heavy chain H2 precursor
eye_rpk_73L18_osl_sgp_std_5p_11S	CO472531	-2.53	Bd	(Q64442) Sorbitol dehydrogenase (EC 1.1.1.14) (
int_oss_T4L18_osl_sal_std_5p_11C	CK885181	-2.52	Bd	No Hit
kid_sts_10E10_sti_sal_std_5p_11C	AJ424553	-2.51	Bd	proprotein convertase subtilisin/kexin type5b
spl_opk_15J02_osl_sgp_std_5p_11S	CK894143	-2.50	Bd	(P31643) Sodium- and chloride-dependent taurine transporter
eye_rpk_73I22_osl_sgp_std_5p_11C	CO472476	-2.48	Bd	lipoprotein lipase
kid_sts_02C07_sti_sal_std_5p_11S	AJ424194	-2.48	Bd	No Hit
hkd_opk_02O22_osl_sgp_std_5p_11C	CK881623	-2.46	Bd	(O42563) Cytochrome P450 3A27 (EC 1.14.14.1) (CYPIIIA27)
hrt_opk_04O04_osl_sgp_std_5p_11C	CK883910	-2.46	Bd	Acyl-Coenzyme A dehydrogenase long chain
spl_sts_19H10_sti_sal_std_5p_11C	AJ425791	-2.46	Bd	(O35250) Exocyst complex component 7
mus_mfo_1aB10_fou_sal_nrp_5p_11M	DW590197	-2.46	Bd	(P02142) Hemoglobin beta-1 subunit
liv_opk_12D08_osl_sgp_std_5p_11C	CK888627	-2.45	Bd	CGOAVACA16Coregonus artedii DNA dispersed repeat AvaIII clone Ava(CAr)-1507
spl_opk_15N02_osl_sgp_std_5p_11C	CK894451	-2.45	Bd	(P30712) Glutathione S-transferase theta 2 (EC 2.5.1.18) (GST class-theta 2)
liv_lrr_08B08_gal_sal_std_5p_11S	BI468166	-2.45	Bd	ONHSOXP1Oncorhynchus mykiss mRNA for SoxP1 complete cds
bra_bfo_09C03_fou_sal_nrc_5p_11S	DW589277	-2.45	Bd	(Q15262) Receptor-type tyrosine-protein phosphatase kappa precursor (EC 3.1.3.48)
liv_stb_B4G01_sti_tra_sub_0p_11S	AM397491	-2.44	Bd	(P01023) Alpha-2-macroglobulin precursor (Alpha-2-M)
ova_oyr_04G07_gal_sal_std_5p_12C	No Acc	-2.44	Bd	(Q9BWX1) PHD finger protein 7 (Protein NYD-SP6)
kid_cki_A4H09_car_tra_sub_0p_11C	EG355339	-2.43	Bd	(P30568) Glutathione S-transferase A (EC 2.5.1.18)
swi_rpk_74D18_osl_sgp_std_5p_11C	CK896717	-2.43	Bd	No Hit
spl_sts_12A03_sti_sal_std_5p_11C	AJ425337	-2.43	Bd	AY872256S1Oncorhynchus mykiss IgH.A locus partial sequence
liv_ali_03H09_abe_tra_sub_0p_11C	AM402697	-2.42	Bd	(P14046) Alpha-1-inhibitor 3 precursor (Alpha-1-inhibitor III)
spl_sts_03B08_sti_sal_std_5p_11C	AJ424994	-2.42	Bd	(P0C033) Ubiquitin-like protein 1
kid_cki_A3H02_car_tra_sub_0p_11C	EG355290	-2.42	Bd	CNS0EPR6Tetraodon nigroviridis full-length cDNA
liv_opk_12F21_osl_sgp_std_5p_11S	CK889023	-2.42	Bd	CNS0G4CRTetraodon nigroviridis full-length cDNA

int_oss_T5C13_osl_sal_std_5p_11S	CK885711	-2.41	Bd	cytochrome P450
mus_mfo_1aA07_fou_sal_nrp_5p_11C	DW590182	-2.41	Bd	hypothetical protein LOC553639
ova_opk_10A17_osl_sgp_std_5p_11C	CK891125	-2.41	Bd	No Hit
kid_sts_01F09_sti_sal_std_5p_11S	AJ424144	-2.41	Bd	PREDICTED: similar to MGC84339 protein isoform 1
bra_bfo_14G05_fou_sal_nrp_5p_11S	No Acc	-2.40	Bd	PREDICTED: Mus musculus membrane-associated ring finger (C3HC4) 9 (March9) mRNA
liv_ali_04F03_abe_tra_sub_0p_11C	AM402756	-2.40	Bd	unnamed protein product
liv_opk_12L17_osl_sgp_std_5p_11S	CK888473	-2.40	Bd	(P17475) Alpha-1-antiproteinase precursor (Alpha-1-antitrypsin)
bra_bfo_03F11_fou_sal_nrc_5p_11C	DW588445	-2.40	Bd	(Q9DEX7) Delta-5/delta-6 fatty acid desaturase (EC 1.14.19.-)
liv_ali_04H06_abe_tra_sub_0p_11C	AM402781	-2.40	Bd	(Q8HXX8) Glutaryl-CoA dehydrogenase mitochondrial precursor (EC 1.3.99.7)
tes_opk_14L10_osl_sgp_std_5p_11C	CK897577	-2.39	Bd	(P24722) Creatine kinase testis isozyme (EC 2.7.3.2)
mus_mfo_11D11_fou_sal_nrp_5p_11M	DW591647	-2.38	Bd	electron-transfer-flavoprotein beta polypeptide
liv_opk_12I22_osl_sgp_std_5p_11C	CK889576	-2.38	Bd	hypothetical protein LOC492516
gil_agi_01G06_abe_tra_sub_0p_11C	AM041479	-2.38	Bd	(P20373) L-lactate dehydrogenase B chain (EC 1.1.1.27)
liv_opk_12L23_osl_sgp_std_5p_11S	CK888535	-2.37	Bd	(Q04499) Proline oxidase mitochondrial precursor (EC 1.5.3.-)
ova_oyr_08F10_gal_sal_std_5p_11C	No Acc	-2.36	Bd	hypothetical protein LOC558615
liv_ali_02C10_abe_tra_sub_0p_11C	AM402551	-2.35	Bd	(Q00796) Sorbitol dehydrogenase (EC 1.1.1.14) (L-iditol 2-dehydrogenase)
mus_snm_08F03_osl_tra_nrc_5p_11C	EG648643	-2.35	Bd	phytanoyl-CoA hydroxylase
ova_oyr_08H08_gal_sal_std_5p_11C	No Acc	-2.35	Bd	(P46926) Glucosamine-6-phosphate isomerase (EC 3.5.99.6)
bra_bfo_01C04_fou_sal_nrc_5p_11S	DW588077	-2.34	Bd	(O60729) Dual specificity protein phosphatase CDC14B (EC 3.1.3.48)
liv_opk_12H01_osl_sgp_std_5p_11C	CK889250	-2.34	Bd	(Q92090) Cytochrome P450 2K1 (EC 1.14.14.1) (CYPIIK1) (P450 LMC2)
kid_opk_01E13_osl_sgp_std_5p_11C	CK887681	-2.33	Bd	(P21213) Histidine ammonia-lyase (EC 4.3.1.3)
int_rpk_78K01_osl_sgp_std_5p_11S	CK886455	-2.33	Bd	(Q9DBM2) Peroxisomal bifunctional enzyme (PBE) (PBFE)
bra_opk_07F12_osl_sgp_std_5p_11S	CK875274	-2.33	Bd	(P15650) Acyl-CoA dehydrogenase long-chain specific mitochondrial precursor (EC 1.3.99.13)
ova_oyr_08D12_gal_sal_std_5p_22C	BM414031	-2.33	Bd	(P10949) Ras-related protein Rab-3C (SMG P25C)
tes_opk_15D09_osl_sgp_std_5p_11C	CK897993	-2.33	Bd	putative serum albumin-related protein
mus_opk_08L20_osl_sgp_std_5p_11S	CK899383	-2.32	Bd	(Q71U34) Heat shock cognate 71 kDa protein
eye_opk_19D15_osl_sgp_std_5p_11C	CO470274	-2.32	Bd	hypothetical protein LOC393575
liv_ali_06E03_abe_tra_sub_0p_11S	AM402913	-2.32	Bd	putative 14 kDa apolipoprotein
ova_oyr_05G12_gal_sal_std_5p_11S	BM414504	-2.32	Bd	syndecan 2
mus_snm_03H06_sti_tra_nrc_5p_11C	EG649400	-2.31	Bd	(O88797) Disabled homolog 2 (DOC-2) (Mitogen-responsive phosphoprotein) (C9)
bra_snb_11D02_osl_tra_nrc_5p_11C	EG647833	-2.31	Bd	(Q92522) Histone H1x
hrt_opk_05O20_osl_sgp_std_5p_11C	CK874353	-2.31	Bd	No Hit
liv_ali_05F07_abe_tra_sub_0p_11S	AM412029	-2.31	Bd	(P20740) Ovostatin precursor (Ovomacroglobulin)
swi_rpk_74L03_osl_sgp_std_5p_11C	CK896260	-2.31	Bd	No Hit
bra_snb_01E02_sti_tra_nrc_5p_11S	EG648195	-2.30	Bd	Oncorhynchus mykiss putative serum albumin-related protein mRNA partial cds
int_oss_T4L02_osl_sal_std_5p_11C	CK885173	-2.29	Bd	(Q46891) Hypothetical protein ygbM

kid_sts_08B02_sti_sal_std_5p_11C	AJ424326	-2.29	Bd	(P24298) Alanine aminotransferase (EC 2.6.1.2)
liv_ali_02H08_abe_tra_sub_0p_11S	AM402615	-2.27	Bd	No Hit
liv_opk_12K03_osl_sgp_std_5p_11C	CK888164	-2.27	Bd	(P80961) Antifreeze protein LS-12 precursor
hrt_opk_04O08_osl_sgp_std_5p_11C	CK883919	-2.27	Bd	(P13913) Arylamine N-acetyltransferase pineal gland isozyme NAT-10 (EC 2.3.1.5)
liv_stb_B4A10_sti_tra_sub_0p_11S	AM397482	-2.27	Bd	hemopexin-like protein
int_rpk_78I13_osl_sgp_std_5p_11C	CK886350	-2.27	Bd	(P14920) D-amino-acid oxidase (EC 1.4.3.3)
tes_tsr_03B04_gal_sal_std_5p_11S	BM414242	-2.26	Bd	No Hit
bra_bfo_09D07_fou_sal_nrc_5p_11S	No Acc	-2.26	Bd	No Hit
can_CaD_S1A10_sti_tra_can_0p_12N	No Acc	-2.26	Bd	At. sal. CAROTENE DioxygenaseContig Blast SW
kid_opk_01A17_osl_sgp_std_5p_11C	CK887346	-2.25	Bd	14-3-3E1 protein
swi_rpk_74G04_osl_sgp_std_5p_11C	CK895537	-2.25	Bd	No Hit
spl_sts_13H07_sti_sal_std_5p_11C	AJ425512	-2.25	Bd	(P12762) Aldehyde dehydrogenase mitochondrial (EC 1.2.1.3)
can_D6O_S1B06_sti_tra_can_0p_11N	No Acc	-2.25	Bd	At. sal. delta6desaturase_orf_1400bpContig Blast SW
liv_opk_12J02_osl_sgp_std_5p_11C	CK887992	-2.25	Bd	PREDICTED: hypothetical protein XP_693867
can_CaD_S1C04_sti_tra_can_0p_22N	No Acc	-2.25	Bd	At. sal. CAROTENE DioxygenaseContig Blast SW
kid_sts_15E04_sti_sal_std_5p_11C	AJ424770	-2.24	Bd	No Hit
hrt_opk_07D21_osl_sgp_std_5p_11C	CK875206	-2.24	Bd	No Hit
int_oss_T6O06_osl_sal_std_5p_12S	CK884079	-2.24	Bd	novel protein containing an acyltransferase domain (zgc:91857)
liv_opk_12G05_osl_sgp_std_5p_11S	CK889074	-2.24	Bd	PREDICTED: similar to neurestin alpha
mus_snm_05C05_sti_tra_nrc_5p_11C	EG648459	-2.24	Bd	(P31335) Bifunctional purine biosynthesis protein PURH
ova_oyr_07G10_gal_sal_std_5p_22C	BM414407	-2.23	Bd	AF256963Salmo salar clone BHMS413 microsatellite sequence
liv_opk_12J17_osl_sgp_std_5p_11S	CK888094	-2.23	Bd	Danio rerio cDNA clone MGC:154069 IMAGE:8338829 complete cds
kid_opk_01L19_osl_sgp_std_5p_11C	CK887094	-2.23	Bd	(P10589) COUP transcription factor 1 (COUP-TF1) (COUP-TF I)
liv_ali_05A07_abe_tra_sub_0p_11C	AM402794	-2.22	Bd	Salmo salar clone Rsa532 microsatellite sequence
liv_opk_12I17_osl_sgp_std_5p_11C	CK889559	-2.22	Bd	(Q8R121) Protein Z-dependent protease inhibitor precursor
eye_rpk_73K02_osl_sgp_std_5p_11C	CO472502	-2.22	Bd	cytosolic malate dehydrogenase A
kid_sts_14A06_sti_sal_std_5p_22C	AJ424632	-2.22	Bd	(P20135) Glutathione S-transferase 1 (EC 2.5.1.18)
spl_sts_06B01_sti_sal_std_5p_22C	AJ425174	-2.22	Bd	(P49019) Probable G-protein coupled receptor 109B
swi_rpk_74F07_osl_sgp_std_5p_11C	CK896920	-2.21	Bd	(Q9ES30) Complement C1q tumor necrosis factor-related protein 3 precursor
ova_oyr_02F07_gal_sal_std_5p_11C	BM414416	-2.21	Bd	Danio rerio zgc:85948 (zgc:85948) mRNA
kid_sts_16B08_sti_sal_std_5p_11C	AJ424831	-2.21	Bd	simple type II keratin K8b (S2)
hrt_opk_05C22_osl_sgp_std_5p_11C	CK882801	-2.20	Bd	(Q9Y2Q3) Glutathione S-transferase kappa 1 (EC 2.5.1.18)
kid_opk_01L10_osl_sgp_std_5p_11C	CK887060	-2.20	Bd	No Hit
liv_ali_06E06_abe_tra_sub_0p_11S	AM402915	-2.20	Bd	hypothetical protein LOC447807
bra_bfo_10D02_fou_sal_nrc_5p_11M	DW589447	-2.19	Bd	transposase
bra_bfo_07B08_fou_sal_nrc_5p_11M	DW588946	-2.19	Bd	(O42259) Glyceraldehyde-3-phosphate dehydrogenase (EC 1.2.1.12)
int_oss_T4O03_osl_sal_std_5p_11C	CK885116	-2.19	Bd	(Q5M875) 17-beta hydroxysteroid dehydrogenase 13 precursor

liv_ali_06G03_abe_tra_sub_0p_11S	AM412031	-2.19	Bd	putative 14 kDa apolipoprotein
bra_snb_12A03_osl_tra_nrc_5p_11C	EG647882	-2.18	Bd	(P52756) RNA-binding protein 5 (RNA-binding motif protein 5)
bra_opk_05O24_osl_sgp_std_5p_11S	CK874360	-2.18	Bd	(P08108) Heat shock cognate 70 kDa protein (HSP70)
gil_oss_GHB16_osl_sal_std_5p_11C	CN181053	-2.18	Bd	(P08110) Endoplasmin precursor (Heat shock 108 kDa protein) (Transferrin-binding protein)
ova_opk_10C12_osl_sgp_std_5p_11S	CK891218	-2.18	Bd	thyroid hormone receptor associated protein complex component TRAP230/KIAA0192
int_rpk_76J09_osl_sgp_std_5p_11C	CK884572	-2.18	Bd	CNS0F44ATetraodon nigroviridis full-length cDNA
liv_ali_01E05_abe_tra_sub_0p_11S	AM402493	-2.17	Bd	QSCN6_CAVPOSulfhydryl oxidase 1 precursor (Quiescin Q6)
liv_ali_02H09_abe_tra_sub_0p_11S	AM412021	-2.17	Bd	(O57592) 60S ribosomal protein L7a (Surfeit locus protein 3)
liv_ali_04C11_abe_tra_sub_0p_11C	AM402719	-2.17	Bd	Oncorhynchus mykiss putative serum albumin-related protein mRNA partial cds
liv_lrr_07G09_gal_sal_std_5p_11C	BI468027	-2.17	Bd	(Q9GL24) Cathepsin L precursor (EC 3.4.22.15)
liv_opk_12K08_osl_sgp_std_5p_11C	CK888222	-2.17	Bd	(P48832) Zona pellucida sperm-binding protein 3 precursor (Zona pellucida glycoprotein ZP3)
swi_rpk_74F10_osl_sgp_std_5p_11S	CK897133	-2.16	Bd	Salmo salar zonadhesin-like gene complete cds and 3' UTR
kid_sts_08B05_sti_sal_std_5p_11C	AJ424329	-2.16	Bd	CNS0GOYZTetraodon nigroviridis full-length cDNA
kid_sts_16E07_sti_sal_std_5p_11C	AJ424864	-2.16	Bd	prothymosin alpha
bra_bfo_01E03_fou_sal_nrc_5p_11M	No Acc	-2.16	Bd	No Hit
liv_ali_02A06_abe_tra_sub_0p_11C	AM402536	-2.16	Bd	(P53760) Phosphatidylcholine-sterol acyltransferase precursor (EC 2.3.1.43)
can_D6D_S1B04_sti_tra_can_0p_22N	No Acc	-2.16	Bd	At. sal. delta6desaturase_401bpContig Blast SW
bra_bfo_12B09_fou_sal_nrc_5p_11M	DW589715	-2.16	Bd	(Q99L27) GMP reductase 2 (EC 1.7.1.7) (Guanosine 5'-monophosphate oxidoreductase 2)
bra_snb_03A10_sti_tra_nrc_5p_11C	EG648323	-2.15	Bd	(Q9H6R6) Zinc finger DHHC domain containing protein 6
tes_opk_14K01_osl_sgp_std_5p_11C	CK897507	-2.15	Bd	Zebrafish DNA sequence from clone CH211-197O6 in linkage group 2 complete sequence
liv_opk_12F05_osl_sgp_std_5p_11S	CK888927	-2.15	Bd	(P00506) Aspartate aminotransferase mitochondrial precursor (EC 2.6.1.1)
gil_oss_G5B20_osl_sal_std_5p_11C	CK879059	-2.15	Bd	PREDICTED: similar to leucine-rich repeat kinase 1
bra_bfo_02F02_fou_sal_nrc_3p_11S	DW588281	-2.14	Bd	AF135117_1heat shock protein hsp90 beta
gil_oss_GHO17_osl_sal_std_5p_11S	CN181107	-2.14	Bd	No Hit
liv_lrr_04A04_gal_sal_std_5p_11C	BI468028	-2.14	Bd	(Q864W1) Complement factor B precursor (EC 3.4.21.47) (C3/C5 convertase)
ova_oyr_08C12_gal_sal_std_5p_11S	BM414373	-2.14	Bd	PREDICTED: kinesin-like motor protein C20orf23
liv_opk_12L20_osl_sgp_std_5p_11S	CK888526	-2.14	Bd	(P22458) Vitronectin precursor (Serum spreading factor) (S-protein)
liv_opk_12H15_osl_sgp_std_5p_11C	CK889346	-2.14	Bd	(P10634) Cytochrome P450 2D2 (EC 1.14.14.1)
gil_rpk_75A03_osl_sgp_std_5p_11S	CK879625	-2.13	Bd	hypothetical protein LOC429380
ova_opk_11P03_osl_sgp_std_5p_11C	CK889719	-2.13	Bd	similar to hypothetical protein FLJ10839; cell-cycle and apoptosis regulatory protein 1
liv_ali_06H02_abe_tra_sub_0p_11C	AM402938	-2.13	Bd	No Hit
ova_oyr_04E02_gal_sal_std_5p_11C	BM414042	-2.13	Bd	hypothetical protein LOC393163
spl_sts_17D07_sti_sal_std_5p_22C	AJ425589	-2.13	Bd	(P50637) Peripheral-type benzodiazepine receptor
liv_ali_05H12_abe_tra_sub_0p_11S	AM402862	-2.13	Bd	No Hit
liv_ali_03D04_abe_tra_sub_0p_11S	AM402652	-2.13	Bd	(P04762) Catalase (EC 1.11.1.6)
int_oss_T4D08_osl_sal_std_5p_11S	CK885138	-2.12	Bd	Oncorhynchus mykiss putative serum albumin-related protein mRNA partial cds

tes_opk_13B03_osl_sgp_std_5p_11S	CK898277	-2.12	Bd	(P31939) Bifunctional purine biosynthesis protein PURH
hrt_opk_04H11_osl_sgp_std_5p_12S	CK883436	-2.11	Bd	(Q95JC8) Arginase-1 (EC 3.5.3.1) (Type I arginase) (Liver-type arginase)
kid_sts_01D06_sti_sal_std_5p_11C	AJ424119	-2.11	Bd	No Hit
bra_bfo_11D09_fou_sal_nrc_5p_11C	DW589613	-2.11	Bd	(Q9UMY4) Sorting nexin-12
can_D5O_S1B05_sti_tra_can_0p_11N	No Acc	-2.11	Bd	At. sal. delta5desaturase_orf_1400bpContig Blast SW
int_rpk_78M05_osl_sgp_std_5p_11C	CK884400	-2.11	Bd	(Q16719) Kynureninase (EC 3.7.1.3) (L-kynurenine hydrolase)
int_oss_T6H09_osl_sal_std_5p_11C	CK884120	-2.10	Bd	D-amino acid oxidase
spl_sts_19H09_sti_sal_std_5p_11S	AJ425790	-2.10	Bd	No Hit
liv_lrr_06F09_gal_sal_std_5p_11C	BI468110	-2.10	Bd	(P17690) Beta-2-glycoprotein I precursor (Apolipoprotein H)
kid_sts_11B10_sti_sal_std_5p_11C	AJ424608	-2.10	Bd	Zebrafish DNA sequence from clone CH211-160M12 in linkage group 6 complete sequence
liv_ali_06G02_abe_tra_sub_0p_11S	AM402930	-2.10	Bd	PREDICTED: Bos taurus mono(ADP-ribosyl)transferase (ART1) mRNA
swi_rpk_74B17_osl_sgp_std_5p_11S	CK896308	-2.10	Bd	Salmo salar zonadhesin-like gene complete cds and 3' UTR
int_oss_THN07_osl_sal_std_5p_11C	CN181376	-2.10	Bd	Salmo salar caspase 7 gene complete cds
gil_rpk_75N07_osl_sgp_std_5p_11S	CK878122	-2.10	Bd	No Hit
mus_snm_14F10_osl_tra_nrc_5p_11C	EG649168	-2.10	Bd	(P80971) Cytochrome c oxidase subunit IV isoform 2 mitochondrial precursor (EC 1.9.3.1)
ova_opk_10A02_osl_sgp_std_5p_11S	CK891089	-2.10	Bd	(P08108) Heat shock cognate 70 kDa protein (HSP70)
liv_ali_02F03_abe_tra_sub_0p_11C	AM402589	-2.10	Bd	AF394686_1C1q-like adipose specific protein
int_oss_T6O06_osl_sal_std_5p_22S	CK884079	-2.09	Bd	novel protein containing an acyltransferase domain (zgc:91857)
liv_ali_02F08_abe_tra_sub_0p_11S	AM402593	-2.09	Bd	(Q5R4N8) Alpha-2-macroglobulin precursor (Alpha-2-M)
mus_snm_03C10_sti_tra_nrc_5p_11C	EG649354	-2.09	Bd	(P04906) Glutathione S-transferase P (EC 2.5.1.18)
gil_oss_G5E22_osl_sal_std_5p_11S	CK878942	-2.08	Bd	No Hit
kid_opk_02A18_osl_sgp_std_5p_11C	CK886774	-2.08	Bd	PREDICTED: similar to methyltransferase COQ3
int_oss_T4E10_osl_sal_std_5p_11S	CK884609	-2.08	Bd	No Hit
int_oss_T6E03_osl_sal_std_5p_12C	CK884262	-2.08	Bd	microsomal glutathione S-transferase
ova_oyr_04E03_gal_sal_std_5p_11C	BM414370	-2.08	Bd	(Q92609) TBC1 domain family member 5
kid_opk_01C04_osl_sgp_std_5p_11S	CK887535	-2.07	Bd	hemopexin-like protein
tes_opk_13K24_osl_sgp_std_5p_11C	CK898763	-2.07	Bd	No Hit
mus_opk_08H05_osl_sgp_std_5p_11S	CK899722	-2.07	Bd	(P51692) Signal transducer and activator of transcription 5B
liv_ali_05D06_abe_tra_sub_0p_11S	AM412027	-2.07	Bd	No Hit
liv_opk_12G11_osl_sgp_std_5p_11C	CK889122	-2.07	Bd	unnamed protein product
mus_snm_01C01_sti_tra_nrc_5p_11C	No Acc	-2.07	Bd	(P42897) Enolase (EC 4.2.1.11) (2-phosphoglycerate dehydratase)
ova_oyr_03B05_gal_sal_std_5p_11C	No Acc	-2.07	Bd	(P56873) Sjogren's syndrome/scleroderma autoantigen 1 homolog
mus_snm_05H05_sti_tra_nrc_5p_11S	No Acc	-2.07	Bd	No Hit
swi_rpk_74C08_osl_sgp_std_5p_11C	CK896561	-2.06	Bd	Salmo salar zonadhesin-like gene complete cds and 3' UTR
kid_opk_01P23_osl_sgp_std_5p_11S	CK886730	-2.06	Bd	SSA532825Salmo salar mRNA for glutamate dehydrogenase (gdh2 gene)
kid_opk_01C06_osl_sgp_std_5p_11S	CK887547	-2.05	Bd	No Hit

liv_ali_06D05_abe_tra_sub_0p_11C	AM402904		-2.05	Bd	(Q7SXF1) 7-dehydrocholesterol reductase (EC 1.3.1.21)
mus_mfo_15F07_fou_sal_nrp_5p_11M	DW592228		-2.05	Bd	AF360980Oncorhynchus mykiss glyceraldehyde phosphate dehydrogenase
tes_opk_14I07_osl_sgp_std_5p_11C	CK897414		-2.04	Bd	No Hit
spl_sts_19B08_sti_sal_std_5p_22C	AJ425726		-2.04	Bd	novel protein
liv_opk_12I24_osl_sgp_std_5p_11C	CK887979		-2.04	Bd	(P32759) Alpha-1-antitrypsin homolog precursor
hrt_opk_04K05_osl_sgp_std_5p_11C	CK883647		-2.04	Bd	(P49263) Pentraxin fusion protein precursor
hrt_opk_05D16_osl_sgp_std_5p_11C	CK882869		-2.04	Bd	PREDICTED: similar to Cytochrome c oxidase polypeptide VIII-heart mitochondrial precursor
liv_ali_01H03_abe_tra_sub_0p_11S	AM402521		-2.04	Bd	(P36021) Monocarboxylate transporter 8 (MCT 8) (X-linked PEST-containing transporter)
int_rpk_76N08_osl_sgp_std_5p_11S	CK884925		-2.03	Bd	(Q03721) Potassium voltage-gated channel subfamily C member 4
liv_ali_04E07_abe_tra_sub_0p_11S	AM402748		-2.03	Bd	complement C4
ova_opk_11E24_osl_sgp_std_5p_11C	CK890262		-2.03	Bd	Zebrafish DNA sequence from clone DKEYP-69H1 in linkage group 17 complete sequence
mus_opk_08M03_osl_sgp_std_5p_11S	CK899445		-2.03	Bd	microtubule-associated protein 1 light chain 3
spl_opk_16N06_osl_sgp_std_5p_11S	CK894079		-2.03	Bd	No Hit
kid_sts_02B07_sti_sal_std_5p_11C	AJ424184		-2.03	Bd	No Hit
hrt_opk_08M24_osl_sgp_std_5p_11C	CK891903		-2.02	Bd	(O35343) Importin alpha-4 subunit (Karyopherin alpha-4 subunit) (Importin alpha Q1)
int_rpk_76O15_osl_sgp_std_5p_11C	CK884945		-2.02	Bd	(P56533) Betaine aldehyde dehydrogenase (EC 1.2.1.8)
mus_mfo_1aH11_fou_sal_nrp_5p_11M	DW590334		-2.02	Bd	(P79891) 40S ribosomal protein S3
int_rpk_78M24_osl_sgp_std_5p_11S	CK884485		-2.02	Bd	No Hit
hkd_opk_03K22_osl_sgp_std_5p_11C	CK880611		-2.02	Bd	Nars-prov protein
kid_opk_01C09_osl_sgp_std_5p_11C	CK887552		-2.02	Bd	(Q8R121) Protein Z-dependent protease inhibitor precursor
gil_oss_G6O07_osl_sal_std_5p_11C	CK877249		-2.01	Bd	Pagrus major putative transient receptor protein 2 mRNA partial cds
bra_snb_13F11_osl_tra_nrc_5p_11C	EG648037		-2.01	Bd	No Hit
ova_oyr_08C11_gal_sal_std_5p_12S	BM414524		-2.01	Bd	hypothetical protein LOC555268
can_HSP_S1A03_sti_tra_can_0p_12N	No Acc		-2.01	Bd	At. sal. Heat shock protein P70Contig Blast SW
tes_tsr_02G10_gal_sal_std_5p_11C	BM414155		-2.01	Bd	(P61009) Signal peptidase complex subunit 3 (EC 3.4.-.-)
mus_mfo_12H09_fou_sal_nrp_5p_11C	DW591858		-2.01	Bd	(Q02374) NADH-ubiquinone oxidoreductase AGGG subunit mitochondrial precursor (EC 1.6.5.3)
liv_ali_02G08_abe_tra_sub_0p_11C	AM402603		-2.01	Bd	(P15169) Carboxypeptidase N catalytic chain precursor (EC 3.4.17.3)
liv_opk_12G21_osl_sgp_std_5p_11C	CK889202		-2.00	Bd	(P98160) Basement membrane-specific heparan sulfate proteoglycan core protein precursor
liv_ali_05E10_abe_tra_sub_0p_11S	AM402831		-2.00	Bd	(P13255) Glycine N-methyltransferase (EC 2.1.1.20)
bra_snb_11C08_osl_tra_nrc_5p_11C	EG647829		-2.00	Bd	(O95563) Brain protein 44
mus_mfo_12C04_fou_sal_nrp_5p_11M	DW591752		-2.00	Bd	No Hit
ova_oyr_04H02_gal_sal_std_5p_11C	BM414009		-2.00	Bd	(P23787) Transitional endoplasmic reticulum ATPase (TER ATPase)
liv_lrr_01C04_gal_sal_std_5p_11C	No Acc	-4.95	-18.91	ABd	(Q02988) Lectin precursor
ova_oyr_04D02_gal_sal_std_5p_11S	BM414075	-4.93	-23.18	ABd	(Q8BTW8) CDK5 regulatory subunit associated protein 1
liv_stb_B4H09_sti_tra_sub_0p_11C	AM397495	-4.52	-6.46	ABd	(Q98SV0) Selenoprotein Pb precursor (zSelPb)

int_oss_THC12_osl_sal_std_5p_11S	CN181333	-4.33	-3.32	ABd	Oncorhynchus mykiss SYPG1 (SYPG1) PHF1 (PHF1) and RGL2 (RGL2) genes complete cds;
gil_oss_52D23_osl_sal_std_5p_11C	CK879324	-4.13	-3.19	ABd	(Q05025) Glyceraldehyde-3-phosphate dehydrogenase (EC 1.2.1.12)
ova_oyr_08G10_gal_sal_std_5p_22S	BM414533	-3.47	-9.38	ABd	hypothetical protein LOC556259
swi_rpk_74G02_osl_sgp_std_5p_11S	CK895302	-3.44	-5.01	ABd	(Q9N0V3) Bile salt export pump (ATP-binding cassette sub-family B member 11)
eye_rpk_73C12_osl_sgp_std_5p_11C	CO472191	-3.34	-2.28	ABd	No Hit
int_rpk_78H05_osl_sgp_std_5p_11S	CK886129	-3.31	-2.38	ABd	(P51469) Glyceraldehyde-3-phosphate dehydrogenase (EC 1.2.1.12) (GAPDH)
bra_cbr_B2H05_car_tra_sub_0p_11C	EG354910	-3.18	-2.24	ABd	glutamine synthetase
int_oss_T4C06_osl_sal_std_5p_11S	CK884596	-3.17	-3.02	ABd	PREDICTED: similar to zinc finger protein 420
bra_snb_10C07_osl_tra_nrc_5p_11C	EG647737	-3.14	-3.34	ABd	(Q8HZM5) Glutamine synthetase (EC 6.3.1.2) (Glutamate--ammonia ligase) (GS)
gil_oss_52N23_osl_sal_std_5p_11C	CK879377	-3.09	-2.74	ABd	(Q9N2D5) Glyceraldehyde-3-phosphate dehydrogenase (EC 1.2.1.12)
liv_dis_D4B09_abe_tra_sub_0p_11N	AM049689	-3.08	-3.93	ABd	(P32759) Alpha-1-antitrypsin homolog precursor
bra_bfo_14E08_fou_sal_nrp_5p_11S	DW590033	-3.07	-3.51	ABd	(P80534) Glyceraldehyde-3-phosphate dehydrogenase muscle (EC 1.2.1.12) (GAPDH)
liv_stb_K4G11_sti_tra_sub_0p_11C	AM397512	-3.00	-4.89	ABd	(P52786) Cytochrome P450 2J1 (EC 1.14.14.1)
liv_stb_B4E12_sti_tra_sub_0p_11C	AM397487	-2.95	-4.90	ABd	No Hit
spl_sts_13B04_sti_sal_std_5p_11C	AJ425439	-2.93	-4.83	ABd	GTP cyclohydrolase I feedback regulator
liv_opk_12G15_osl_sgp_std_5p_11C	CK889156	-2.85	-2.78	ABd	hypothetical protein LOC554114
tes_opk_15A07_osl_sgp_std_5p_11S	CK897836	-2.79	-7.95	ABd	(Q93088) Betaine--homocysteine S-methyltransferase (EC 2.1.1.5)
spl_sts_13A01_sti_sal_std_5p_11S	AJ425425	-2.78	-3.72	ABd	(O93343) GSK-3-binding protein (GBP)
mus_opk_08G21_osl_sgp_std_5p_11S	CK899665	-2.77	-2.91	ABd	glyceraldehyde 3-phosphate dehydrogenase
hrt_opk_08J05_osl_sgp_std_5p_11C	CK900108	-2.75	-4.16	ABd	(O57656) Glycerol-3-phosphate dehydrogenase [NAD+] cytoplasmic (EC 1.1.1.8)
bra_snb_13A05_osl_tra_nrc_5p_11S	EG647974	-2.73	-2.47	ABd	PREDICTED: similar to enhancer of polycomb homolog 1 isoform 2
liv_dis_D3E09_abe_tra_sub_0p_11N	AM049641	-2.70	-4.83	ABd	C-type MBL-2 protein
liv_opk_12I06_osl_sgp_std_5p_11S	CK889439	-2.68	-7.71	ABd	(P04694) Tyrosine aminotransferase (EC 2.6.1.5)
ova_oyr_08A08_gal_sal_std_5p_11S	BM414453	-2.68	-3.91	ABd	(Q00004) Signal recognition particle 68 kDa protein (SRP68)
can_GAP_S1A04_sti_tra_can_0p_12N	No Acc	-2.68	-2.85	ABd	At. sal. Glyceraldehyde phosphate dehydrogenaseContig Blast SW
mus_snm_13G03_osl_tra_nrc_5p_11C	EG649083	-2.67	-2.17	ABd	(P24774) Plasma retinol-binding protein I (PRBP-I)
tes_tsr_04B05_gal_sal_std_5p_11C	BM414275	-2.62	-3.44	ABd	PREDICTED: similar to calcium-binding protein p26olf
kid_cki_A1G02_car_tra_sub_0p_11C	No Acc	-2.62	-2.68	ABd	No Hit
can_HoD_S1A01_sti_tra_can_0p_12N	No Acc	-2.59	-2.77	ABd	At. sal. Homogenistate DioxygenaseContig Blast SW
mus_snm_14E11_osl_tra_nrc_5p_11C	EG649157	-2.58	-2.30	ABd	(Q98TT6) Eukaryotic translation initiation factor 4E-1A-binding protein
int_rpk_76H17_osl_sgp_std_5p_11S	CK884553	-2.58	-3.76	ABd	PREDICTED: similar to UDP-glucuronosyltransferase 2A1 precursor microsomal
liv_opk_12I19_osl_sgp_std_5p_11C	CK889562	-2.58	-3.26	ABd	(P32754) 4-hydroxyphenylpyruvate dioxygenase (EC 1.13.11.27)
bra_bfo_10G06_fou_sal_nrc_5p_11M	DW589535	-2.54	-2.44	ABd	(P41320) Glutamine synthetase mitochondrial precursor (EC 6.3.1.2)
mus_opk_08G14_osl_sgp_std_5p_11S	CK899620	-2.54	-3.99	ABd	unnamed protein product
can_HoD_S1B08_sti_tra_can_0p_22N	No Acc	-2.52	-2.96	ABd	At. sal. Homogenistate DioxygenaseContig Blast SW
int_rpk_78O06_osl_sgp_std_5p_11C	CK884506	-2.52	-4.66	ABd	Oncorhynchus mykiss BAC 127C24 partial sequence
bra_opk_06D07_osl_sgp_std_5p_11S	CK874533	-2.51	-2.46	ABd	Oncorhynchus mykiss SYPG1 (SYPG1) PHF1 (PHF1) and RGL2 (RGL2) genes

					complete cds;
liv_dis_D5B03_abe_tra_sub_0p_11N	AM049762	-2.51	-5.17	ABd	C-type MBL-2 protein
liv_opk_12L05_osl_sgp_std_5p_11C	CK888371	-2.47	-5.11	ABd	(P30613) Pyruvate kinase isozymes R/L (EC 2.7.1.40)
kid_opk_01L02_osl_sgp_std_5p_11S	CK887013	-2.46	-2.68	ABd	No Hit
liv_opk_12M05_osl_sgp_std_5p_11C	CK888589	-2.46	-6.49	ABd	(P31029) Serine--pyruvate aminotransferase mitochondrial precursor (EC 2.6.1.51)
kid_opk_01K02_osl_sgp_std_5p_11C	CK886937	-2.45	-3.48	ABd	(P53603) Formimidoyltransferase-cyclodeaminase (Formiminotransferase-cyclodeaminase) (FTCD)
ova_oyr_04H12_gal_sal_std_5p_11C	BM414022	-2.45	-2.42	ABd	(Q9YH91) Claudin-like protein ZF-A89 (Claudin d)
ski_opk_09G11_osl_sgp_std_5p_11S	CK892833	-2.44	-4.84	ABd	(Q16851) UTP--glucose-1-phosphate uridylyltransferase 2 (EC 2.7.7.9)
kid_sts_01C07_sti_sal_std_5p_11C	AJ424109	-2.42	-3.23	ABd	(Q4V8T0) Inositol oxygenase (EC 1.13.99.1) (Myo-inositol oxygenase)
kid_opk_01C03_osl_sgp_std_5p_11C	CK887528	-2.39	-3.29	ABd	(P32754) 4-hydroxyphenylpyruvate dioxygenase (EC 1.13.11.27)
mus_mfo_08E03_fou_sal_nrp_5p_11C	DW591204	-2.38	-2.17	ABd	OLA319580Oryzias latipes partial mRNA for cold-shock domain protein (mfYP2 gene)
int_rpk_78H18_osl_sgp_std_5p_11S	CK886207	-2.38	-3.77	ABd	(P70691) UDP-glucuronosyltransferase 1-2 precursor microsomal (EC 2.4.1.17)
liv_ali_05F09_abe_tra_sub_0p_11S	AM402849	-2.35	-2.35	ABd	(Q9JLJ4) Elongation of very long chain fatty acids protein 2
mus_snm_04D02_sti_tra_nrc_5p_11C	EG649440	-2.35	-2.72	ABd	(P00355) Glyceraldehyde-3-phosphate dehydrogenase (EC 1.2.1.12)
ova_oyr_04C10_gal_sal_std_5p_11C	No Acc	-2.35	-3.39	ABd	Homo sapiens fosmid clone XXFOS-802244A1 from 2 complete sequence
liv_opk_12K20_osl_sgp_std_5p_11S	CK888307	-2.35	-3.61	ABd	(P36514) UDP-glucuronosyltransferase 2C1 microsomal (EC 2.4.1.17)
kid_opk_01O06_osl_sgp_std_5p_11C	CK886572	-2.34	-2.20	ABd	glycogen synthase kinase binding protein
ova_oyr_08D11_gal_sal_std_5p_11C	BM414459	-2.34	-2.79	ABd	PD2-like protein
ova_oyr_07C09_gal_sal_std_5p_22C	BM414512	-2.34	-2.56	ABd	No Hit
swi_rpk_74H14_osl_sgp_std_5p_11C	CK895691	-2.33	-2.99	ABd	(P06768) Retinol-binding protein II cellular (CRBP-II)
kid_opk_01L15_osl_sgp_std_5p_11C	CK887080	-2.33	-4.75	ABd	(P53447) Fructose-bisphosphate aldolase B (EC 4.1.2.13) (Liver-type aldolase)
liv_ali_04G07_abe_tra_sub_0p_11C	AM402771	-2.32	-2.77	ABd	(Q9NYQ3) Hydroxyacid oxidase 2 (EC 1.1.3.15)
liv_ali_06F01_abe_tra_sub_0p_11C	AM402919	-2.32	-2.03	ABd	(Q92109) Cytochrome P450 1A3 (EC 1.14.14.1) (CYP1A3) (CYP1A1)
kid_sts_01C08_sti_sal_std_5p_11C	AJ424110	-2.30	-3.55	ABd	No Hit
liv_dis_D5D11_abe_tra_sub_0p_11N	AM049780	-2.29	-4.67	ABd	C-type MBL-2 protein
mus_amu_09D06_abe_tra_sub_0p_11C	AM083899	-2.28	-3.69	ABd	(Q9D0F9) Phosphoglucomutase-1 (EC 5.4.2.2)
liv_ali_01B07_abe_tra_sub_0p_11C	AM402462	-2.27	-2.81	ABd	(O88618) Formimidoyltransferase-cyclodeaminase
swi_rpk_74J19_osl_sgp_std_5p_11C	CK895950	-2.27	-2.74	ABd	No Hit
can_GAP_S1B10_sti_tra_can_0p_22N	No Acc	-2.26	-3.20	ABd	At. sal. Glyceraldehyde phosphate dehydrogenaseContig Blast SW
bra_bfo_03H05_fou_sal_nrc_5p_11M	DW588482	-2.26	-2.26	ABd	(Q9UKN8) General transcription factor 3C polypeptide 4 (EC 2.3.1.48)
tes_tsr_07H01_gal_sal_std_5p_11C	BM414199	-2.25	-2.10	ABd	No Hit
liv_opk_12F02_osl_sgp_std_5p_11C	CK888908	-2.24	-2.12	ABd	Gasterosteus aculeatus clone CEC21-F12 mRNA sequence
int_oss_T6F11_osl_sal_std_5p_11C	CK884111	-2.23	-2.06	ABd	(P19001) Keratin type I cytoskeletal 19 (Cytokeratin-19) (CK-19) (Keratin-19) (K19)
kid_opk_01K04_osl_sgp_std_5p_11C	CK886946	-2.23	-2.59	ABd	No Hit
swi_rpk_74E16_osl_sgp_std_5p_11C	CK896964	-2.22	-4.33	ABd	C1q-like adipose specific protein
liv_lrr_06A11_gal_sal_std_5p_11C	BI468091	-2.22	-2.88	ABd	PREDICTED: similar to C6orf65 protein
int_oss_T4J07_osl_sal_std_5p_11S	CK884712	-2.20	-3.00	ABd	Zebrafish DNA sequence from clone CH211-250C16 complete sequence

swi_rpk_74M05_osl_sgp_std_5p_11S	CK895001	-2.20	-4.91		ABd	isocitrate dehydrogenase 2 (NADP+) mitochondrial
ova_oyr_08B04_gal_sal_std_5p_11S	BM414522	-2.18	-3.92		ABd	No Hit
liv_ali_05H07_abe_tra_sub_0p_11S	AM402865	-2.17	-2.71		ABd	(Q99NF1) Beta beta-carotene 9' 10'-dioxygenase (EC 1.14.99.-) (Beta-carotene dioxygenase 2) (B-diox-II)
bra_snb_05D06_sti_tra_nrc_5p_11C	EG647411	-2.17	-2.29		ABd	(P14152) Malate dehydrogenase cytoplasmic (EC 1.1.1.37)
bra_snb_08E10_osl_tra_nrc_5p_11S	EG647575	-2.15	-2.64		ABd	unnamed protein product
int_rpk_76M03_osl_sgp_std_5p_11S	CK885360	-2.14	-2.96		ABd	(P07099) Epoxide hydrolase 1 (EC 3.3.2.3) (Microsomal epoxide hydrolase) (Epoxide hydratase)
liv_stb_K4E08_sti_tra_sub_0p_11C	AM397506	-2.13	-2.36		ABd	(O93477) Adenosylhomocysteinase B (EC 3.3.1.1)
spl_sts_04B06_sti_sal_std_5p_11C	AJ425086	-2.13	-3.51		ABd	(P59016) Vacuolar protein sorting 33B
liv_opk_12K24_osl_sgp_std_5p_11C	CK888340	-2.13	-2.09		ABd	(O08782) Aldose reductase-related protein 2 (EC 1.1.1.21)
eye_opk_19C10_osl_sgp_std_5p_11C	CO470176	-2.12	-2.17		ABd	Ndrg1 protein
bra_snb_05C04_sti_tra_nrc_5p_11C	EG647400	-2.12	-3.20		ABd	(Q9PVK4) L-lactate dehydrogenase B chain (EC 1.1.1.27) (LDH-B)
mus_mfo_11C04_fou_sal_nrp_5p_11C	DW591606	-2.10	-2.26		ABd	No Hit
int_rpk_76P16_osl_sgp_std_5p_11S	CK884962	-2.07	-2.36		ABd	hypothetical protein LOC405857
ova_oyr_04H09_gal_sal_std_5p_11S	BM414043	-2.07	-2.11		ABd	(Q62312) TGF-beta receptor type II precursor (EC 2.7.1.37)
ova_oyr_06B10_gal_sal_std_5p_11C	BM414426	-2.06	-3.15		ABd	PREDICTED: similar to niban protein
hrt_opk_08J24_osl_sgp_std_5p_11C	CK900270	-2.05	-2.13		ABd	AF330140Oncorhynchus mykiss myosin light chain 1 mRNA complete cds
liv_lrr_05D02_gal_sal_std_5p_11C	BI468117	-2.05	-2.43		ABd	Sepp1a protein
spl_sts_06A08_sti_sal_std_5p_11S	AJ425170	-2.03	-2.60		ABd	(Q14314) Fibroleukin precursor (Fibrinogen-like protein 2)
liv_ali_04H01_abe_tra_sub_0p_11S	AM402774	-2.03	-3.06		ABd	(O54750) Cytochrome P450 2J6 (EC 1.14.14.1)
int_rpk_76M19_osl_sgp_std_5p_11S	CK885307	-2.02	-2.15		ABd	Xenopus tropicalis finished cDNA clone TNeu136g19
gil_oss_52D01_osl_sal_std_5p_11S	CK879314	-2.02	-2.53		ABd	unnamed protein product
kid_sts_01C06_sti_sal_std_5p_11C	AJ424108	-2.02	-3.40		ABd	(P61972) Nuclear transport factor 2 (NTF-2)
int_rpk_76N07_osl_sgp_std_5p_11S	CK884924	-2.01	-2.85		ABd	(Q94F08) HIPL2 protein precursor
liv_ali_04C02_abe_tra_sub_0p_11C	AM402721	-2.00	-2.87		ABd	(O00746) Nucleoside diphosphate kinase mitochondrial precursor (EC 2.7.4.6)
	#N/A					#N/A
int_oss_T6A03_osl_sal_std_5p_11C	CK884084	-9.32		-8.66	ACd	rRNA promoter binding protein
int_rpk_78A08_osl_sgp_std_5p_11S	CK885620	-7.97		-8.17	ACd	(Q04592) Proprotein convertase subtilisin/kexin type 5 precursor (EC 3.4.21.-)
bra_snb_03H11_sti_tra_nrc_5p_11S	No Acc	-7.69		-8.65	ACd	No Hit
bra_snb_06G07_osl_tra_nrc_5p_11S	EG648153	-6.40		-6.06	ACd	No Hit
ova_opk_10D15_osl_sgp_std_5p_11C	CK891273	-5.23		-4.51	ACd	(Q18268) Phosphodiesterase delta-like protein
bra_snb_03H02_sti_tra_nrc_5p_11S	EG648401	-4.09		-3.97	ACd	PREDICTED: similar to dentin sialophosphoprotein precursor isoform 1
mus_mfo_08B05_fou_sal_nrp_5p_11M	DW591167	-3.85		-5.21	ACd	No Hit
spl_sts_17H08_sti_sal_std_5p_11C	AJ425625	-3.58		-4.97	ACd	(P63326) 40S ribosomal protein S10
swi_rpk_74E02_osl_sgp_std_5p_11S	CK896866	-3.57		-4.57	ACd	No Hit
mus_amu_01C10_abe_tra_sub_0p_11C	AM083366	-3.23		-4.04	ACd	hypothetical protein TTHERM_02141640
tes_opk_13P17_osl_sgp_std_5p_11S	CK898961	-2.99		-3.60	ACd	(Q92743) Serine protease HTRA1 precursor (EC 3.4.21.-)

liv_ali_05A01_abe_tra_sub_0p_11S	AM402785	-2.81		-2.18	ACd	(Q9R182) Angiopoietin-related protein 3 precursor (Angiopoietin-like 3)
spl_sts_20D01_sti_sal_std_5p_11C	AJ425823	-2.57		-4.75	ACd	rRNA promoter binding protein
mus_mfo_08A05_fou_sal_nrp_5p_11S	DW591157	-2.55		-2.37	ACd	(Q9DCV6) Protein KIAA0141
liv_opk_12L01_osl_sgp_std_5p_11C	CK888348	-2.49		-2.10	ACd	complement receptor-like
mus_snm_07F10_osl_tra_nrc_5p_11C	EG648557	-2.48		-3.64	ACd	putative senescence-associated protein
ova_oyr_08A02_gal_sal_std_5p_11S	BM414054	-2.45		-3.64	ACd	(Q9QZD8) Mitochondrial dicarboxylate carrier
hrt_opk_08M01_osl_sgp_std_5p_11C	CK899423	-2.39		-2.47	ACd	thioredoxin interacting protein
gil_cgi_E3H04_car_tra_sub_0p_11N	No Acc	-2.39		-2.40	ACd	(Q9DCH4) Eukaryotic translation initiation factor 3 subunit 5 (eIF-3 epsilon)
liv_lrr_06A03_gal_sal_std_5p_11C	BI468158	-2.34		-3.52	ACd	(O95477) ATP-binding cassette sub-family A member 1
int_oss_T4M01_osl_sal_std_5p_11C	CK885105	-2.33		-3.17	ACd	PREDICTED: hypothetical protein
gil_oss_G6N16_osl_sal_std_5p_11C	CK877483	-2.31		-4.15	ACd	nuclear factor kappa-B 1
liv_lrr_01H12_gal_sal_std_5p_11C	No Acc	-2.30		-2.60	ACd	Vacuolar protein sorting 37A
mus_amu_05D01_abe_tra_sub_0p_11C	AM412040	-2.28		-2.37	ACd	CHK1 checkpoint homolog (S. pombe)
liv_lrr_05A07_gal_sal_std_5p_11C	No Acc	-2.28		-2.88	ACd	(P47776) Heparin cofactor II precursor (HC-II) (Protease inhibitor leuserpin 2)
bra_bfo_06F09_fou_sal_nrc_5p_11M	DW588878	-2.24		-2.29	ACd	hypothetical 18K protein - goldfish mitochondrion
liv_ali_04G01_abe_tra_sub_0p_11S	AM402762	-2.24		-4.91	ACd	unnamed protein product
liv_opk_12K01_osl_sgp_std_5p_11C	CK888158	-2.17		-2.15	ACd	No Hit
int_oss_T6J23_osl_sal_std_5p_11C	CK884136	-2.17		-2.07	ACd	thioredoxin interacting protein
ova_oyr_07E11_gal_sal_std_5p_11S	BM414448	-2.15		-3.76	ACd	Danio rerio zgc:92668 (zgc:92668) mRNA
liv_lrr_01E09_gal_sal_std_5p_11C	No Acc	-2.13		-2.60	ACd	(Q28640) Histidine-rich glycoprotein precursor (Histidine-proline-rich glycoprotein) (HPRG) (Fragment)
tes_tsr_03A12_gal_sal_std_5p_11S	BM413916	-2.11		-3.82	ACd	(Q13227) G protein pathway suppressor 2 (GPS2 protein)
tes_tsr_02G05_gal_sal_std_5p_11C	BM414288	-2.10		-2.61	ACd	(O00750) Phosphatidylinositol-4-phosphate 3-kinase C2 domain-containing beta polypeptide (EC 2.7.1.154)
kid_sts_09E10_sti_sal_std_5p_12C	AJ424460	-2.09		-2.67	ACd	hypothetical protein LOC322350
liv_lrr_01E11_gal_sal_std_5p_11C	No Acc	-2.06		-2.42	ACd	(P70483) Striatin
liv_lrr_01C06_gal_sal_std_5p_11C	No Acc	-2.05		-2.59	ACd	(P04186) Complement factor B precursor (EC 3.4.21.47)
kid_sts_08H12_sti_sal_std_5p_11C	AJ424405	-6.09	-2.51	-4.01	ABCd	No Hit
int_rpk_78B19_osl_sgp_std_5p_11S	CK885499	-5.85	-5.28	-2.77	ABCd	No Hit
liv_ali_05C09_abe_tra_sub_0p_11S	AM402819	-5.31	-2.58	-5.09	ABCd	PREDICTED: similar to Tetratricopeptide repeat protein 14 (TPR repeat protein 14)
liv_stb_A4A03_sti_tra_sub_0p_11C	AM397478	-4.37	-2.36	-5.18	ABCd	No Hit
eye_opk_17O17_osl_sgp_std_5p_11C	CK876190	-3.62	-2.72	-3.25	ABCd	No Hit
gil_cgi_D1F04_car_tra_sub_0p_11C	EG999243	-2.79	-3.04	-2.45	ABCd	PREDICTED: similar to Nuclear protein 1 (Protein p8) (Candidate of metastasis 1) isoform 1
liv_ali_05E03_abe_tra_sub_0p_11S	AM402834	-2.59	-2.00	-3.24	ABCd	hypothetical protein LOC217071
liv_ali_05F11_abe_tra_sub_0p_11C	AM402841	-2.52	-2.00	-69.49	ABCd	(P47805) Gastrulation-specific protein G12
ova_oyr_08G01_gal_sal_std_5p_11C	BM413998	-2.19	-2.31	-2.03	ABCd	(Q9Y371) SH3 domain GRB2-like protein B1 (EC 2.3.1.-) (Endophilin B1)

liv_stb_B4B06_sti_tra_sub_0p_11C	AM397484	-2.67	-2.44		BCd	No Hit
bra_snb_12D04_osl_tra_nrc_5p_11C	EG647916	-2.34	-4.39		BCd	(Q01584) Lipocalin precursor
hrt_opk_07B03_osl_sgp_std_5p_11C	CK875077	-2.18	-3.04		BCd	Zebrafish DNA sequence from clone DKEY-89B17 in linkage group 19 complete sequence
int_oss_T5O22_osl_sal_std_5p_11C	CK885870	-2.15	-2.74		BCd	(P14527) Hemoglobin alpha-4 subunit
tes_tsr_03A03_gal_sal_std_5p_12C	BM413920	-2.13	-2.21		BCd	(Q03958) Prefoldin subunit 6 (Protein Ke2)
int_oss_T4E15_osl_sal_std_5p_11S	CK885068	-2.03	-2.11		BCd	(P14519) Serine hydroxymethyltransferase mitochondrial precursor (EC 2.1.2.1)
kid_aki_04G07_abe_tra_sub_0p_11C	AM042284			-10.17	Cd	(P81491) Serum amyloid A-5 protein
hkd_opk_03I22_osl_sgp_std_5p_11S	CK880463			-9.89	Cd	PREDICTED: similar to activating transcription factor 5
kid_aki_07B09_abe_tra_sub_0p_11S	AM042484			-8.32	Cd	immunoglobulin tau heavy chain secretory form
swi_rpk_74B22_osl_sgp_std_5p_11C	CK896319			-6.95	Cd	(Q01584) Lipocalin precursor
liv_opk_12L16_osl_sgp_std_5p_11C	CK888465			-4.82	Cd	No Hit
liv_opk_12I14_osl_sgp_std_5p_11S	CK889507			-4.65	Cd	(P80429) Serotransferrin II precursor (Siderophilin II) (STF II)
gil_oss_G6J01_osl_sal_std_5p_11C	CK877377			-4.38	Cd	CC chemokine SCYA113
bra_bfo_13G03_fou_sal_nrp_5p_11M	DW589986			-4.35	Cd	(Q92038) Acyl-CoA desaturase (EC 1.14.19.1)
gil_rpk_75A11_osl_sgp_std_5p_11C	CK879789			-4.19	Cd	Gasterosteus aculeatus clone CNB140-C06 mRNA sequence
liv_ali_05C10_abe_tra_sub_0p_11S	AM402809			-4.17	Cd	(Q07949) Probable phosphatase PSR2 (EC 3.1.3.-)
mus_snm_10F04_osl_tra_nrc_5p_11C	EG648831			-4.16	Cd	(P02142) Hemoglobin beta-1 subunit (Hemoglobin beta-1 chain)
liv_ali_04B05_abe_tra_sub_0p_11C	AM402715			-4.02	Cd	(P80429) Serotransferrin II precursor
gil_oss_52L03_osl_sal_std_5p_11C	CK879361			-3.98	Cd	thioredoxin interacting protein
ova_oyr_04F11_gal_sal_std_5p_11S	BM414013			-3.96	Cd	(P14105) Myosin-9 (Myosin heavy chain nonmuscle IIa)
gil_oss_GHK09_osl_sal_std_5p_12S	CN181226			-3.96	Cd	hypothetical protein Tc00.1047053505193.50
eye_rpk_73M16_osl_sgp_std_5p_11C	CK875442			-3.82	Cd	TTRAP_BRARETRAF and TNF receptor-associated protein homolog
swi_rpk_74F14_osl_sgp_std_5p_11C	CK895354			-3.82	Cd	unnamed protein product
gil_oss_G6I02_osl_sal_std_5p_11C	CK877297			-3.80	Cd	Parahucho perryi DNA microsatellite locus Hper-20
spl_sts_19B11_sti_sal_std_5p_12S	AJ425729			-3.71	Cd	(P02019) Hemoglobin alpha-1 subunit (Hemoglobin alpha-1 chain) (Alpha-1-globin)
gil_agi_06H07_abe_tra_sub_0p_11C	AM041897			-3.66	Cd	No Hit
int_oss_T6A14_osl_sal_std_5p_11C	CK884021			-3.66	Cd	(P62972) Ubiquitin
gil_oss_G6I01_osl_sal_std_5p_11S	CK877214			-3.65	Cd	No Hit
kid_sts_16D12_sti_sal_std_5p_11C	AJ424858			-3.64	Cd	(Q99ME9) Nucleolar GTP-binding protein 1 (Chronic renal failure gene protein)
gil_oss_G6L07_osl_sal_std_5p_11C	CK877389			-3.54	Cd	No Hit
ova_oyr_05A10_gal_sal_std_5p_11C	BM414000			-3.51	Cd	(P62916) Transcription initiation factor IIB (General transcription factor TFIIB)
tes_tsr_02B04_gal_sal_std_5p_11C	BM414132			-3.43	Cd	Oryzias latipes hox gene cluster complete cds contains hoxCa
gil_oss_G6O17_osl_sal_std_5p_11C	CK877253			-3.40	Cd	PREDICTED: similar to dapit protein
gil_rpk_74N22_osl_sgp_std_5p_11C	CK879564			-3.34	Cd	(Q9H3Y8) Protein C20orf149
gil_oss_52A01_osl_sal_std_5p_11C	CK879123			-3.33	Cd	(Q91473) Hemoglobin beta subunit
tes_tsr_03A07_gal_sal_std_5p_11C	BM413900			-3.28	Cd	(Q7ZYS1) 60S ribosomal protein L19

spl_opk_17A02_osl_sgp_std_5p_11S	CK893078	-3.27	Cd	(P11251) Hemoglobin alpha subunit (Hemoglobin alpha chain) (Alpha-globin)
gil_agi_06E04_abe_tra_sub_0p_11S	AM041862	-3.16	Cd	No Hit
gil_oss_GHD07_osl_sal_std_5p_11S	CN181195	-3.16	Cd	No Hit
gil_rpk_75N05_osl_sgp_std_5p_11S	CK878042	-3.12	Cd	SSABGLOB6S.salar genes encoding alpha-globin and beta-globin clone 6
gil_oss_GHK09_osl_sal_std_5p_22S	CN181226	-3.11	Cd	hypothetical protein Tc00.1047053505193.50
liv_lrr_01D04_gal_sal_std_5p_11C	No Acc	-3.10	Cd	(Q9R182) Angiopoietin-related protein 3 precursor (Angiopoietin-like 3)
liv_dis_D4A07_abe_tra_sub_0p_11N	AM049678	-3.09	Cd	C type lectin receptor A
eye_opk_18D09_osl_sgp_std_5p_11C	CK876469	-3.08	Cd	(P11251) Hemoglobin alpha subunit
gil_oss_G6H03_osl_sal_std_5p_11C	CK877367	-3.06	Cd	No Hit
tes_tsr_02H11_gal_sal_std_5p_11C	BM413811	-3.06	Cd	(Q8BH95) Enoyl-CoA hydratase mitochondrial precursor (EC 4.2.1.17)
bra_snb_11C01_osl_tra_nrc_5p_11C	EG647824	-3.03	Cd	(Q9H3Y8) Protein C20orf149
bra_snb_05B05_sti_tra_nrc_5p_11C	EG647391	-3.01	Cd	(P14527) Hemoglobin alpha-4 subunit (Hemoglobin alpha-4 chain) (Alpha-4-globin)
spl_sts_19C08_sti_sal_std_5p_12C	AJ425738	-2.99	Cd	PREDICTED: similar to Ubiquitin carboxyl-terminal hydrolase 4 (Ubiquitin thiolesterase 4)
liv_lrr_01C07_gal_sal_std_5p_11C	No Acc	-2.98	Cd	(Q9R182) Angiopoietin-related protein 3 precursor (Angiopoietin-like 3)
tes_tsr_02F07_gal_sal_std_5p_11C	BM413791	-2.95	Cd	(Q6RUV5) Ras-related C3 botulinum toxin substrate 1 (p21-Rac1)
mus_mfo_14H04_fou_sal_nrp_5p_11M	DW592129	-2.94	Cd	(P60060) Protein transport protein SEC61 gamma subunit
gil_oss_G6A13_osl_sal_std_5p_11S	CK877175	-2.93	Cd	STR535589Salmo trutta single nucleotide polymorphism (SNP) specimen voucher AT7-21 572BP
bra_bfo_13A12_fou_sal_nrp_5p_11C	No Acc	-2.91	Cd	No Hit
int_rpk_78B07_osl_sgp_std_5p_11C	CK885560	-2.87	Cd	(P43378) Tyrosine-protein phosphatase non-receptor type 9 (EC 3.1.3.48)
eye_opk_19L10_osl_sgp_std_5p_11S	CO470738	-2.87	Cd	unnamed protein product
gil_oss_G6L06_osl_sal_std_5p_11S	CK877470	-2.87	Cd	PREDICTED: similar to poly (ADP-ribose) polymerase family member 14
tes_tsr_03H08_gal_sal_std_5p_11C	BM414150	-2.85	Cd	(Q9NPA3) Mid1-interacting protein 1 (Gastrulation-specific G12-like protein)
bra_snb_06F02_osl_tra_nrc_5p_11C	EG647964	-2.84	Cd	(Q4AEH7) Glutathione peroxidase 2 (EC 1.11.1.9)
gil_oss_52E04_osl_sal_std_5p_11C	CK879235	-2.81	Cd	(Q91473) Hemoglobin beta subunit
gil_oss_G6G02_osl_sal_std_5p_11C	CK877288	-2.78	Cd	Ube2d2-prov protein
mus_snm_03C07_sti_tra_nrc_5p_11C	EG649361	-2.76	Cd	(O93484) Collagen alpha 2(I) chain precursor
int_rpk_78C22_osl_sgp_std_5p_11C	CK885437	-2.76	Cd	(O00423) Echinoderm microtubule-associated protein-like 1
spl_sts_19A08_sti_sal_std_5p_11S	AJ425714	-2.76	Cd	#N/AContig Blastn nr
mus_mfo_09A01_fou_sal_nrp_5p_11S	DW591257	-2.75	Cd	No Hit
spl_sts_04E11_sti_sal_std_5p_11C	AJ425127	-2.74	Cd	No Hit
gil_oss_G6M07_osl_sal_std_5p_11C	CK877238	-2.73	Cd	hypothetical protein LOC328783
gil_oss_G6N01_osl_sal_std_5p_11S	CK877398	-2.73	Cd	No Hit
ova_oya_02B02_gal_sal_std_5p_11C	BM414312	-2.72	Cd	hypothetical protein BLi00801
int_rpk_76O04_osl_sgp_std_5p_11C	CK885326	-2.71	Cd	(Q13637) Ras-related protein Rab-32
gil_oss_G6D01_osl_sal_std_5p_11C	CK877348	-2.71	Cd	PREDICTED: similar to DNA polymerase zeta catalytic subunit (hREV3) isoform 1
mus_snm_06C07_osl_tra_nrc_5p_11C	EG648715	-2.70	Cd	(Q9H3Y8) Protein C20orf149

hkd_opk_03G23_osl_sgp_std_5p_11C	CK880303	-2.70	Cd	No Hit
gil_rpk_75J23_osl_sgp_std_5p_11S	CK877824	-2.70	Cd	SSABGLOB2S.salar genes encoding alpha-globin and beta-globin clone 2
gil_oss_G5C15_osl_sal_std_5p_11S	CK878857	-2.68	Cd	(Q8WV16) WD-repeat protein 21
spl_opk_16I09_osl_sgp_std_5p_11C	CK893691	-2.68	Cd	villin 2
ova_oyr_03G06_gal_sal_std_5p_11C	BM413996	-2.68	Cd	(P08113) Endoplasmin precursor (Endoplasmic reticulum protein 99)
mus_mfo_13G09_fou_sal_nrp_5p_11S	DW591963	-2.67	Cd	(P25007) Peptidyl-prolyl cis-trans isomerase (EC 5.2.1.8)
mus_mfo_04H04_fou_sal_nrp_5p_11C	DW590695	-2.65	Cd	transposase
int_oss_THE08_osl_sal_std_5p_11S	CN181338	-2.65	Cd	IGHM enhancer 3a-binding transcription factor
liv_ali_04F09_abe_tra_sub_0p_11S	AM402761	-2.64	Cd	adenosine kinase a
mus_mfo_08C11_fou_sal_nrp_5p_11S	DW591185	-2.63	Cd	No Hit
int_rpk_78C07_osl_sgp_std_5p_11C	CK885425	-2.63	Cd	No Hit
mus_mfo_07F12_fou_sal_nrp_5p_11C	DW591113	-2.59	Cd	Zebrafish DNA sequence from clone CH211-208I16 in linkage group 18 complete sequence
spl_sts_17C07_sti_sal_std_5p_11S	AJ425579	-2.59	Cd	(Q91473) Hemoglobin beta subunit (Hemoglobin beta chain) (Beta-globin)
tes_tsa_01C01_gal_sal_std_5p_11C	No Acc	-2.58	Cd	liver-expressed antimicrobial peptide 2 isoform A precursor
tes_tsr_02G01_gal_sal_std_5p_11S	BM414144	-2.56	Cd	(Q6P7G9) Probable G-protein-coupled receptor 146
int_oss_T4M20_osl_sal_std_5p_11S	CK884656	-2.55	Cd	No Hit
kid_sts_08F09_sti_sal_std_5p_11S	AJ424379	-2.54	Cd	No Hit
spl_opk_17E09_osl_sgp_std_5p_11S	CK893379	-2.50	Cd	(Q91473) Hemoglobin beta subunit (Hemoglobin beta chain) (Beta-globin)
spl_opk_16H05_osl_sgp_std_5p_11S	CK893583	-2.48	Cd	SSGLOBINBS.salar mRNA for beta-globin
bra_bfo_14H11_fou_sal_nrp_5p_11S	No Acc	-2.47	Cd	novel protein similar to vertebrate RAD23 homolog B
spl_sts_04C02_sti_sal_std_5p_11S	AJ425094	-2.46	Cd	hypothetical protein LOC492761
bra_bfo_04H04_fou_sal_nrc_3p_11C	DW588554	-2.45	Cd	AF390021_1glutamine synthetase
liv_lrr_01A04_gal_sal_std_5p_11C	No Acc	-2.45	Cd	ADP-ribosylation factor 1 like
spl_opk_17D16_osl_sgp_std_5p_11S	CK893333	-2.44	Cd	No Hit
spl_sts_03D12_sti_sal_std_5p_11C	AJ425020	-2.43	Cd	(Q9H944) Ubiquitin-specific protease homolog 49 (TRF-proximal protein homolog)
hkd_opk_02C03_osl_sgp_std_5p_11S	CK880833	-2.42	Cd	(P09215) Protein kinase C delta type (EC 2.7.1.-) (nPKC-delta)
hkd_opk_02E06_osl_sgp_std_5p_11S	CK880988	-2.42	Cd	hypothetical protein LOC553813
gil_oss_GHO11_osl_sal_std_5p_11C	CN181242	-2.41	Cd	No Hit
bra_snb_10H09_osl_tra_nrc_5p_11C	EG647796	-2.39	Cd	CD59-like protein
pit_cpi_C1C04_car_tra_sub_0p_11C	No Acc	-2.39	Cd	glyoxalase 1
ova_oyr_01D09_gal_sal_std_5p_11C	No Acc	-2.38	Cd	chorion protein
int_oss_THP18_osl_sal_std_5p_11S	CN181464	-2.37	Cd	No Hit
gil_agi_06A11_abe_tra_sub_0p_11S	AM041820	-2.37	Cd	unnamed protein product
hkd_opk_03C15_osl_sgp_std_5p_11S	CK881845	-2.31	Cd	(P18242) Cathepsin D precursor (EC 3.4.23.5)
bra_bfo_14H12_fou_sal_nrp_5p_11S	DW590066	-2.31	Cd	TPA_exp: Oncorhynchus mykiss RTN2*1 (RTN2)
gil_rpk_75O01_osl_sgp_std_5p_11C	CK878136	-2.29	Cd	(P47198) 60S ribosomal protein L22
liv_lrr_01G04_gal_sal_std_5p_11C	No Acc	-2.29	Cd	(Q02988) Lectin precursor

gil_agi_05D07_abe_tra_sub_0p_11C	AM041777	-2.27	Cd	(P14527) Hemoglobin alpha-4 subunit (Hemoglobin alpha-4 chain)
tes_tsr_02F03_gal_sal_std_5p_11C	BM414240	-2.27	Cd	No Hit
liv_ali_04H04_abe_tra_sub_0p_11C	AM402779	-2.22	Cd	(P62193) 26S protease regulatory subunit 4 (P26s4) (Proteasome 26S subunit ATPase 1)
tes_opk_13B12_osl_sgp_std_5p_11C	CK898304	-2.20	Cd	(P54843) Transcription factor Maf (Proto-oncogene c-maf)
ova_oyr_08B05_gal_sal_std_5p_11C	No Acc	-2.20	Cd	PDZ and LIM domain 2
kid_opk_01A09_osl_sgp_std_5p_11S	CK887294	-2.18	Cd	(O54939) Estradiol 17-beta-dehydrogenase 3 (EC 1.1.1.62)
int_oss_T5A05_osl_sal_std_5p_11C	CK885697	-2.17	Cd	(P48163) NADP-dependent malic enzyme (EC 1.1.1.40) (NADP-ME) (Malic enzyme 1)
tes_opk_12N14_osl_sgp_std_5p_11C	CK898114	-2.17	Cd	SMOTC1TPSNSalmo salar transposon-like Tc1-encoded transposase pseudogene
kid_opk_01B22_osl_sgp_std_5p_11C	CK887474	-2.16	Cd	PREDICTED: hypothetical protein XP_678073 isoform 1
int_oss_T4C19_osl_sal_std_5p_11C	CK885060	-2.15	Cd	collagen a3(I)
gil_oss_52L01_osl_sal_std_5p_11S	CK879360	-2.14	Cd	(Q08509) Epidermal growth factor receptor kinase substrate 8
liv_opk_12K11_osl_sgp_std_5p_11S	CK888248	-2.14	Cd	No Hit
int_oss_THA07_osl_sal_std_5p_11C	CN181319	-2.13	Cd	Oncorhynchus mykiss COL1A3 mRNA for collagen a3(I) complete cds
mus_mfo_14E10_fou_sal_nrp_5p_11M	DW592075	-2.12	Cd	(Q9JHW0) Proteasome subunit beta type 7 precursor (EC 3.4.25.1)
kid_sts_05E08_sti_sal_std_5p_12C	AJ424303	-2.11	Cd	AF394686_1C1q-like adipose specific protein
spl_sts_05H03_sti_sal_std_5p_11C	AJ425274	-2.10	Cd	(P43305) Parvalbumin thymic CPV3 (Parvalbumin 3)
hrt_opk_08D06_osl_sgp_std_5p_11C	CK873507	-2.09	Cd	No Hit
mus_mfo_06B07_fou_sal_nrp_5p_11S	No Acc	-2.07	Cd	No Hit
mus_mfo_08H02_fou_sal_nrp_5p_11S	DW591248	-2.07	Cd	(O13085) Cytochrome c oxidase polypeptide VIa mitochondrial precursor (EC 1.9.3.1)
bra_snb_04C02_sti_tra_nrc_5p_11C	EG648433	-2.05	Cd	(P10868) Guanidinoacetate N-methyltransferase (EC 2.1.1.2)
mus_mfo_05E04_fou_sal_nrp_5p_11C	DW590783	-2.04	Cd	Zebrafish DNA sequence from clone CH211-208I16 in linkage group 18 complete sequence
mus_mfo_06F04_fou_sal_nrp_5p_11M	DW590948	-2.03	Cd	(Q00059) Transcription factor A mitochondrial precursor (mtTFA)
kid_aki_05H02_abe_tra_sub_0p_11C	AM042371	-2.03	Cd	(Q801Y3) Hepcidin 1 precursor
mus_mfo_10H08_fou_sal_nrp_5p_11M	DW591560	-2.02	Cd	No Hit
tes_opk_15G13_osl_sgp_std_5p_11C	CK897246	-2.00	Cd	(Q9HCU8) DNA polymerase delta subunit 4

Additional file 1 table S1. List of mRNAs found up and down regulated during experimental challenges those shown were significant at P<0.001 following correction for multiple tests. [1] indicates the unique code for the feature on the microarray, [2] Accession number of the cDNA sequence, if "No Acc" information is on TRAITS web page. [3] Fold change for genes increased in expression following *Aeromonas salmonicida* infection in fish fed a normal diet (AFLvsPFL). [4] Fold change for genes increased in expression following *A. salmonicida* infection in fish starved prior to infection (ASLvsPSL). [5] Fold change for genes increased in expression following 4 weeks starvation. [6] Identity of the cDNA as determined by BlastX and BlastN searches. [7] This indicates if this gene is up (u) or down (d) regulated in one or more other experiments, A (AFL), B (ASL), C (PSL), AB (AFL+ASL), ABC (AFL+ASL+PSL).
