# Supplementary material for: Starvation alters the liver transcriptome of the innate immune response in Atlantic salmon (Salmo salar)
Source: BMC Genomics. 2010 Jul 5;11:418. doi: 10.1186/1471-2164-11-418 (PMC2996946; doi:10.1186/1471-2164-11-418)
Supplement: Additional file 2 — Table S2. GO Biological processes enriched following starvation and infection. [file 1471-2164-11-418-S2.PDF]

Additional file 2 Table S2. GO Biological processes enriched following starvation and infection

GO Biological processes enriched for up regulated genes following starvation

GO Identifier[1]	Term[2]	q[3]	m[4]	p[5]
**Amino acid metabolism**				
GO:0006519	cellular amino acid and derivative metabolic process	10	296	0.05
GO:0006457	protein folding	7	130	0.01
GO:0006790	sulfur metabolic process	4	77	0.10
GO:0006518	peptide metabolic process	3	46	0.09
GO:0006749	glutathione metabolic process	3	37	0.05
GO:0006544	glycine metabolic process	3	33	0.04
**Fatty acid and steroid**				
GO:0008610	lipid biosynthetic process	6	89	0.01
GO:0006720	isoprenoid metabolic process	4	33	0.01
GO:0008299	isoprenoid biosynthetic process	4	10	0.00
GO:0006694	steroid biosynthetic process	3	21	0.01
GO:0008202	steroid metabolic process	3	43	0.08
GO:0016125	sterol metabolic process	3	18	0.01
GO:0016126	sterol biosynthetic process	3	10	0.00
**Cellular responses**				
GO:0042221	response to chemical stimulus	10	183	0.00
GO:0009605	response to external stimulus	6	124	0.05
GO:0040011	locomotion	5	52	0.01
GO:0006935	chemotaxis	4	26	0.00
GO:0007610	behavior	4	36	0.01
GO:0007626	locomotory behavior	4	27	0.00
GO:0042330	taxis	4	26	0.00
GO:0043086	negative regulation of catalytic activity	3	25	0.01
GO:0044092	negative regulation of molecular function	3	31	0.03
**Other processes**				
GO:0055080	cation homeostasis	3	47	0.10
GO:0001501	skeletal system development	4	22	0.00
GO:0043086	negative regulation of catalytic activity	3	25	0.01
GO:0001501	skeletal system development	4	22	0.00

GO Biological processes enriched for down regulated genes following starvation

GO Identity[1]	Term[2]	q[3]	m[4]	p[5]
**Blood and oxygen transport**				
GO:0015669	gas transport	10	26	0.00
GO:0015671	oxygen transport	10	26	0.00
**Lipid and steroid metabolism**				
GO:0045834	positive regulation of lipid metabolic process	3	14	0.01
GO:0006638	neutral lipid metabolic process	3	10	0.00

GO:0006639	acylglycerol metabolic process	3	10	0.00
GO:0006641	triglyceride metabolic process	3	9	0.00

Other processes

GO:0022603	regulation of anatomical structure morphogenesis	4	37	0.03
GO:0006662	glycerol ether metabolic process	3	10	0.00
GO:0018904	organic ether metabolic process	3	10	0.00
GO:0045765	regulation of angiogenesis	3	25	0.05
GO:0045766	positive regulation of angiogenesis	3	18	0.02
GO:0030334	regulation of cell migration	3	27	0.07
GO:0030335	positive regulation of cell migration	3	11	0.00
GO:0040012	regulation of locomotion	3	27	0.07
GO:0051270	regulation of cell motion	3	31	0.10
GO:0051272	positive regulation of cell motion	3	13	0.01

GO Biological processes enriched for up regulated genes following bacterial infection

GO Identifier[1]	Term[2]	q[3]	m[4]	p[5]
**Immune related**				
GO:0050896	response to stimulus	71	673	0.00
GO:0006950	response to stress	51	395	0.00
GO:0009605	response to external stimulus	33	191	0.00
GO:0006952	defense response	29	138	0.00
GO:0042221	response to chemical stimulus	29	243	0.00
GO:0009611	response to wounding	25	132	0.00
GO:0006954	inflammatory response	24	92	0.00
GO:0002376	immune system process	23	197	0.01
GO:0006955	immune response	22	161	0.00
GO:0040011	locomotion	19	77	0.00
GO:0002526	acute inflammatory response	18	45	0.00
GO:0009607	response to biotic stimulus	15	108	0.02
GO:0006953	acute-phase response	14	18	0.00
GO:0006935	chemotaxis	13	39	0.00
**cellular homeostasis**				
GO:0046907	intracellular transport	32	355	0.10
GO:0042592	homeostatic process	16	116	0.02
GO:0030003	cellular cation homeostasis	15	62	0.00
GO:0055080	cation homeostasis	15	65	0.00
GO:0048878	chemical homeostasis	15	89	0.00
GO:0019725	cellular homeostasis	15	90	0.00
GO:0006873	cellular ion homeostasis	15	78	0.00
GO:0055082	cellular chemical homeostasis	15	80	0.00
GO:0050801	ion homeostasis	15	81	0.00
GO:0030005	cellular di-, tri-valent inorganic cation homeostasis	11	52	0.00
GO:0055066	di-, tri-valent inorganic cation homeostasis	11	52	0.00
GO:0006879	cellular iron ion homeostasis	7	22	0.00
GO:0055072	iron ion homeostasis	7	22	0.00
**Protein metabolism**				
GO:0006886	intracellular protein transport	24	216	0.02
GO:0034613	cellular protein localization	24	218	0.02

GO:0006457	protein folding	22	155	0.00
GO:0006986	response to unfolded protein	12	61	0.00
GO:0051789	response to protein stimulus	12	61	0.00

Other processes

GO:0070727	cellular macromolecule localization	24	218	0.02
GO:0042330	taxis	13	39	0.00
GO:0007626	locomotory behavior	13	41	0.00
GO:0007610	behavior	13	53	0.00
GO:0048193	Golgi vesicle transport	12	87	0.06
GO:0001501	skeletal system development	9	30	0.00
GO:0048870	cell motility	7	35	0.04

GO Biological processes enriched for down regulated genes following bacterial infection

GO Identifier1	Term[2]	q[3]	m[4]	p[5]
Energy metabolism				
GO:0019752	carboxylic acid metabolic process	63	544	0.00
GO:0032787	monocarboxylic acid metabolic process	32	241	0.00
GO:0046483	heterocycle metabolic process	21	199	0.00
GO:0006749	glutathione metabolic process	6	44	0.04
GO:0005975	carbohydrate metabolic process	36	337	0.00
GO:0005996	monosaccharide metabolic process	26	166	0.00
GO:0006006	glucose metabolic process	24	140	0.00
GO:0006066	cellular alcohol metabolic process	32	212	0.00
GO:0006091	generation of precursor metabolites and energy	24	354	0.05
GO:0006096	glycolysis	18	94	0.00
GO:0019318	hexose metabolic process	26	166	0.00
GO:0044262	cellular carbohydrate metabolic process	30	258	0.00
GO:0006090	pyruvate metabolic process	18	109	0.00
GO:0006094	gluconeogenesis	17	96	0.00
GO:0016051	carbohydrate biosynthetic process	19	132	0.00
GO:0019319	hexose biosynthetic process	17	102	0.00
GO:0034637	cellular carbohydrate biosynthetic process	19	124	0.00
GO:0046165	alcohol biosynthetic process	17	102	0.00
GO:0046364	monosaccharide biosynthetic process	17	102	0.00
GO:0006081	cellular aldehyde metabolic process	5	24	0.01
GO:0046487	glyoxylate metabolic process	4	18	0.02
GO:0006586	indolalkylamine metabolic process	11	57	0.00
GO:0042430	indole and derivative metabolic process	11	58	0.00
GO:0042434	indole derivative metabolic process	11	57	0.00
GO:0006730	one-carbon compound metabolic process	6	34	0.01
GO:0046942	carboxylic acid transport	4	25	0.06

Lipid and steroid metabolism

GO:0006629	lipid metabolic process	28	306	0.00
GO:0044255	cellular lipid metabolic process	23	268	0.00
GO:0006631	fatty acid metabolic process	14	110	0.00
GO:0008610	lipid biosynthetic process	12	123	0.02
GO:0016053	organic acid biosynthetic process	11	68	0.00
GO:0008202	steroid metabolic process	8	57	0.01
GO:0006633	fatty acid biosynthetic process	7	58	0.05
GO:0006720	isoprenoid metabolic process	6	35	0.01

GO:0006694	steroid biosynthetic process	5	27	0.02
GO:0006636	unsaturated fatty acid biosynthetic process	4	12	0.00
GO:0033559	unsaturated fatty acid metabolic process	4	12	0.00
GO:0010817	regulation of hormone levels	4	18	0.02
GO:0042445	hormone metabolic process	4	17	0.01
GO:0008203	cholesterol metabolic process	4	22	0.04
GO:0016125	sterol metabolic process	4	25	0.06

Protein and amino acid metabolism

GO:0006807	nitrogen compound metabolic process	47	419	0.00
GO:0034641	cellular nitrogen compound metabolic process	45	404	0.00
GO:0009308	cellular amine metabolic process	44	397	0.00
GO:0006519	cellular amino acid and derivative metabolic process	43	381	0.00
GO:0006520	cellular amino acid metabolic process	42	366	0.00
GO:0009069	serine family amino acid metabolic process	13	147	0.04
GO:0009066	aspartate family amino acid metabolic process	13	95	0.00
GO:0009064	glutamine family amino acid metabolic process	12	70	0.00
GO:0006790	sulfur metabolic process	11	107	0.02
GO:0006575	cellular amino acid derivative metabolic process	11	84	0.00
GO:0006576	biogenic amine metabolic process	11	70	0.00
GO:0006568	tryptophan metabolic process	10	55	0.00
GO:0044271	nitrogen compound biosynthetic process	9	81	0.03
GO:0006544	glycine metabolic process	9	39	0.00
GO:0006563	L-serine metabolic process	8	36	0.00
GO:0006525	arginine metabolic process	7	41	0.01
GO:0006560	proline metabolic process	7	40	0.00
GO:0006566	threonine metabolic process	7	32	0.00
GO:0006570	tyrosine metabolic process	6	52	0.08
GO:0006547	histidine metabolic process	6	16	0.00
GO:0009075	histidine family amino acid metabolic process	6	16	0.00
GO:0006553	lysine metabolic process	5	30	0.03
GO:0019794	nonprotein amino acid metabolic process	5	23	0.01
GO:0006558	L-phenylalanine metabolic process	5	32	0.04
GO:0051171	regulation of nitrogen compound metabolic process	4	7	0.00
GO:0019482	beta-alanine metabolic process	4	19	0.02
GO:0009084	glutamine family amino acid biosynthetic process	4	17	0.01
GO:0006541	glutamine metabolic process	4	22	0.04

Catabolism

GO:0009056	catabolic process	56	685	0.00
GO:0044248	cellular catabolic process	43	453	0.00
GO:0009057	macromolecule catabolic process	30	474	0.05
GO:0044265	cellular macromolecule catabolic process	20	254	0.02
GO:0046164	alcohol catabolic process	20	122	0.00
GO:0016052	carbohydrate catabolic process	19	130	0.00
GO:0006007	glucose catabolic process	18	114	0.00
GO:0019320	hexose catabolic process	18	114	0.00
GO:0044275	cellular carbohydrate catabolic process	18	123	0.00
GO:0046365	monosaccharide catabolic process	18	114	0.00
GO:0009063	cellular amino acid catabolic process	17	88	0.00
GO:0009310	amine catabolic process	17	89	0.00
GO:0044270	nitrogen compound catabolic process	17	90	0.00
GO:0016042	lipid catabolic process	8	63	0.02
GO:0009068	aspartate family amino acid catabolic process	6	33	0.01

GO:0009074	aromatic amino acid family catabolic process	6	11	0.00
GO:0019439	aromatic compound catabolic process	6	13	0.00
GO:0042178	xenobiotic catabolic process	5	13	0.00
GO:0006554	lysine catabolic process	5	26	0.01
GO:0006559	L-phenylalanine catabolic process	5	10	0.00
GO:0008300	isoprenoid catabolic process	4	15	0.01
GO:0043694	monoterpene catabolic process	4	15	0.01
GO:0046247	terpene catabolic process	4	15	0.01
GO:0046251	limonene catabolic process	4	15	0.01

Other processes

GO:0008152	metabolic process	152	3113	0.00
GO:0044237	cellular metabolic process	128	2703	0.01
GO:0044238	primary metabolic process	127	2716	0.01
GO:0006082	organic acid metabolic process	63	545	0.00
GO:0006725	cellular aromatic compound metabolic process	24	227	0.00
GO:0042221	response to chemical stimulus	23	243	0.00
GO:0009072	aromatic amino acid family metabolic process	15	88	0.00
GO:0046394	carboxylic acid biosynthetic process	11	68	0.00
GO:0009410	response to xenobiotic stimulus	9	50	0.00
GO:0006805	xenobiotic metabolic process	9	50	0.00
GO:0000740	nuclear membrane fusion	7	8	0.00
GO:0006944	membrane fusion	7	36	0.00
GO:0006699	bile acid biosynthetic process	4	12	0.00
GO:0008206	bile acid metabolic process	4	17	0.01
GO:0018923	limonene metabolic process	4	15	0.01
GO:0042214	terpene metabolic process	4	15	0.01
GO:0043692	monoterpene metabolic process	4	15	0.01
GO:0042537	benzene and derivative metabolic process	4	27	0.08
GO:0015849	organic acid transport	4	25	0.06

GO Biological processes enriched for genes increased to a greater extent in fed infected fish than starved infected fish

GO Identifier[1]	Term[2]	q[3]	m[4]	p[5]

Immune related

GO:0009605	response to external stimulus	6	191	0.07
GO:0002252	immune effector process	4	51	0.01
GO:0002253	activation of immune response	4	31	0.00
GO:0002526	acute inflammatory response	4	45	0.01
GO:0002541	activation of plasma proteins involved in acute inflammatory response	4	27	0.00
GO:0002682	regulation of immune system process	4	53	0.01
GO:0002684	positive regulation of immune system process	4	38	0.00
GO:0006954	inflammatory response	4	92	0.06
GO:0006956	complement activation	4	27	0.00
GO:0006959	humoral immune response	4	35	0.00
GO:0048583	regulation of response to stimulus	4	63	0.02
GO:0048584	positive regulation of response to stimulus	4	37	0.00
GO:0050776	regulation of immune response	4	43	0.00
GO:0050778	positive regulation of immune response	4	34	0.00

Protein and amino acid metabolism

GO:0016485	protein processing	4	37	0.00
GO:0051604	protein maturation	4	38	0.00
GO:0051605	protein maturation by peptide bond cleavage	4	34	0.00
GO:0006986	response to unfolded protein	3	61	0.08
GO:0051789	response to protein stimulus	3	61	0.08
GO:0051171	regulation of nitrogen compound metabolic process	3	7	0.00
GO:0006790	sulfur metabolic process	4	107	0.10

GO Biological processes enriched for genes increased to a greater extent in starved infected fish than fed infected fish

GO Identifier[1]	Term[2]	q[3]	m[4]	p[5]
**Immune related**				
GO:0006950	response to stress	6	395	0.05
GO:0006952	defense response	4	138	0.02
GO:0006954	inflammatory response	3	92	0.03
GO:0009611	response to wounding	3	132	0.07
GO:0002376	immune system process	4	197	0.05
**Ion balance**				
GO:0006873	cellular ion homeostasis	3	78	0.02
GO:0019725	cellular homeostasis	3	90	0.03
GO:0030003	cellular cation homeostasis	3	62	0.01
GO:0030005	cellular di-, tri-valent inorganic cation homeostasis	3	52	0.01
GO:0042592	homeostatic process	3	116	0.05
GO:0048878	chemical homeostasis	3	89	0.03
GO:0050801	ion homeostasis	3	81	0.02
GO:0055066	di-, tri-valent inorganic cation homeostasis	3	52	0.01
GO:0055080	cation homeostasis	3	65	0.01
GO:0055082	cellular chemical homeostasis	3	80	0.02

Additional file 2 Table S2. Gene ontology (GO) identifiers enriched in the group of genes found differentially expressed by microarray. [1]Gene ontology identifier, [2]Gene Ontology term, [3]number of times the GO term is present in the gene list, [4]number of times the GO term is present on the filtered array list, [5]statistical enrichment for GO term. Only GO terms that appear 3 or more times are shown for fed / starved and 4 or more times for infection.
