# Supplementary material for: Starvation alters the liver transcriptome of the innate immune response in Atlantic salmon (Salmo salar)
Source: BMC Genomics. 2010 Jul 5;11:418. doi: 10.1186/1471-2164-11-418 (PMC2996946; doi:10.1186/1471-2164-11-418)
Supplement: Additional file 3 — Table S3. Genes altered significantly in liver by infection. [file 1471-2164-11-418-S3.PDF]

Additional file 3 Table S3. Genes altered significantly in liver following bacterial infection

TRAITS IDENTIFIER[1]	ACC[2]	AFL[3] FC A	ASL[4] FC B	group[7]	Identity[6]
**Immune related:**					
**Acute phase response**					
kid_aki_04G07_abe_tra_sub_0p_11C	AM042284	13.21	36.68	ABu	(P81491) Serum amyloid A-5 protein
liv_dis_D1E05_abe_tra_sub_0p_11N	AM049483	9.36	17.13	ABu	Differentially regulated trout protein 1
liv_lra_02F05_gal_sal_std_5p_11C	No Acc	11.90	9.04	ABu	Oncorhynchus mykiss haptoglobin mRNA partial cds
liv_dis_D4H02_abe_tra_sub_0p_11N	AM049741	14.11	7.30	ABu	Precerebellin-like protein
liv_dis_D1E12_abe_tra_sub_0p_11N	AM049479	16.18	6.74	ABu	Similar to catechol-O-methyltransferase domain containing 1
swi_rpk_74K11_osl_sgp_std_5p_11C	CK895971	5.95	3.33	ABu	(Q01528) Hemagglutinin/amebocyte aggregation factor precursor (18K-LAF)
liv_ali_04B05_abe_tra_sub_0p_11C	AM402715		3.08	Bu	(P80429) Serotransferrin II precursor (Siderophilin II) (STF II)
ova_oyr_08C10_gal_sal_std_5p_22S	BM414055	4.38	2.43	ABu	(P06238) Alpha-2-macroglobulin precursor (Alpha-2-M)
liv_dis_D4C06_abe_tra_sub_0p_11N	AM049696		2.04	Bu	(P02790) Hemopexin precursor (Beta-1B-glycoprotein)
hrt_opk_04K05_osl_sgp_std_5p_11C	CK883647		-2.04	Bd	(P49263) Pentraxin fusion protein precursor
**Antimicrobial peptides**					
kid_aki_05H02_abe_tra_sub_0p_11C	AM042371	46.22	177.73	ABu	(Q801Y3) Hepcidin 1 precursor
spl_opk_16G20_osl_sgp_std_5p_11C	CK893548	5.12	2.31	ABu	(P11941) Lysozyme C II precursor (EC 3.2.1.17) (Lysozyme type II)
**Complement factors**					
liv_ali_02G12_abe_tra_sub_0p_11C	AM402598	15.77	31.84	ABu	(P10643) Complement component C7 precursor
hrt_opk_06J17_osl_sgp_std_5p_11C	CK874795	3.39	4.27	ABu	(Q07021) Complement component 1 Q
liv_stb_J4D07_sti_tra_sub_0p_11C	AM397498	2.65		Au	(P08603) Complement factor H precursor
liv_lrr_04C03_gal_sal_std_5p_11C	BI468056	2.46		Au	(P04186) Complement factor B precursor (EC 3.4.21.47)
liv_opk_12E12_osl_sgp_std_5p_11S	CK888813	2.02		Au	(P98093) Complement C3-1
kid_sts_22D08_sti_sal_sub_0p_11C	AJ424936	-2.40		Ad	(Q811M5) Complement component C6 precursor
liv_ali_04E07_abe_tra_sub_0p_11S	AM402748		-2.03	Bd	complement C4
**Antigen presentation**					
kid_aki_07D09_abe_tra_sub_0p_11S	AM042502	52.18	124.66	ABu	ONHMHB2M *Oncorhynchus mykiss* beta-2 microglobulin mRNA
eye_opk_20I02_osl_sgp_std_5p_11S	CO471610	7.56	2.97	ABu	(P27797) Calreticulin precursor (CRP55) (Calregulin)
gil_oss_G6E21_osl_sal_std_5p_11S	CK877203	2.05		Au	*Oncorhynchus mykiss* genes MHC class I
**Immune regulation**					
liv_opk_12G04_osl_sgp_std_5p_11C	CK889070	9.78	32.12	ABu	(Q9JLF7) Toll-like receptor 5 precursor
spl_opk_15J16_osl_sgp_std_5p_11S	CK894206	11.01	12.76	ABu	chemotaxin
liv_dis_D1F07_abe_tra_sub_0p_11N	AM049496	8.19	12.15	ABu	C type lectin receptor A
kid_sts_11B02_sti_sal_std_5p_22C	AJ424600	10.63	6.16	ABu	(O88803) Leukocyte cell-derived chemotaxin 2 precursor (Chondromodulin II) (ChM-II)
int_oss_T6L01_osl_sal_std_5p_11C	CK884137	5.68	4.86	ABu	junB protein
spl_sts_03B01_sti_sal_std_5p_11C	AJ424987	2.42	4.02	ABu	(P25963) NF-kappaB inhibitor alpha
kid_sts_10E08_sti_sal_std_5p_11C	AJ424551	3.02	3.30	ABu	(P17676) CCAAT/enhancer binding protein beta (C/EBP beta) (Nuclear factor NF-IL6)
ova_oyr_05A10_gal_sal_std_5p_11C	BM414000		3.24	Bu	(P62916) Transcription initiation factor IIB (General transcription factor TFIIB)
spl_opk_16C09_osl_sgp_std_5p_11C	CK894789		2.95	Bu	interleukin 1 receptor accessory protein

hrt_opk_09B03_osl_sgp_std_5p_11C	CK892289	3.64	2.78	ABu	(P79703) Transcription factor jun-B
hrt_opk_08P01_osl_sgp_std_5p_11C	CK892065	3.84	2.62	ABu	CCAAT/enhancer-binding protein beta
ova_opk_11J11_osl_sgp_std_5p_11C	CK890485	2.63	2.57	ABu	similar to interleukin-4 receptor alpha-chain
int_oss_T4I13_osl_sal_std_5p_11C	CK885089	3.06	2.25	ABu	(P05619) Leukocyte elastase inhibitor (LEI) (Serpin B1)
spl_sts_03F02_sti_sal_std_5p_11C	AJ425034		2.12	Bu	TNF-alpha 2
ova_opk_09P23_osl_sgp_std_5p_11C	CK891086		2.03	Bu	(Q6NZ06) Interleukin enhancer-binding factor 2 homolog
swi_rpk_74J14_osl_sgp_std_5p_11C	CK896211	3.52		Au	AF483530_1VHSV-induced protein-4
gil_agi_05G12_abe_tra_sub_0p_11S	AM041801	2.28		Au	chemokine CXC-like protein
kid_sts_15C06_sti_sal_std_5p_12C	AJ424749	2.12		Au	chemokine (C-C motif) ligand 13
kid_sts_05C11_sti_sal_std_5p_11C	AJ424283	2.04		Au	Danio rerio similar to Interferon induced with helicase C domain protein 1
hkd_opk_02P24_osl_sgp_std_5p_11C	CK881672	2.05		Au	CCAAT/enhancer binding protein alpha
int_oss_T4F12_osl_sal_std_5p_11S	CK885151		-3.88	Bd	suppressor of cytokine signaling 1-like protein
gil_oss_G6N16_osl_sal_std_5p_11C	CK877483	-2.31		Ad	nuclear factor kappa-B 1
ova_oyr_04H09_gal_sal_std_5p_11S	BM414043	-2.07	-2.11	ABd	(Q62312) TGF-beta receptor type II precursor (EC 2.7.1.37)
mus_opk_08H05_osl_sgp_std_5p_11S	CK899722		-2.07	Bd	(P51692) Signal transducer and activator of transcription 5B
ova_oyr_04D02_gal_sal_std_5p_11S	BM414075	-4.93	-23.18	ABd	(Q8BTW8) CDK5 regulatory subunit associated protein 1
**Cell surface receptors**					
liv_dis_D4D03_abe_tra_sub_0p_11N	AM049704	4.72	14.50	ABu	toll-like leucine-rich repeat protein precursor
hrt_opk_04M04_osl_sgp_std_5p_11S	CK883775	3.07	2.58	ABu	activated leukocyte cell adhesion molecule
liv_dis_D1D03_abe_tra_sub_0p_11N	AM049472	2.64	3.14	ABu	C type lectin receptor B
liv_dis_D3E09_abe_tra_sub_0p_11N	AM049641	-2.70	-4.83	ABd	C-type MBL-2 protein
**Lipid metabolism**					
bra_opk_07F18_osl_sgp_std_5p_11S	CK875291	5.39		Au	(O35760) Isopentenyl-diphosphate delta-isomerase 1 (EC 5.3.3.2)
liv_opk_12J23_osl_sgp_std_5p_11C	CK888142	4.94		Au	AF232215 steroidogenic acute regulatory protein (StAR)
ova_opk_11H16_osl_sgp_std_5p_11C	CK890396	3.57	2.67	ABu	(P49924) Fatty acid-binding protein liver (L-FABP)
can_PPa_S1B12_sti_tra_can_0p_22N	No Acc	2.31	2.04	ABu	PPARa
can_ELO_S1B07_sti_tra_can_0p_11N	No Acc	-2.28		Ad	elongase
bra_bfo_03F11_fou_sal_nrc_5p_11C	DW588445		-2.40	Bd	(Q9DEX7) Delta-5/delta-6 fatty acid desaturase (EC 1.14.19.-)
bra_bfo_07B02_fou_sal_nrc_5p_11S	DW588937		-3.33	Bd	(Q9WUR2) Peroxisomal 3 2-trans-enoyl-CoA isomerase (EC 5.3.3.8)
bra_opk_07F12_osl_sgp_std_5p_11S	CK875274		-2.33	Bd	(P15650) Acyl-CoA dehydrogenase long-chain specific mitochondrial precursor
can_D5O_S1B05_sti_tra_can_0p_11N	No Acc		-2.11	Bd	delta 5 desaturase
eye_rpk_73I22_osl_sgp_std_5p_11C	CO472476		-2.48	Bd	lipoprotein lipase
hrt_opk_04O04_osl_sgp_std_5p_11C	CK883910		-2.46	Bd	Acyl-Coenzyme A dehydrogenase long chain
int_rpk_78K01_osl_sgp_std_5p_11S	CK886455		-2.33	Bd	(Q9DBM2) Peroxisomal bifunctional enzyme (PBE) (PBFE)
liv_ali_06D05_abe_tra_sub_0p_11C	AM402904		-2.05	Bd	(Q7SXF1) 7-dehydrocholesterol reductase (EC 1.3.1.21)
bra_snb_12D04_osl_tra_nrc_5p_11C	EG647916	-2.34	-4.39	Bd	(Q01584) Lipocalin precursor
**Protein metabolism:**					
**Protein synthesis**					
gil_oss_G5G11_osl_sal_std_5p_11S	CK878874	2.97		Au	(Q64337) Sequestosome-1 (Ubiquitin-binding protein p62) (STONE14)
bra_snb_05A04_sti_tra_nrc_5p_11C	EG647378	2.19		Au	(Q15008) 26S proteasome non-ATPase regulatory subunit 6

gil_rpk_75K21_osl_sgp_std_5p_11C	CK877770	2.18		Au	(P56399) Ubiquitin carboxyl-terminal hydrolase 5 (EC 3.1.2.15)
kid_sts_02A04_sti_sal_std_5p_11C	AJ424170	2.13		Au	cathepsin B preproprotein
int_oss_T5M06_osl_sal_std_5p_22C	CK885851	2.13		Au	cathepsin Y
mus_snm_08D12_osl_tra_nrc_5p_11C	EG648628	2.05		Au	(P68037) Ubiquitin-conjugating enzyme E2 L3 (EC 6.3.2.19)
ova_opk_09M10_osl_sgp_std_5p_11C	CK890911	2.04		Au	(P21670) Proteasome subunit alpha type 4 (EC 3.4.25.1)
mus_snm_12F02_osl_tra_nrc_5p_11C	EG648990		2.06	Bu	(Q9IB84) Proteasome subunit beta type 1-A (EC 3.4.25.1)
tes_tsr_04B01_gal_sal_std_5p_11C	BM413798	4.25	3.56	ABu	(P35031) Trypsin I precursor (EC 3.4.21.4)
hrt_opk_04F14_osl_sgp_std_5p_11C	CK883277	2.83	2.17	ABu	AChain A Human Ubiquitin-Conjugating Enzyme (E2) Ubch5b Wild-Type
hkd_opk_03G08_osl_sgp_std_5p_11C	CK882106	2.59	2.37	ABu	(O35593) 26S proteasome non-ATPase regulatory subunit 14
bra_bfo_08C01_fou_sal_nrc_5p_11M	DW589115	2.05	2.43	ABu	(Q9CX56) 26S proteasome non-ATPase regulatory subunit 8
ova_oyr_02D04_gal_sal_std_5p_11S	BM414061	4.13	2.09	ABu	(P97571) Calpain-1 catalytic subunit (EC 3.4.22.52)
**Protein degradation**					
liv_opk_12I09_osl_sgp_std_5p_11C	CK889466	4.43	3.28	ABu	(Q90YT6) 60S ribosomal protein L32
gil_oss_G6A23_osl_sal_std_5p_11C	CK877180	3.73	2.42	ABu	(P62909) 40S ribosomal protein S3
kid_aki_01O09_abe_tra_sub_0p_11C	AM042049	2.45	2.33	ABu	(Q9Y221) 60S ribosome subunit biogenesis protein NIP7 homolog (KD93)
mus_snm_12B12_osl_tra_nrc_5p_11S	EG648956	2.36	2.62	ABu	(Q5RA42) Eukaryotic translation initiation factor 1A X-chromosomal
spl_sts_13D09_sti_sal_std_5p_11C	AJ425467	2.36	2.73	ABu	(P56537) Eukaryotic translation initiation factor 6 (eIF-6) (B4 integrin interactor)
kid_opk_01E01_osl_sgp_std_5p_11C	CK887656	2.35	2.19	ABu	ribosomal protein L7-like 1
hkd_opk_02I04_osl_sgp_std_5p_11C	CK881242	2.20	2.11	ABu	(P68101) Eukaryotic translation initiation factor 2 subunit 1
liv_opk_12K22_osl_sgp_std_5p_11S	CK888317	3.31	2.25	ABu	(Q99PL5) Ribosome-binding protein 1 (Ribosome receptor protein) (mRRp)
**Stress response**					
tes_opk_14A02_osl_sgp_std_5p_11S	CK898981	2.12		Au	(Q8BK64) Activator of 90 kDa heat shock protein ATPase homolog 1 (AHA1)
gil_rpk_75M22_osl_sgp_std_5p_11C	CK878109	2.02		Au	heat shock 60 kD protein 1
bra_snb_06F02_osl_tra_nrc_5p_11C	EG647964		3.21	Bu	(Q4AEH7) Glutathione peroxidase 2 (EC 1.11.1.9)
kid_sts_22A04_sti_sal_sub_0p_11C	AJ424875		2.20	Bu	(P63039) 60 kDa heat shock protein mitochondrial precursor (Hsp60)
swi_rpk_74M02_osl_sgp_std_5p_11C	CK894999	10.69	8.64	ABu	(Q91YW3) DnaJ homolog subfamily C member 3
int_oss_T6P12_osl_sal_std_5p_12C	CK884243	4.90	4.25	ABu	Heat shock protein 9B
kid_opk_01H20_osl_sgp_std_5p_11S	CK887907	3.57	3.72	ABu	(P08110) Heat shock 108 kDa protein Transferrin-binding protein
ova_opk_11C15_osl_sgp_std_5p_11C	CK890160	2.74	3.93	ABu	heat shock 60 kD protein 1
int_oss_T6P12_osl_sal_std_5p_22C	CK884243	2.39	2.10	ABu	Heat shock protein 9B
bra_snb_04F11_sti_tra_nrc_5p_11C	EG647341	2.33	2.73	ABu	(P48721) Stress-70 protein mitochondrial precursor
ova_oyr_06E10_gal_sal_std_5p_22C	BM414402	2.18	2.17	ABu	AHA1 activator of heat shock 90kDa protein ATPase homolog 1 like
int_oss_T5E08_osl_sal_std_5p_11C	CK885807	2.14	2.90	ABu	(P63039) 60 kDa heat shock protein mitochondrial precursor (Hsp60)
kid_aki_05A08_abe_tra_sub_0p_11C	AM042306	29.57	12.79	ABu	(P06761) 78 kDa glucose-regulated protein precursor (GRP 78) (BiP)
**Serum and oxygen transport**					
swi_rpk_74I18_osl_sgp_std_5p_11S	CK895661	-2.81		Bd	(Q03156) Serum albumin 2 precursor
liv_ali_03C09_abe_tra_sub_0p_11S	AM402646	-2.80		Bd	(O57523) Apolipoprotein A-I-1 precursor (Apo-AI-1) (ApoA-I-1)
mus_snm_01H03_sti_tra_nrc_5p_11C	EG649260	-2.74		Bd	(P14527) Hemoglobin alpha-4 subunit
liv_ali_04C06_abe_tra_sub_0p_11C	AM402725	-2.56		Bd	(P28665) Murinoglobulin-1 precursor (MuG1)
mus_mfo_1aB10_fou_sal_nrp_5p_11M	DW590197	-2.46		Bd	(P02142) Hemoglobin beta-1 subunit

liv_opk_12K03_osl_sgp_std_5p_11C	CK888164	-2.27		Bd	(P80961) Antifreeze protein LS-12 precursor
liv_lrr_06F09_gal_sal_std_5p_11C	BI468110	-2.10		Bd	(P17690) Beta-2-glycoprotein I precursor (Apolipoprotein H)
spl_sts_13G09_sti_sal_std_5p_12C	AJ425502	-2.03		Ad	HMOX_FUGRUHeme oxygenase (HO)
liv_ali_05F02_abe_tra_sub_0p_11S	AM402843	-2.01		Ad	(P04114) Apolipoprotein B-100 precursor (Apo B-100)
**Energy and glycolysis**					
bra_bfo_07B08_fou_sal_nrc_5p_11M	DW588946		-2.19	Bd	(O42259) Glyceraldehyde-3-phosphate dehydrogenase (EC 1.2.1.12)
gil_agi_01G06_abe_tra_sub_0p_11C	AM041479		-2.38	Bd	(P20373) L-lactate dehydrogenase B chain (EC 1.1.1.27)
mus_snm_01C01_sti_tra_nrc_5p_11C	No Acc		-2.07	Bd	(P42897) Enolase (EC 4.2.1.11) (2-phosphoglycerate dehydratase)
bra_bfo_14E08_fou_sal_nrp_5p_11S	DW590033	-3.07	-3.51	ABd	(P80534) Glyceraldehyde-3-phosphate dehydrogenase muscle (EC 1.2.1.12) (
bra_snb_05C04_sti_tra_nrc_5p_11C	EG647400	-2.12	-3.20	ABd	(Q9PVK4) L-lactate dehydrogenase B chain (EC 1.1.1.27) (LDH-B)
bra_snb_05D06_sti_tra_nrc_5p_11C	EG647411	-2.17	-2.29	ABd	(P14152) Malate dehydrogenase cytoplasmic (EC 1.1.1.37)
gil_oss_52D23_osl_sal_std_5p_11C	CK879324	-4.13	-3.19	ABd	(Q05025) Glyceraldehyde-3-phosphate dehydrogenase (EC 1.2.1.12)
hrt_opk_08J05_osl_sgp_std_5p_11C	CK900108	-2.75	-4.16	ABd	(O57656) Glycerol-3-phosphate dehydrogenase [NAD+] cytoplasmic (EC 1.1.1.8)
kid_opk_01L15_osl_sgp_std_5p_11C	CK887080	-2.33	-4.75	ABd	(P53447) Fructose-bisphosphate aldolase B (EC 4.1.2.13) (Liver-type aldolase)
**Stress response**					
bra_opk_05O24_osl_sgp_std_5p_11S	CK874360		-2.18	Bd	(P08108) Heat shock cognate 70 kDa protein (HSP70)
int_oss_T4K02_osl_sal_std_5p_11C	CK884637		-3.82	Bd	(O57521) Heat shock protein HSP 90-beta
kid_sts_14A06_sti_sal_std_5p_22C	AJ424632		-2.22	Bd	(P20135) Glutathione S-transferase 1 (EC 2.5.1.18)
mus_opk_08L20_osl_sgp_std_5p_11S	CK899383		-2.32	Bd	(Q71U34) Heat shock cognate 71 kDa protein

Additional file 3 Table S3. List of selected mRNAs differentially regulated resulting from bacterial infection and grouped according to functional classes (shown in bold). The selection was based on manual assignment of function and those genes that had greatest fold change are shown, down regulated genes have (-) with fold change. In this table the order of the genes within each functional group is by decreasing expression level in the ASL group. If the genes only occur in the AFL group they are ordered by decreasing expression for AFL. The genes shown were significant at P<0.001 following correction for multiple tests and greater than 2 fold change. [1]Indicates the unique code for the feature on the microarray, [2] Accession number of the cDNA sequence, if "No Acc" the TRAITS web page. [3]Fold change for genes increased in expression following *Aeromonas salmonicida* infection in fish fed a normal diet (AFL vs PFL). [4]Fold change for genes increased in expression following *A. salmonicida* infection in fish starved prior to infection (ASL vs PSL). [5]Fold change for genes increased expression following 28 days starvation. [6]Identity of the cDNA as determined by BlastX and BlastN searches. [7]This indicates if this gene is up regulated in one or more other experiments, Ad (AFL), Bd (ASL), Cd (PSL), ABd (AFL+ASL), ABCd (AFL+ASL+PSL) . The complete list of genes can be found in Additional file 1 Table S1.
