# Supplementary material for: Starvation alters the liver transcriptome of the innate immune response in Atlantic salmon (Salmo salar)
Source: BMC Genomics. 2010 Jul 5;11:418. doi: 10.1186/1471-2164-11-418 (PMC2996946; doi:10.1186/1471-2164-11-418)
Supplement: Additional file 4 — Table S4. Full list of genes responding differently to infection between fed and starved fish. [file 1471-2164-11-418-S4.PDF]

Additional file 4 Table S4. Full list of genes responding differently to infection between fed and starved fish.

TRAITS IDENTIFIER[1]	ACC[2]	AFL/PFL[3] FC A	ASL/PSL[4] FC B	Interaction[5]	IDENTITY[6]
**Increased more in AFL than ASL**					
ova_oyr_07F12_gal_sal_std_5p_11C	BM414341	17.73	6.39	2.77	(Q9UMY4) Sorting nexin-12
liv_ali_02D09_abe_tra_sub_0p_11C	AM402572	16.23	6.86	2.37	(P55083) Microfibril-associated glycoprotein 4 precursor
liv_dis_D1E12_abe_tra_sub_0p_11N	AM049479	16.18	6.74	2.40	similar to catechol-O-methyltransferase domain containing 1
liv_dis_D3C06_abe_tra_sub_0p_11N	AM049619	16.06	6.01	2.67	(Q40313) Caffeoyl-CoA O-methyltransferase (EC 2.1.1.104)
liv_dis_D3C01_abe_tra_sub_0p_11N	AM049614	15.22	5.40	2.82	(Q40313) Caffeoyl-CoA O-methyltransferase (EC 2.1.1.104)
int_oss_T5H03_osl_sal_std_5p_11S	CK885905	13.56	3.62	3.74	Salmo salar clone Rsa459 microsatellite sequence
liv_dis_D5D03_abe_tra_sub_0p_11N	AM049783	12.53	4.78	2.62	similar to catechol-O-methyltransferase domain containing 1
mus_opk_08K02_osl_sgp_std_5p_11S	CK900277	11.92	4.74	2.51	(Q5RI56) Optineurin
swi_rpk_74D20_osl_sgp_std_5p_11S	CK896849	11.32	2.22	5.10	Oncorhynchus mykiss fushi tarazu factor 1 (FTZ-F1)
liv_lrr_03G12_gal_sal_std_5p_11C	No Acc	10.94	2.89	3.79	hypothetical protein XP_677792 isoform 1
swi_rpk_74J10_osl_sgp_std_5p_11S	CK896202	10.42	2.09	4.99	No hit
tes_tsr_02D08_gal_sal_std_5p_11C	BM414146	9.62	2.91	3.31	(Q99K41) EMILIN-1 precursor (Elastin microfibril interface-located protein 1)
eye_opk_20I02_osl_sgp_std_5p_11S	CO471610	7.56	2.97	2.54	(P27797) Calreticulin precursor (CRP55) (Calregulin) (HACBP) (ERp60)
ova_opk_10C23_osl_sgp_std_5p_11S	CK891239	6.84	2.61	2.62	(P15253) Calreticulin precursor (CRP55) (Calregulin) (HACBP) (ERp60)
hrt_opk_05J02_osl_sgp_std_5p_11S	CK882427	6.59	3.22	2.05	MGC89305 protein
mus_opk_08L10_osl_sgp_std_5p_11S	CK900534	6.26	2.70	2.32	hypothetical protein LOC386968
mus_snm_01B09_sti_tra_nrc_5p_11S	EG649209	6.00	2.77	2.17	hypothetical protein LOC563855
bra_snb_04D10_sti_tra_nrc_5p_11C	EG648439	5.81	2.46	2.36	hypothetical protein LOC563855
mus_mfo_13B12_fou_sal_nrp_5p_12M	DW591886	5.60	2.36	2.37	(P36871) Phosphoglucomutase-1 (EC 5.4.2.2)
bra_opk_07F18_osl_sgp_std_5p_11S	CK875291	5.41	1.22	4.43	(O35760) Isopentenyl-diphosphate delta-isomerase 1 (EC 5.3.3.2)
hrt_opk_05H16_osl_sgp_std_5p_11C	CK882321	5.22	2.32	2.25	hypothetical protein LOC563855
mus_mfo_13B12_fou_sal_nrp_5p_22M	DW591886	5.21	2.51	2.08	(P36871) Phosphoglucomutase-1 (EC 5.4.2.2)
eye_opk_20N21_osl_sgp_std_5p_11C	CO471940	5.09	1.29	3.96	(Q4R4W5) Isopentenyl-diphosphate delta-isomerase 1 (EC 5.3.3.2)
spl_opk_16G20_osl_sgp_std_5p_11C	CK893548	5.08	2.31	2.20	(P11941) Lysozyme C II precursor (EC 3.2.1.17)
liv_opk_12J23_osl_sgp_std_5p_11C	CK888142	4.94	1.95	2.54	AF232215Salvelinus fontinalis steroidogenic acute regulatory protein (StAR)
int_oss_T5H07_osl_sal_std_5p_11S	CK885907	4.93	*-1.07*	5.28	transposase
liv_lrr_06F01_gal_sal_std_5p_11C	No Acc	4.62	1.84	2.51	(O88803) Leukocyte cell-derived chemotaxin 2 precursor
bra_bfo_12B08_fou_sal_nrc_5p_11M	No Acc	4.19	1.33	3.15	No hit
ova_oyr_07G06_gal_sal_std_5p_11S	No Acc	4.12	1.80	2.29	CK073_XENLAProtein C11orf73 homolog
liv_dis_D2G04_abe_tra_sub_0p_11N	AM049579	3.94	1.28	3.08	chemotaxin

spl_sts_05A01_sti_sal_std_5p_11C	AJ425281	3.55	1.73	2.05	(O08623) Sequestosome-1 (Ubiquitin-binding protein p62)
spl_opk_16I18_osl_sgp_std_5p_11S	CK893722	3.54	1.77	2.01	OMLYRNAO.mykiss mRNA for lysozyme II
swi_rpk_74J14_osl_sgp_std_5p_11C	CK896211	3.50	1.61	2.18	AF483530_1VHSV-induced protein-4
liv_dis_D1B08_abe_tra_sub_0p_11N	AM049455	3.24	1.50	2.16	Danio rerio similar to myosin light chain kinase isoform 6 (LOC559667) mRNA
liv_opk_12I04_osl_sgp_std_5p_11C	CK889426	3.10	1.25	2.49	Danio rerio similar to myosin light chain kinase isoform 6 (LOC559667) mRNA
liv_stb_K4E03_sti_tra_sub_0p_11S	AM397505	3.08	*1.12*	2.74	hypothetical protein XP_683888
swi_rpk_74L20_osl_sgp_std_5p_11S	CK895060	3.07	1.39	2.21	hypothetical protein XP_683888
kid_sts_05A07_sti_sal_std_5p_12C	AJ424263	3.06	1.33	2.30	No hit
mus_mfo_08G06_fou_sal_nrp_5p_11C	DW591237	3.02	1.38	2.18	(Q61142) Spindlin (30000 Mr metaphase complex) (SSEC P)
gil_oss_G5G11_osl_sal_std_5p_11S	CK878874	2.97	1.46	2.04	(Q64337) Sequestosome-1 (Ubiquitin-binding protein p62) (STONE14)
kid_opk_01A01_osl_sgp_std_5p_11C	CK887252	2.93	*1.18*	2.47	ONU56710Oncorhynchus nerka microsatellite Oneu10 DNA
liv_stb_J4D07_sti_tra_sub_0p_11C	AM397498	2.65	*1.09*	2.43	(P08603) Complement factor H precursor (H factor 1)
ova_oyr_08E05_gal_sal_std_5p_11S	BM414527	2.49	*1.10*	2.25	unnamed protein product
liv_lrr_04C03_gal_sal_std_5p_11C	BI468056	2.46	*-1.03*	2.54	(P04186) Complement factor B precursor (EC 3.4.21.47)
ova_opk_09N20_osl_sgp_std_5p_11S	CK890974	2.37	-1.63	3.86	solute carrier family 25 member 25
spl_sts_17F07_sti_sal_std_5p_11C	AJ425602	2.31	*1.14*	2.03	(Q5RF83) Cold-inducible RNA-binding protein (Glycine-rich RNA-binding protein CIRP)
liv_dis_D2A01_abe_tra_sub_0p_11N	AM049516	2.29	*1.12*	2.05	(Q864W1) Complement factor B precursor (EC 3.4.21.47)
liv_opk_12H05_osl_sgp_std_5p_11S	CK889271	2.18	-1.20	2.62	No hit
hrt_opk_04E01_osl_sgp_std_5p_11C	CK883183	2.09	-1.43	2.99	unnamed protein product
ova_oya_01E08_gal_sal_std_5p_11C	No Acc	2.03	*-1.02*	2.06	(Q9JI19) Acidic fibroblast growth factor intracellular binding protein
liv_opk_12E12_osl_sgp_std_5p_11S	CK888813	2.02	*-1.13*	2.29	(P98093) Complement C3-1

Increased more in ASL than AFL

kid_aki_07D09_abe_tra_sub_0p_11S	AM042502	52.18	124.66	-2.39	ONHMHB2MOncorhynchus mykiss beta-2 microglobulin mRNA complete cds
kid_aki_05H02_abe_tra_sub_0p_11C	AM042371	46.53	177.59	-3.82	(Q801Y3) Hepcidin 1 precursor
swi_rpk_74F14_osl_sgp_std_5p_11C	CK895354	22.51	75.32	-3.35	unnamed protein product
liv_ali_02G12_abe_tra_sub_0p_11C	AM402598	15.77	31.84	-2.02	(P10643) Complement component C7 precursor
kid_aki_04G07_abe_tra_sub_0p_11C	AM042284	13.21	36.72	-2.78	(P81491) Serum amyloid A-5 protein
kid_aki_07B09_abe_tra_sub_0p_11S	AM042484	12.09	25.91	-2.14	immunoglobulin tau heavy chain secretory form
liv_opk_12G04_osl_sgp_std_5p_11C	CK889070	9.80	32.12	-3.28	(Q9JLF7) Toll-like receptor 5 precursor
liv_dis_D4D03_abe_tra_sub_0p_11N	AM049704	4.72	14.51	-3.07	toll-like leucine-rich repeat protein precursor
liv_opk_12D07_osl_sgp_std_5p_11C	CK888622	3.69	7.44	-2.02	(O15431) High-affinity copper uptake protein 1 (hCTR1) (Copper transporter 1)
liv_dis_D4A07_abe_tra_sub_0p_11N	AM049678	3.09	7.36	-2.38	C type lectin receptor A
ova_oyr_02C07_gal_sal_std_5p_11C	BM414412	2.95	5.98	-2.03	No hit

mus_snm_10E10_osl_tra_nrc_5p_11C	EG648827	2.14	5.06	-2.36	Gasterosteus aculeatus clone CNB269-C04 mRNA sequence
spl_opk_16E18_osl_sgp_std_5p_11S	CK894917	1.95	4.69	-2.40	Salmo salar BAC S0188I22 partial sequence
ova_oyr_04F11_gal_sal_std_5p_11S	BM414013	1.35	4.09	-3.02	(P14105) Myosin-9 (Myosin heavy chain nonmuscle IIa)
spl_sts_13D10_sti_sal_std_5p_11C	AJ425468	1.34	2.81	-2.10	Oncorhynchus tshawytscha insulin-like growth factor I (IGF-I.1) gene intron D
liv_ali_04F09_abe_tra_sub_0p_11S	AM402761	1.30	2.78	-2.13	adenosine kinase a
liv_opk_12L16_osl_sgp_std_5p_11C	CK888465	1.29	4.66	-3.62	No hit
hkd_opk_03G12_osl_sgp_std_5p_11S	CK880278	1.28	3.44	-2.70	No hit
bra_snb_14H07_osl_tra_nrc_5p_11C	EG648148	1.23	2.65	-2.15	(P62155) Calmodulin (CaM)
liv_ali_04B05_abe_tra_sub_0p_11C	AM402715	1.22	3.08	-2.51	(P80429) Serotransferrin II precursor
ova_oyr_05A10_gal_sal_std_5p_11C	BM414000	1.19	3.24	-2.73	(P62916) Transcription initiation factor IIB (General transcription factor TFIIB)
tes_opk_12M22_osl_sgp_std_5p_11S	CK898088	1.18	2.72	-2.31	CNS0G2P6Tetraodon nigroviridis full-length cDNA
liv_opk_12I14_osl_sgp_std_5p_11S	CK889507	1.15	4.40	-3.82	(P80429) Serotransferrin II precursor (Siderophilin II) (STF II)
bra_snb_06F02_osl_tra_nrc_5p_11C	EG647964	1.12	3.21	-2.87	(Q4AEH7) Glutathione peroxidase 2 (EC 1.11.1.9)
liv_ali_05C10_abe_tra_sub_0p_11S	AM402809	1.08	3.18	-2.93	(Q07949) Probable phosphatase PSR2 (EC 3.1.3.-)
ova_oyr_02C01_gal_sal_std_5p_11C	BM413951	-1.02	2.21	-2.26	ZPC2
**Decreased more in ASL than AFL**					
swi_rpk_74F07_osl_sgp_std_5p_11C	CK896920	2.00	-2.21	4.42	(Q9ES30) Complement C1q tumor necrosis factor-related protein 3 precursor
swi_rpk_74D18_osl_sgp_std_5p_11C	CK896717	1.41	-2.43	3.42	No hit
liv_ali_06E06_abe_tra_sub_0p_11S	AM402915	1.17	-2.20	2.57	hypothetical protein LOC447807
ova_oyr_06A10_gal_sal_std_5p_11S	BM414424	1.16	-1.81	2.11	No hit
int_rpk_76J09_osl_sgp_std_5p_11C	CK884572	1.14	-2.18	2.49	CNS0F44ATetraodon nigroviridis full-length cDNA
bra_snb_12A03_osl_tra_nrc_5p_11C	EG647882	1.14	-2.18	2.48	(P52756) RNA-binding protein 5 (RNA-binding motif protein 5)
ova_oyr_07G10_gal_sal_std_5p_22C	BM414407	1.10	-2.23	2.46	AF256963Salmo salar clone BHMS413 microsatellite sequence
mus_opk_08L20_osl_sgp_std_5p_11S	CK899383	1.08	-2.32	2.52	(Q71U34) Heat shock cognate 71 kDa protein (Heat shock 70 kDa protein 8)
kid_sts_15E04_sti_sal_std_5p_11C	AJ424770	1.06	-2.24	2.39	No hit
liv_lrr_06F09_gal_sal_std_5p_11C	BI468110	1.05	-2.10	2.20	(P17690) Beta-2-glycoprotein I precursor (Apolipoprotein H) (Apo-H)
bra_bfo_11D09_fou_sal_nrc_5p_11C	DW589613	-1.01	-2.11	2.09	(Q9UMY4) Sorting nexin-12
bra_bfo_12B09_fou_sal_nrc_5p_11M	DW589715	-1.02	-2.16	2.12	(Q99L27) GMP reductase 2 (EC 1.7.1.7)
swi_rpk_74L03_osl_sgp_std_5p_11C	CK896260	-1.03	-2.29	2.23	No hit
liv_opk_12D08_osl_sgp_std_5p_11C	CK888627	-1.07	-2.45	2.29	CGOAVACA16Coregonus artedii DNA dispersed repeat AvaIII clone Ava(CAr)-1507
bra_bfo_09D07_fou_sal_nrc_5p_11S	No Acc	-1.09	-2.26	2.07	No hit
liv_ali_03F06_abe_tra_sub_0p_11S	AM412024	-1.10	-2.62	2.39	No hit
liv_opk_12I22_osl_sgp_std_5p_11C	CK889576	-1.13	-2.38	2.11	hypothetical protein LOC492516
tes_opk_14L10_osl_sgp_std_5p_11C	CK897577	-1.16	-2.35	2.03	(P24722) Creatine kinase testis isozyme (EC 2.7.3.2)

swi_rpk_74K12_osl_sgp_std_5p_11C	CK895973	-1.19	-3.11	2.61	betaine-homocysteine methyltransferase
bra_bfo_09C03_fou_sal_nrc_5p_11S	DW589277	-1.19	-2.45	2.05	(Q15262) Receptor-type tyrosine-protein phosphatase kappa precursor (EC 3.1.3.48)
ova_oyr_08A01_gal_sal_std_5p_11C	BM414030	-1.21	-9.54	7.91	(Q9Z2N8) Actin-like protein 6A (53 kDa BRG1-associated factor A)
kid_sts_14A06_sti_sal_std_5p_12C	AJ424632	-1.21	-2.65	2.18	(P20135) Glutathione S-transferase 1 (EC 2.5.1.18)
liv_ali_05C12_abe_tra_sub_0p_11C	AM402811	-1.23	-2.69	2.19	(P42357) Histidine ammonia-lyase (EC 4.3.1.3)
ova_oyr_08D12_gal_sal_std_5p_12C	BM414031	-1.24	-2.92	2.36	(P10949) Ras-related protein Rab-3C (SMG P25C)
liv_opk_12K10_osl_sgp_std_5p_11C	CK888239	-1.26	-3.66	2.90	No hit
swi_rpk_74J11_osl_sgp_std_5p_11C	CK896114	-1.31	-4.46	3.41	AF055439Oncorhynchus kisutch microsatellite OKi14 DNA
bra_snb_02F08_sti_tra_nrc_5p_11C	EG648298	-1.31	-2.65	2.02	(P47954) Glutathione S-transferase P (EC 2.5.1.18)
liv_ali_05B07_abe_tra_sub_0p_11C	AM402805	-1.36	-3.40	2.49	similar to IGFALS
spl_sts_17G04_sti_sal_std_5p_11C	AJ425609	-1.47	-4.02	2.74	(P04431) Ig kappa chain V-I region Walker precursor
ova_oyr_08H09_gal_sal_std_5p_11S	BM414382	-1.47	-3.24	2.21	(Q9UJW8) Zinc finger protein 180 (HHZ168)
liv_opk_12H07_osl_sgp_std_5p_11S	CK889304	-1.50	-3.33	2.23	(O95685) Protein phosphatase 1 regulatory subunit 3D
liv_opk_12G17_osl_sgp_std_5p_11S	CK889175	-1.59	-3.55	2.23	No hit
liv_ali_02G01_abe_tra_sub_0p_11C	AM402595	-1.63	-3.76	2.31	C-type MBL-2 protein
ova_opk_09N23_osl_sgp_std_5p_11C	CK890982	-1.67	-4.11	2.46	(P49638) Alpha-tocopherol transfer protein (Alpha-TTP)
int_oss_T4F12_osl_sal_std_5p_11S	CK885151	-1.67	-3.88	2.32	suppressor of cytokine signaling 1-like protein
swi_rpk_74J22_osl_sgp_std_5p_11C	CK896123	-1.69	-3.88	2.29	similar to Elongation of very long chain fatty acids protein 2
hrt_opk_08H03_osl_sgp_std_5p_11C	CK899703	-1.69	-3.43	2.03	hypothetical protein LOC553776
liv_ali_05D12_abe_tra_sub_0p_11C	AM402823	-1.72	-3.77	2.20	(P21643) Tryptophan 2 3-dioxygenase (EC 1.13.11.11)
swi_rpk_74L09_osl_sgp_std_5p_11C	CK896176	-1.73	-3.83	2.21	No hit
int_oss_T4K02_osl_sal_std_5p_11C	CK884637	-1.81	-3.82	2.10	(O57521) Heat shock protein HSP 90-beta
swi_rpk_74M05_osl_sgp_std_5p_11S	CK895001	-2.20	-4.91	2.23	isocitrate dehydrogenase 2 (NADP+) mitochondrial
liv_dis_D5D11_abe_tra_sub_0p_11N	AM049780	-2.30	-4.67	2.03	C-type MBL-2 protein
kid_opk_01L15_osl_sgp_std_5p_11C	CK887080	-2.33	-4.75	2.04	(P53447) Fructose-bisphosphate aldolase B (EC 4.1.2.13)
liv_opk_12M05_osl_sgp_std_5p_11C	CK888589	-2.46	-6.49	2.64	(P31029) Serine--pyruvate aminotransferase mitochondrial precursor (EC 2.6.1.51)
liv_opk_12L05_osl_sgp_std_5p_11C	CK888371	-2.47	-5.16	2.09	(P30613) Pyruvate kinase isozymes R/L (EC 2.7.1.40)
liv_dis_D5B03_abe_tra_sub_0p_11N	AM049762	-2.51	-5.17	2.06	C-type MBL-2 protein
liv_opk_12I06_osl_sgp_std_5p_11S	CK889439	-2.68	-7.71	2.88	(P04694) Tyrosine aminotransferase (EC 2.6.1.5)
tes_opk_15A07_osl_sgp_std_5p_11S	CK897836	-2.79	-7.95	2.85	(Q93088) Betaine--homocysteine S-methyltransferase (EC 2.1.1.5)
ova_oyr_08G10_gal_sal_std_5p_22S	BM414533	-3.47	-9.38	2.70	hypothetical protein LOC556259
ova_oyr_04D02_gal_sal_std_5p_11S	BM414075	-4.93	-23.18	4.71	(Q8BTW8) CDK5 regulatory subunit associated protein 1
liv_lrr_01C04_gal_sal_std_5p_11C	No Acc	-4.95	-18.85	3.80	(Q02988) Lectin precursor

Decreased more in AFL than ASL

liv_lrr_01E11_gal_sal_std_5p_11C	No Acc	-2.06	1.38	-2.85	(P70483) Striatin
liv_lrr_01C06_gal_sal_std_5p_11C	No Acc	-2.09	1.40	-2.93	(P04186) Complement factor B precursor (EC 3.4.21.47)
tes_tsr_02G05_gal_sal_std_5p_11C	BM414288	-2.10	1.44	-3.03	(O00750) Phosphatidylinositol-4-phosphate 3-kinase (EC 2.7.1.154)
tes_tsr_03A12_gal_sal_std_5p_11S	BM413916	-2.11	1.92	-4.06	(Q13227) G protein pathway suppressor 2 (GPS2 protein)
liv_lrr_01E09_gal_sal_std_5p_11C	No Acc	-2.13	1.40	-2.98	(Q28640) Histidine-rich glycoprotein precursor (Histidine-proline-rich glycoprotein)
ova_oyr_07E11_gal_sal_std_5p_11S	BM414448	-2.16	1.17	-2.52	Danio rerio zgc:92668 (zgc:92668) mRNA
bra_bfo_06F09_fou_sal_nrc_5p_11M	DW588878	-2.24	*1.01*	-2.26	hypothetical 18K protein - goldfish mitochondrion
mus_amu_05D01_abe_tra_sub_0p_11C	AM412040	-2.31	*-1.05*	-2.19	CHK1 checkpoint homolog (S. pombe)
liv_lrr_01H12_gal_sal_std_5p_11C	No Acc	-2.31	*1.20*	-2.77	Vacuolar protein sorting 37A
gil_oss_G6N16_osl_sal_std_5p_11C	CK877483	-2.31	1.40	-3.23	nuclear factor kappa-B 1
int_oss_T4M01_osl_sal_std_5p_11C	CK885105	-2.33	1.43	-3.32	hypothetical protein
liv_lrr_06A03_gal_sal_std_5p_11C	BI468158	-2.34	1.18	-2.77	(O95477) ATP-binding cassette sub-family A member 1
ova_oyr_08A02_gal_sal_std_5p_11S	BM414054	-2.46	1.17	-2.87	(Q9QZD8) Mitochondrial dicarboxylate carrier
mus_snm_07F10_osl_tra_nrc_5p_11C	EG648557	-2.48	*1.08*	-2.67	putative senescence-associated protein
spl_sts_20D01_sti_sal_std_5p_11C	AJ425823	-2.54	*1.09*	-2.77	rRNA promoter binding protein
kid_aki_07E09_abe_tra_sub_0p_11C	AM042513	-2.64	*-1.02*	-2.59	No hit
liv_ali_05A01_abe_tra_sub_0p_11S	AM402785	-2.81	*-1.04*	-2.69	(Q9R182) Angiopoietin-related protein 3 precursor (Angiopoietin-like 3)
mus_amu_01C10_abe_tra_sub_0p_11C	AM083366	-3.23	*-1.24*	-2.60	hypothetical protein TTHERM_02141640
spl_sts_17H08_sti_sal_std_5p_11C	AJ425625	-3.60	*-1.07*	-3.37	(P63326) 40S ribosomal protein S10
mus_mfo_08B05_fou_sal_nrp_5p_11M	DW591167	-3.85	*-1.07*	-3.58	No hit
bra_snb_03H02_sti_tra_nrc_5p_11S	EG648401	-4.09	-1.31	-3.12	similar to dentin sialophosphoprotein precursor isoform 1
ova_opk_10D15_osl_sgp_std_5p_11C	CK891273	-5.23	*-1.14*	-4.60	(Q18268) Phosphodiesterase delta-like protein
liv_ali_05C09_abe_tra_sub_0p_11S	AM402819	-5.32	-2.55	-2.09	similar to Tetratricopeptide repeat protein 14 (TPR repeat protein 14)
bra_snb_06G07_osl_tra_nrc_5p_11S	EG648153	-6.40	*-1.27*	-5.06	No hit
bra_snb_03H11_sti_tra_nrc_5p_11S	No Acc	-7.67	*-1.36*	-5.62	No hit
int_rpk_78A08_osl_sgp_std_5p_11S	CK885620	-7.97	-1.40	-5.69	(Q04592) Proprotein convertase subtilisin/kexin type 5 precursor (EC 3.4.21.-)
int_oss_T6A03_osl_sal_std_5p_11C	CK884084	-9.32	*-1.27*	-7.36	rRNA promoter binding protein

Additional file 4 Table S4. List of mRNAs found to respond to the bacterial infection in a differential manner depending on prior feeding regime. The genes were differentially expressed with P<0.001 following correction for multiple tests. Only those with a two fold difference between AFL and ASL in response are shown. [1]Indicates the unique identifier for the microarray clone on the microarray, [2]Accession number of the sequence deposited to GenBank, when "No Acc" the sequence can be obtained from the TRAITS web page as described in materials. [3]Fold change in gene expression between diseased fed and uninfected fed (numbers in italics indicate they are non significant at P<0.001). [4]Fold change in gene expression between diseased starved and uninfected starved (numbers in italics indicate they are non significant at P<0.001). [5]The fold difference in the response to the disease challenge as result of the prior feeding regime. [6]Identity of the cDNAs following BlastX or BlastN.
